# A molecular atlas of the developing ectoderm defines neural, neural crest, placode, and nonneural progenitor identity in vertebrates

**DOI:** 10.1371/journal.pbio.2004045

**Published:** 2017-10-19

**Authors:** Jean-Louis Plouhinec, Sofía Medina-Ruiz, Caroline Borday, Elsa Bernard, Jean-Philippe Vert, Michael B. Eisen, Richard M. Harland, Anne H. Monsoro-Burq

**Affiliations:** 1 Université Paris Sud, Université Paris Saclay, CNRS UMR 3347, INSERM U1021, Orsay, France; 2 Institut Curie Research Division, PSL Research University, CNRS UMR 3347, INSERM U1021, Orsay, France; 3 MINES ParisTech, PSL Research University, CBIO - Centre for Computational Biology, Paris, France; 4 Department of Molecular and Cell Biology, Division of Genetics, Genomics and Development Biology, University of California, Berkeley, Berkeley, California, United States of America; 5 Institut Curie, INSERM U900, Paris, France; 6 INSERM U900, Paris, France; 7 Howard Hughes Medical Institute, University of California, Berkeley, Berkeley, California, United States of America; 8 Institut Universitaire de France, Paris, France; The Francis Crick Institute, United Kingdom of Great Britain and Northern Ireland

## Abstract

During vertebrate neurulation, the embryonic ectoderm is patterned into lineage progenitors for neural plate, neural crest, placodes and epidermis. Here, we use *Xenopus laevis* embryos to analyze the spatial and temporal transcriptome of distinct ectodermal domains in the course of neurulation, during the establishment of cell lineages. In order to define the transcriptome of small groups of cells from a single germ layer and to retain spatial information, dorsal and ventral ectoderm was subdivided along the anterior-posterior and medial-lateral axes by microdissections. Principal component analysis on the transcriptomes of these ectoderm fragments primarily identifies embryonic axes and temporal dynamics. This provides a genetic code to define positional information of any ectoderm sample along the anterior-posterior and dorsal-ventral axes directly from its transcriptome. In parallel, we use nonnegative matrix factorization to predict enhanced gene expression maps onto early and mid-neurula embryos, and specific signatures for each ectoderm area. The clustering of spatial and temporal datasets allowed detection of multiple biologically relevant groups (e.g., Wnt signaling, neural crest development, sensory placode specification, ciliogenesis, germ layer specification). We provide an interactive network interface, EctoMap, for exploring synexpression relationships among genes expressed in the neurula, and suggest several strategies to use this comprehensive dataset to address questions in developmental biology as well as stem cell or cancer research.

## Author summary

Vertebrate embryo germ layers become progressively regionalized by evolutionarily conserved molecular processes. Catching the early steps of this dynamic spatial cell diversification at the scale of the transcriptome was challenging, even with the advent of efficient RNA sequencing. We have microdissected complementary and defined areas of a single germ layer, the developing ectoderm, and explored how the transcriptome changes over time and space in the ectoderm during the differentiation of frog epidermis, neural plate, and neural crest. We have created EctoMap, a searchable interface using these regional transcriptomes, to predict the expression of the 31 thousand genes expressed in neurulae and their networks of co-expression, predictive of functional relationships. Through several examples, we illustrate how these data provide insights in development, cancer, evolution and stem cell biology.

## Introduction

Neural induction and neurulation are the critical molecular and morphogenetic processes that initiate spatial regionalization in the outer germ layer of vertebrate embryos, the ectoderm. The ectoderm forms the protective epidermis, the central nervous system, including the brain and spinal cord, as well as the neural crest (NC) cells and the sensory placodes. NC cells emerge from the border of the neural plate (NP) and generate multiple derivatives, including the peripheral nervous system, pigment cells, endocrine cells, and craniofacial structures [1]. The placodes are specialized ectodermal thickenings arising around the anterior neural plate (NPa), which form sensory organs, including lens, inner ear, cranial ganglia, and olfactory epithelium [2]. Diversification of ectodermal fates is initiated during gastrulation and early neurulation by signaling interactions that take place within the ectoderm or between ectoderm and mesoderm [3]. These developmental processes are highly conserved among vertebrates and have been intensively explored in frog embryos [4]. However, the cellular and signaling mechanisms that underlie progressive fate restriction and ectoderm patterning remain only partially understood. Moreover, besides the activation of key transcription factors in response to signaling molecules, the potential involvement of other cellular processes such as cell cycle regulation, cell metabolism, or structural cell organization remains largely unexplored during early embryo regionalization [5,6].

Development of transcriptome analyses using small samples has stimulated genome-wide studies of transcription patterns in early embryos. Several recent studies have provided transcriptomes for cleavage-stage or gastrulating embryos, either using whole embryos (WEs) or more defined spatial areas by combining embryo sectioning and spatial reconstruction (e.g., zebrafish, mouse, *Drosophila*, and frog) [5,7–10]. For early stages, these studies have reconstructed coarse-grained 3D gene expression patterns and identified important developmental events such as the transition between pluripotent epiblast state and onset of germ layer specification [11]. More recently, single-cell transcriptome analysis was applied to define cell clusters in gastrula-stage fish embryo and correlated with reference gene expression patterns [12]. As a necessary complement to these 2 approaches, the genome-wide molecular annotation of regionalized embryonic domains within a single germ layer is critical to understand gene regulation in space and time. Moreover, as previous analyses have mainly focused on the cleavage and gastrulation stages, little is currently available for the neurulation and early organogenesis steps.

We therefore aimed to document the spatial and temporal transcriptome of discrete cell populations within the ectoderm germ layer at anatomically defined locations using the frog neurula at critical stages of its development: at the end of gastrulation, as the dorsal ectoderm responds robustly to neural induction; and then during regional specification into neural, nonneural, NC, and placodal domains, at 2 later stages. At the early stages, prior to definitive cell fate specification, there is no method to sort out these small ectoderm cell populations based on a single specific gene expression; instead, to define each area, several partially overlapping regional markers must be used. We therefore used a microdissection strategy, guided by the expression of key transcription factors to define and dissect out 7 small, distinct, and complementary ectodermal domains from single embryos to provide their individual transcriptomes. The individually collected samples enable us to provide the genome-wide transcriptome profile of the anterior and posterior subdivisions of the NP and neural border (NB), the anterior neural fold (ANF) and preplacodal ectoderm (PPE), and the nonneural ectoderm (NNE). Additionally, transcriptomes for WEs at 7 stages of neurulation define a time course of gene expression and link our work to time courses of gene expression in *Xenopus laevis* and to previous analysis in *X*. *tropicalis* and other vertebrate embryos [5,7,13–16]. Using the latest *X*. *laevi*s genome assembly [16] and extensive reannotation of genes to match standard human nomenclature (human gene ortholog [HUGO]) names, included in *X*. *laevis* Annotation 9.1/Gene Model Annotation 1.8.3; Xenbase.org), we use 3 complementary tools to analyze the data and provide a user-friendly and integrated web application called "EctoMap" (for ectodermal map of differential gene expression) to visualize and explore them. EctoMap is a molecular atlas defining the regional code of ectoderm development and a searchable interface to compare gene expression levels, synexpression groups, and relationships between genes. This tool will be broadly useful in embryos and evo-devo analyses, for example, to analyze and compare gene regulatory networks (GRNs) in the evolution of NC and placodes or to study gene subfunctionalization after genome duplication, as in the *X*. *laevis* genome. EctoMap and the tools provided here constitute a spatial reference for single cell transcriptome analysis and for comparing gene expression in embryos with other contexts, such as cancer cells, stem cells, or in congenital diseases.

## Results

### Transcriptional profiling of the developing ectoderm at late gastrulation and neurulation stage

**Fig 1:**
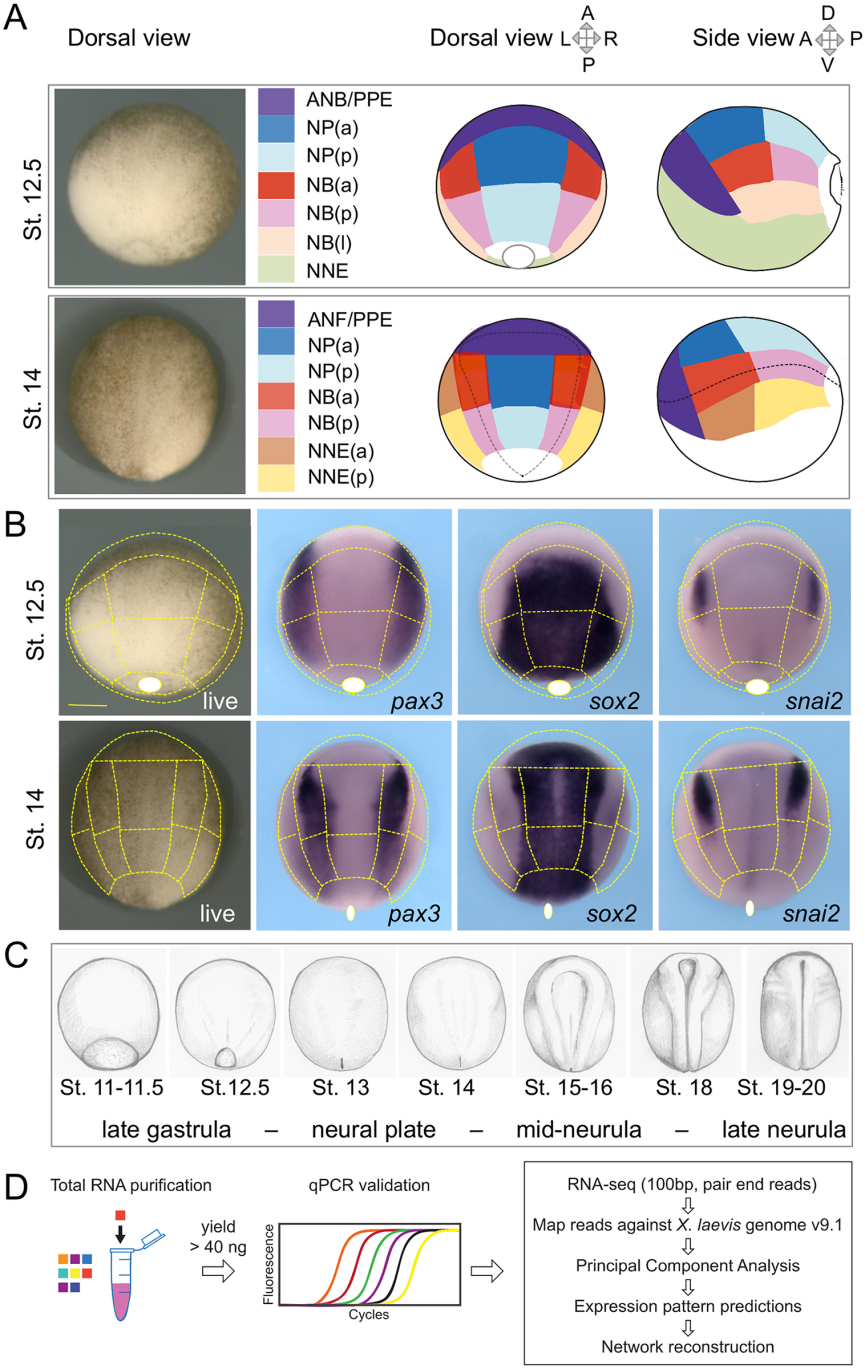
Ectoderm microdissections and experimental strategy. (A) *Xenopus laevis* neurula-stage embryos were dissected at Nieuwkoop and Faber stage (St.) 12.5 and St. 14 according to the patterns indicated. Live embryos are shown in dorsal view. The superficial ectoderm was separated from the underlying mesoderm germ layer. The tissues collected corresponded to the developing neural plate (NP, blue), medial and lateral neural border (NB, red-pink-beige), anterior neural border and preplacodal ectoderm (ANB/PPE or ANF/PPE, violet), and nonneural ectoderm (NNE, green, yellow-brown). NP, NB (at St. 12.5 and St. 14), and NNE (at St. 14 only) were further subdivided into 2 or 3 pieces: anterior (a, darker color) and posterior (p, lighter color) fragments, as indicated. For St. 12.5 NB only, a more lateral fragment was cut (l, beige), (lateral refers to medial-lateral coordinates on the dorsal view or corresponds to more ventral if using dorsal-ventral whole embryo coordinates). Each sample is associated to 1 color throughout this study (color code reference in S1 Table). (B) This dissection pattern (dotted lines) matches with reference genes expression patterns in the developing neurula ectoderm as indicated for *pax3* (dorsal-lateral NP and NB), *sox2* (NP and St. 14 PPE), and *snail2* (premigratory neural crest within the NB). The dissection line is indicated outside the embryo whenever the dissection encompasses tissues located more ventrally than seen in dorsal view (e.g., for PPE and NNE). Similarly, the blastopore (white circle) can be indicated shifted outside the embryo if located more ventrally. (C) Whole embryos at each stage of neurulation were used for studying the time course of gene expression in neurulas. Drawing after living embryos. (D) The main steps of sample treatments and bioinformatics analysis are indicated. After total RNA purification from each individual dissected sample or whole embryo, quality was checked using quantitative PCR. Suitable samples (see text) were processed for deep transcriptome sequencing. Details of analysis workflow are indicated in Results and Materials and methods.

To capture how the dorsal ectoderm progressively differentiates into multiple distinct regions, we divided the dorsal ectoderm into 7 defined areas at early and mid-neurula stages using *X*. *laevis* embryos (Fig 1A, S1 Table). The choice of these areas was guided by the current knowledge of ectoderm regionalization and corresponded to the main prospective regions (NP, epidermis, NB or NC, and ectodermal placodes). Some regions were sufficiently large to allow further subdivision into their dorsal-ventral (D-V) or anterior-posterior (A-P) portions. The first stage captures the transition between gastrulation and neurulation at Nieuwkoop and Faber stage (St.) 12.5 [17]. At this time, the ectoderm is classically divided into neural and non-neural ectoderm, but signaling events have already started to define finer subdivisions around the NP [18–22]. The second stage (St. 14) is an advanced NP stage, corresponding to mid-neurulation, before the neural folds begin to elevate. NP, NB, or NNE specification markers are already robustly expressed by this stage, albeit in partially overlapping patterns (e.g., *sox2*, *pax3*, and *epidermal keratin*, respectively; Fig 1B and not shown). According to a previous NP fate map [23], we have subdivided the NP into an anterior part (NPa), corresponding mostly to diencephalon and mesencephalon, and a posterior part (NPp), corresponding to rhombencephalon and spinal cord. The ANF, corresponding to telencephalon, was dissected together with PPE. Moreover, as the NB subdivisions are better characterized later on, when premigratory NC and placodes are segregated and fully specified [24,25], we also dissected the premigratory NC and the adjacent lateral preplacodal domain in late neurula St. 17, according to published protocols [26,27]. These additional dissected regions allowed us to link gene expression in progenitor cells from St. 14 early neurula to the transcriptome of specified St. 17 NC and placodes. To incorporate further temporal information into our dataset, we also included WEs collected during late gastrulation and throughout neurulation: St. 11–11.5, 12, 12.5, 13, 14–15, 16–17, and 18–19 (Fig 1C, S1 Table). More than 300 dissected samples were quality checked by quantitative PCR analysis (qPCR) using diagnostic markers for NP (*sox2*), NNE (*epidermal keratin*, *xk81a1*), NB (*pax3*), NC (*snai2*), and paraxial mesoderm (*myod1*) (S2 Table). Despite the utmost care in dissections, a few attached mesoderm cells occasionally contaminated the posterior ectoderm samples; samples with the lowest *myod1* expression were selected. After this quality check, whenever possible, adjacent ectoderm pieces, i.e., dissected out from a single embryo, were chosen (details available in Gene Expression Omnibus [GEO] data repository, GSE103240). Finally, we selected samples with a yield above 40 ng of total RNA. According to standards in the field, 3 biological replicates were collected per dissected region, at St. 12.5 and St. 14. Each replicate originated from different embryos and different clutches to buffer interindividual variations. For NB ectoderm regions, which are our main research focus of interest, we were able to add a few more replicates, which would increase statistical power of the analyses in this region (S1 Table). For premigratory NC and PPE from the late neurula, between 2 and 4 biological replicates were dissected at St. 17. A total of 79 samples were finally used for polyadenylated RNA selection and cDNA library preparation. Each was then subjected to a classical pipeline of downstream data analysis using paired-end sequencing on a HiSeq 2000 (Illumina) (Fig 1D; S1 Table; see details in Materials and methods).

The RNA sequencing from polyadenylated transcripts yielded at least 14 million reads per sample. About 88% of the reads mapped to unique loci in the *X*. *laevis* genome assembly (v9.1) [16]. In addition, we predicted novel transcripts, which were included in Gene Model Annotation 1.8.3 of the *X*. *laevis* 9.1 Genome Build (http://www.xenbase.org/entry/doNewsRead.do?id=223). In the following sections, we focus on spatial and temporal analyses at early and mid-neurula stages. Then, we describe how we use the whole dataset (tissue dissections and time series) to build a gene co-expression network. Finally, we present EctoMap, a user-friendly interactive tool that aims to provide insight on the study of temporal and spatial relationships of genes using *X*. *laevis* neurula as a prototypical vertebrate model.

### Principal component analysis decoded fine positional information from the transcriptome

The first step of analysis was to visually evaluate samples grouping. We compared 2 commonly used dimension reduction techniques: principal component analysis (PCA), a linear multivariate technique, and t-distributed stochastic neighbor embedding (tSNE), a stochastic and nonlinear approach.

A total of 31,597 transcribed loci were found in the dissected samples, out of 45,099 reported in the *X*. *laevis* genome [16]. In order to improve the analysis, we have filtered out the genes with little variation in expression (S1A and S1B Fig). Based on silhouette analysis, which also indicates the number of genes to keep, we defined a list of 2,198 genes with high range of expression (1,174 at St. 12.5 and 1,859 at St. 14, see Materials and methods). This list of genes was used in the subsequent unsupervised approaches (PCA, tSNE, and in expression pattern predictions calculated using a nonnegative matrix factorization [NMF] algorithm, see below). All the transcripts excluded from the initial analyses were reintroduced later in the study, including in NMF analysis, to document their spatial distribution and time course.

PCA and tSNE capture the major differences in gene expression between samples, with the aim to describe a complex dataset (consisting of a large number of samples, each defined by thousands of gene expression values) and represent this dataset in a space with fewer dimensions. While these algorithms are often used merely to check that replicate samples group together and that experimental conditions (here, dissected regions) are well separated, the mathematical definition of PCA potentially allows exploring further the structure of the dataset. In the case of PCA, dimensions are defined to capture the highest amount of variation (percentage of the variance) from the high-dimensional gene expression space. By identifying the biological meaning of each dimension (called "component"), one could get insight into the most important factors influencing gene expression in the dataset.

**Fig 2:**
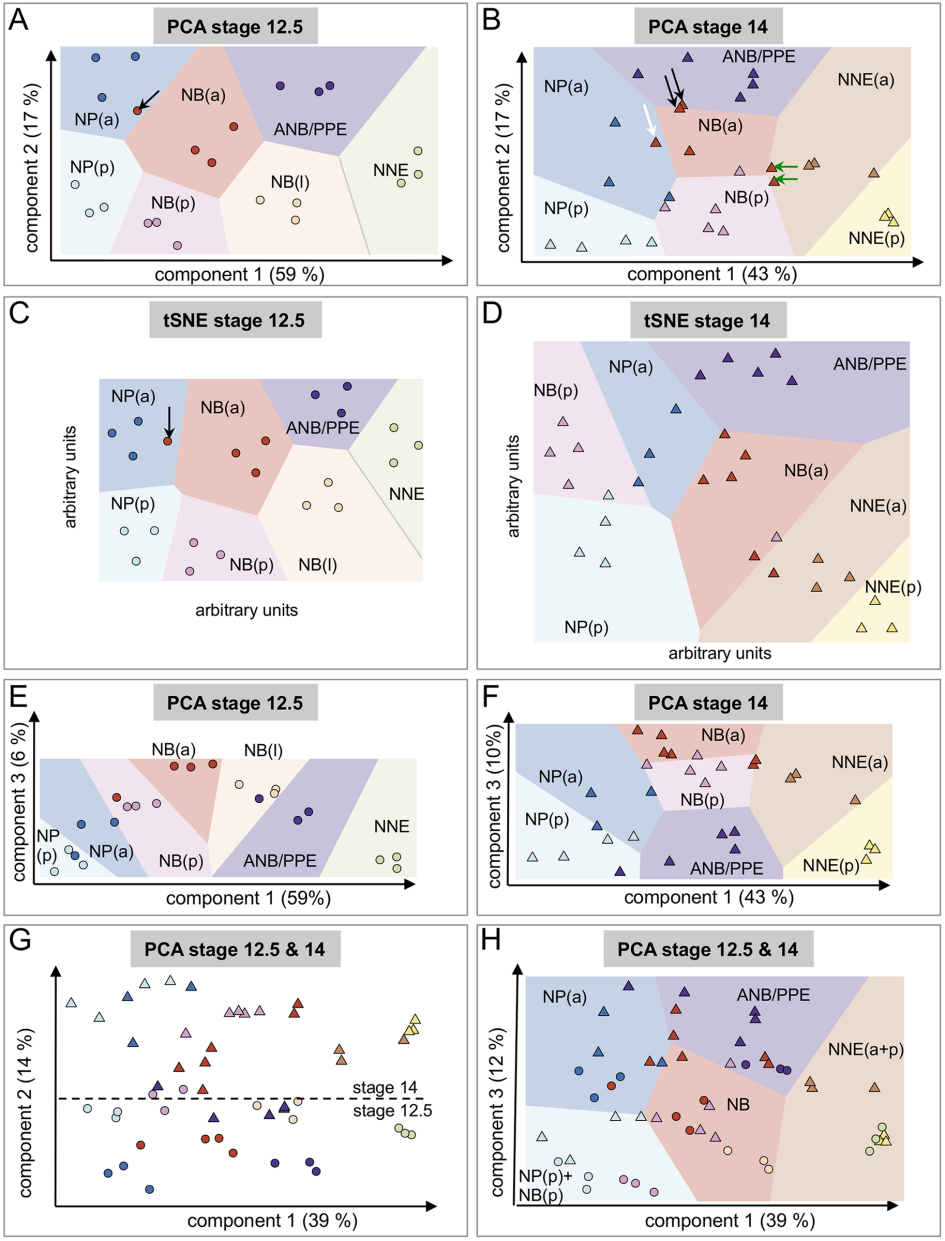
Unsupervised analysis of variance between samples using principal component analysis (PCA) retrieves biologically meaningful components, matching embryo polarity axes, neural border (NB) formation, and developmental stage. (A, B) At Nieuwkoop and Faber stage (St.) 12.5 (A, circles) and St. 14 (B, triangles), the first 2 PCA components segregated ectodermal samples according to their position along the dorsal-ventral (D-V) and anterior-posterior (A-P) embryonic axes, respectively. Percent of total gene expression variance captured by each component is indicated between parentheses. Sample color code is identical to Fig 1A. Lighter background colors are Voronoi diagram areas. These areas are drawn using the barycenter of samples belonging to the same dissected region. Each area indicates the regions of the PCA plane, where all points are closer to the barycenter of this dissected region than to any other barycenter. Arrows indicate anterior neural border samples falling slightly outside their intended dissected region (see text for details). (C, D) At St. 12.5 (C) and St. 14 (D), t-distributed stochastic neighbor embedding (tSNE) groups samples according to dissected regions. (E, F) At St. 12.5 (E) and St. 14 (F), neural border samples are located higher than most others along the third PCA component. This indicates that component 3 correlates with the acquisition of the NB characteristics during neurulation. (G, H) When combining St. 12.5 (circles) and St. 14 (triangles) samples together, the first component remains correlated to the D-V axis. The second component sorts out most samples according to their stage, irrespective of their position along the D-V axis (triangles separated from circles, (G). The first and third PCA components segregate most samples according to their position along the D-V and A-P axes, irrespective of their stage (circles and triangles of same color together, [H], Voronoi diagram). The posterior neural border (NBp), which is known to resemble the posterior neural plate (NPp) at St. 12.5, grouped with the NPp, while the anterior neural border (NBa)–St. 12.5, NBa–St. 14, and the lateral neural border (NBl)–St. 12.5 belong to the same Voronoi cell. As noticed above, NBa–St. 14 is more heterogeneous (red triangles).

We first analyzed each stage separately, using data from genes selected at the corresponding stage (Fig 2). For PCA, samples were plotted along the first 2 components and in 2 dimensions for tSNE (Fig 2A, 2B, 2C and 2D). With either method, we observed a good separation of most samples according to the dissected regions and adequate grouping of the samples representing replicates for the same dissected region (Fig 2). To further visualize the outcome of the PCA/tSNE plots, we used Voronoi diagrams. These diagrams partition the plane of PCA using the barycenter of replicates for a given dissected region; each area contains all points closer to this barycenter than to barycenters of other regions (Fig 2A–2D). These diagrams are meant to show whether replicate samples for the same dissected region lay closer to each other than to other dissected regions. In particular, this could be used to identify outlier samples. At St. 14, tSNE separates, better than PCA, the ANF/PPE from the NBa. This difference between methods could reflect the complexity of the edges of the neural plate, which have been separated into different subregions here (medially located ANF versus laterally located NBa); these adjacent ectoderm regions, which are highly dynamic and rather small, could share an important part of their transcriptomes and were likely to be dissected out with some variation from embryo to embryo. In this case, tSNE would be preferable to PCA. From simple usage of unsupervised dimension reduction algorithms, the first conclusion was that the replicate samples grouped as expected using thousands of genes with high variation in expression. This meant that for each neurula stage, ectoderm dissected regions could be recognized based on a large part of their transcriptome.

Moreover, exploiting PCA mathematical properties, we could retrieve interesting biological information from the structure of the dataset, as explained below. This deeper analysis was possible in the case of our experimental design, because a few PCA dimensions (named components) captured a large amount of the variance between samples (S2 Fig). At both stages, St. 12.5 and St. 14, the first PCA components, which capture the highest percentage of variance, were identified as significant at the 1% level: components 1 to 3 at St. 12.5 and components 1 to 4 at St. 14, respectively (S2 Fig). Moreover, the first 2 components retain a very significant amount of total variation both at St. 12.5 and St. 14, respectively, 76% and 60% (S2 Fig). When samples were plotted along these first 2 dimensions, at both stages, the first component separated dissected regions according to their position along the embryonic D-V axis, and the second component separated dissected regions according to their position along the A-P axis of the embryo (Fig 2A and 2B). A similar distribution of dissected regions along the first PCA components was obtained when, instead of computing PCA with all samples, it was computed from average gene expression values for each dissected region (not shown). Together, these results indicated that transcriptome-scale differences between 2 given ectoderm regions, either at St. 12.5 or at St. 14, were primarily and significantly correlated to the position of the dissected region along the D-V axis first, and secondly along the A-P axis. In biological terms, this means that more than 60% of variation in gene expression in the ectoderm during early and mid-neurulation is related to positional information along these 2 axes.

Experimental analyses have previously identified genes whose expression strongly correlates to embryonic axes. For example, transcription factors such as regional neural markers are clearly differentially expressed along D-V or A-P axes (*otx2*, *gbx2*, *cdx*[28,29]). Here, using PCA, we could identify which genes best correlate with PCA components 1 and 2. Because PCA component 1 matches with embryonic D-V axis and PCA component 2 with A-P axis, we propose that by knowing the expression level of the genes most correlated with each axis, we could approximate the spatial position of unknown ectoderm sample along these axes. For each axis, expression level of selected genes would provide D-V and A-P coordinates (positional information) in the plane of ectoderm.

To test and illustrate this hypothesis, we identified which genes significantly contribute to components 1 and 2 (*p*-value < 0.05) at both embryonic stages. We provided the list of the 60 most significant ones for each axis at each stage of development (S4 Table). These were also the genes most correlated with the 2 main PCA components, at St. 12.5 and St. 14 (S3 Fig). As an independent confirmation of the assignment of the PCA components, we reconstructed the spatial expression of the 5 genes most correlated to components 1 and 2 at St. 12.5 and St. 14 (S4 Fig, see NMF prediction below). Indeed, the genes most negatively correlated to component 2 included *caudal* family genes *cdx2* and *cdx4*, and *hox* genes, all expressed in posterior structures in vivo, while genes correlated positively to component 2 were brain-specific genes *otx1*, *otx2*, *fezf2*, or *crx* [30–34]. Most correlated genes maintained a pattern restricted along D-V or A-P axes at mid-neurula stage (S3 Fig, S4 Table). We concluded that the expression level of the genes most correlated to PCA components 1 and 2 are diagnostic markers for cell position along D-V and A-P embryonic axes during neurulation.

According to this hypothesis, if PCA faithfully described D-V and A-P position in the plane of ectoderm, it should also uncover subtle spatial heterogeneities between the biological replicates for a given dissected region (Fig 2). Hence, for a given sample (known or unknown), comparing the expression level of the diagnostic genes for each axis would indicate a sample's position along each axis. For example, at St. 12.5 (Fig 2A), the 3 replicates for each ectoderm area fall into the defined zones homogeneously, as all 3 dots, 1 for each replicate, are included in the domain defining a given sample type, usually close to one another. One exception is a fourth replicate of the NBa (dark red, black arrow), which shares more global gene expression similarity with the adjacent NPa (navy blue), suggesting that this sample may have been dissected slightly more medially than the other St. 12.5 NBas. For this sample, we tested expression levels of the 2 genes most correlated to the D-V axis to test this hypothesis. Indeed, *zc4h2*.*l* and *rfx4*.*l* expression were higher in this sample than in the other NBa replicates (S3 Fig).

As another example, at St. 14 (Fig 2B), biological replicates for the neural plate (NP(a) and NP(p), light and navy blue), ANF/PPE (dark blue), and the lateral NNE (a and p), (brown and yellow) are rather homogeneous. However, as the dorsal ectoderm is patterned into smaller subdomains with few morphological landmarks to guide the dissection, the heterogeneity between replicates is higher in the transition zone formed by the lateral NB, mainly the anterior part (NB(a), dark red). As expected for an accurate dissection, all NBa replicates display high *snail2* and *pax3* expression, a known characteristic of this area. However, additionally, we find that 2 replicates shared a partial signature with the ANF/PPE (black arrows), 1 with the NP(a) (white arrow), and 2 with the NNE(a) (green arrows). In terms of landmark gene expression, the 2 samples closer to the preplacode domain express the lowest level of the posterior gene *cdx4* expression of the 5 replicates, thus marking them as more anterior along the A-P axis (PCA axis 2). Similarly, the 2 replicates close to the lateral ectoderm samples (green arrows) express the lowest *sox2* levels of the 5 replicates, thus suggesting they were dissected slightly farther from the dorsal midline of the embryo. This suggests that the samples all cover the intended area, with small variations around a “typical” or average transcriptome established by PCA. This also means that the PCA, computed for the genes with highest expression range, accurately reconstitutes individual replicate position around a median point defined for each sample type, without prior information on a dissected region gene signature. Such refined analysis may be important for including or excluding individual replicates or for assigning a spatial identity to an unknown sample, even a single cell, in future analyses (e.g., assessing modifications of positional information after mutation of a given gene or identifying differences in position for a cluster of single cells). Here, as we performed a global analysis of ectoderm patterning, we were interested in depicting all samples, including variability in their positions, and we have chosen to keep all samples in the analysis.

### PCA identified gene expression variations due to ectoderm developmental timing and NB formation

Statistical analysis of PCA component distribution showed that a third dimension was also informative (S2 Fig): when plotting samples along the first and third PCA dimension, the NB samples were positioned higher than the others along PCA component 3. This third dimension behaved similarly at St. 12.5 and St. 14 and captured 6% and 10% of gene expression variance, respectively (Fig 2E and 2F). Genes most correlated with this third dimension included known NB/NC specifiers such as *snail1*, *pax3*, *sox9*, *msx2*, and *lmx1b*. This meant that a small but measurable amount of gene expression variation was linked to NB differentiation and that PCA captured the emergence of the NB as a significant contributor to region-specific transcriptome variations during early neurulation, in addition to variation along the D-V axis already captured by the first PCA dimension.

Next, to test if dissected regions at St. 12.5 and St. 14 shared similar variations in gene expression, we analyzed together the 65 samples using PCA (Fig 2G and 2H). In this larger analysis with both neurula stages together, the first PCA dimension still positioned the samples along the D-V axis, while the third (and not the second as previously) dimension referred to the A-P axis (Fig 2H). Interestingly, similar dissected regions from both stages occupied similar locations along component 1; this suggested that D-V positional information of a given ectoderm region, as encoded in the transcriptome, was relatively stable during early and mid-neurulation (Fig 2G). However, the temporal dynamics of gene expression was important, as the second dimension encoded the temporal differences because it segregated most samples from St. 12.5 (circles) and St. 14 (triangles), irrespective of their dissected region identity (Fig 2G). Further functional studies are needed to identify the main parameters supporting this variation between early and mid-neurula samples, as a biological function for the genes most correlated to component 2 remained elusive. Finally, the fourth dimension separated the NB region from the other ectoderm areas, with high correlation of genes expressed in the NB and NB to this component, as seen in the stage-specific analysis (*egr4*, *msx1*, *msx2*, *pfkfb4*, and *sox9*) [6,35–37].

In general conclusion on this part of the analysis, PCA and related approaches allowed visualizing the most prominent differences between samples and dissected regions for the various experimental conditions. We confirmed the accuracy of dissections using thousands of genes with the highest expression range and observed that most samples tend to group according to the 7 dissected regions at each stage. Compared to landmark gene analysis (e.g., a few marker genes analyzed by qPCR), PCA was more detailed and retrieved the main factors (components) that accounted for variation between samples. These components significantly correlated with positional information (D-V and A-P axes), NB formation, and developmental time progression. PCA also revealed small variations between biological replicates that could complicate the establishment of a unique and robust gene signature for each dissected region. We thus turned to a deconvolution approach to overcome this last point.

### Coarse-grained quantitative ectoderm-specific gene expression patterns were reconstructed by nonnegative matrix factorization (NMF) deconvolution

In order to complement the PCA, which highlights variation in gene expression between samples, we next focused on identifying robust spatial tissue signatures from our samples by looking for a common gene signature for biological replicates of a given dissected region, irrespective of technical/biological variation between replicates. However, statistical tests of differential expression applied to adjacent dissected regions did not recover any dissected-region specific genes signature. This was explained as we observed that markers highly specific for a tissue could be found at lower levels of expression in some samples from adjacent tissues, potentially indicative of slight shifts in dissection position; *snai2*, a specific marker of the lateral NB at St. 14, was found expressed at lower levels in 3 out of 8 biological replicates of the adjacent NPa and anterior ectoderm, suggesting that these 3 samples contained a few NB cells (Fig 1, S5A Fig). At the same time, in such early embryos, most genes are not tissue specific; *sox2* marks NP with high expression levels but is also expressed in the adjacent NB and in the lateral PPE at lower levels (S5B Fig). We were therefore looking for an algorithm that could identify and discard small contributions from adjacent tissues, due to biological or technical variability, while correctly identifying contribution of the main dissected tissue. This is commonly achieved using such deconvolution approaches as the NMF algorithm [38,39].

**Fig 3:**
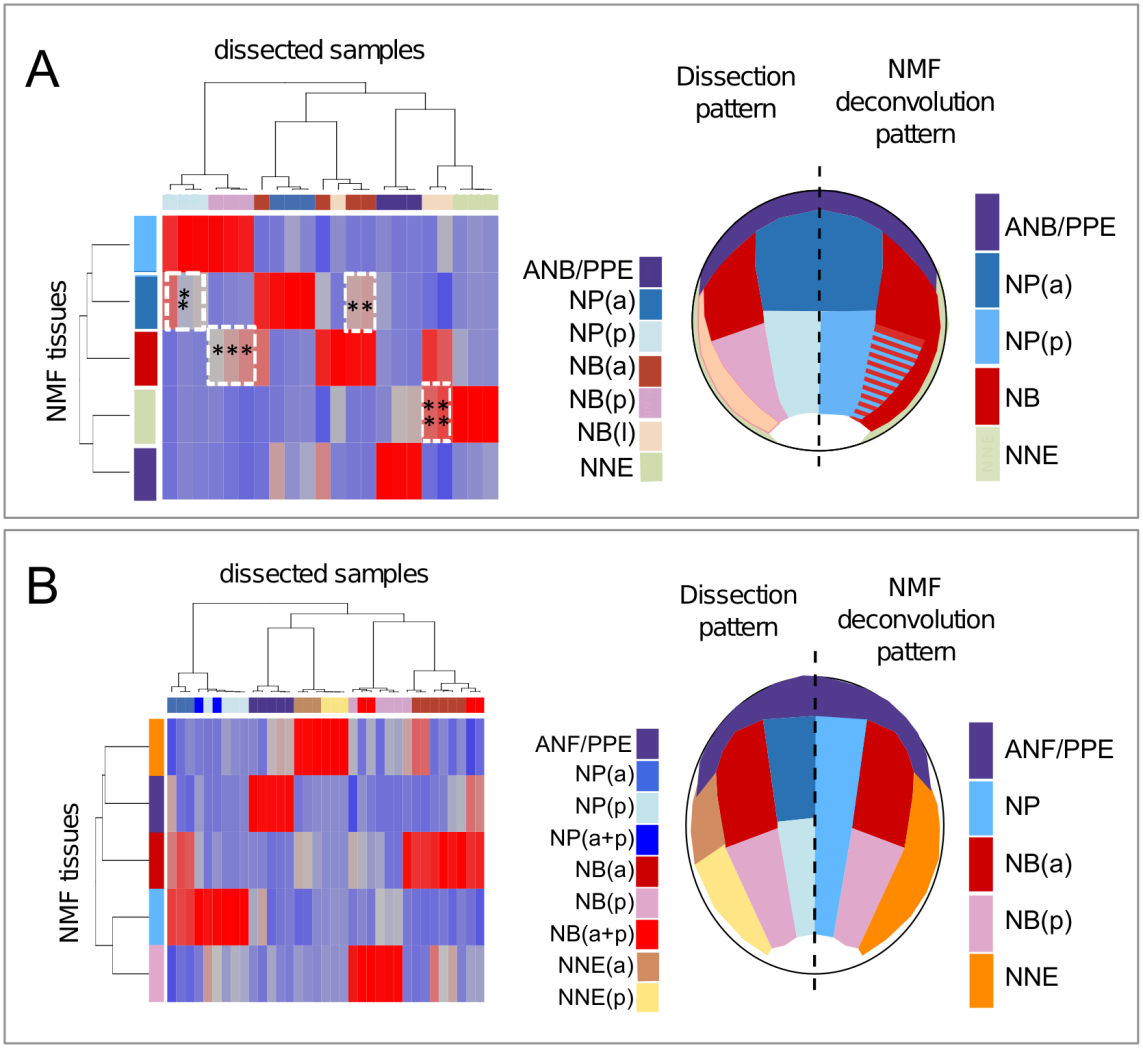
Nonnegative matrix factorization (NMF) efficiently deconvolutes differential expression profiles. The NMF algorithm defines a set of ectoderm tissues (NMF-tissues) at Nieuwkoop and Faber stage (St.) 12.5 (A) and St. 14 (B). Columns in the mixing matrix indicate how these tissues contribute to the initial dissected samples. Red indicates high contribution; blue indicates low/no contribution. Interesting intermediate contributions are outlined by white dotted lines; stars (*) refer to comments in text. From these contributions, dissected samples (left part of the pattern drawn on embryo) were matched with 1 of the NMF-tissues (right part of the pattern). At St. 12.5, striped pattern indicated the mixed contribution of posterior neural plate and neural border (NMF-NP(p), NMF-NB(p)) to NBp samples. Abbreviations and colors are the same as in Fig 1 and S1 Table.

In this model, each dissected region is "a priori" proposed to be a mix of a few "prototypical" ectoderm regions, the transcriptome and proportions of which must be estimated in each dissected region. For example, one could hypothesize that the NB transcriptome could either be unique compared to the other ectoderm regions or could result from a combination of NP transcriptome and NNE transcriptome. The algorithm of choice should identify how many prototypical transcriptomes could be defined from the dataset and reconstruct how the actual transcriptome of each dissected region corresponds to a combination of the prototypical transcriptomes. The NMF algorithm reconstructs a sample expression matrix (our complex transcriptome dataset) as a product of 2 matrices: the prototypical tissue expression matrix, which describes gene expression in the reconstructed tissues (named below as "NMF-tissue" or "NMF-predicted"), and the mixing matrix, which describes how NMF reconstructs dissected regions from NMF-tissue with the constraint that the transcriptome expression and the proportions matrices are nonnegative. When running the algorithm, one looks for the number of prototypical tissues that produce a robust solution (convergence on a single solution from multiple random initializations, see Materials and methods). When applied to our dataset, the NMF algorithm robustly recovered 5 prototypical NMF-tissues at each neurula stage (Fig 3, S6 Fig). Based on the contribution of NMF-tissues to each dissected sample (represented in shades of blue [low] to red [high]), these NMF-predicted tissues could be straightforwardly assigned to the 7 dissected tissues (Fig 3A and 3B). The hypothesis of 6 or 7 prototypical tissues did not produce stable results, indicating that the NMF algorithm could not find a robust solution (S6 Fig).

The hypothesis of 5 prototypical NMF-tissues results in the mixing matrix shown in Fig 3A and 3B. The embryo map shows how these 5 prototypical tissues relate to our 7 dissected ectoderm regions (Fig 3A and 3B). First, the main contributions were considered in the mixing matrix (indicated in red). At St. 12.5, 3 of the 5 domains identified by NMF matched directly with the dissected region pattern: the ANB/PPE (violet), the NPa (navy blue), and the ventral NNE (green) (Fig 3A). These 3 biological tissues thus showed a clear-cut and specific signature by NMF deconvolution as also reflected by their clear segregation along the first 2 PCA dimensions. In contrast, the NMF algorithm merged the dissected NPp (light blue) and NBp (pink), indicating that the transcriptomes of these 2 tissues are very similar, although they could clearly be separated along the first 2 PCA dimensions. They were grouped into a single NMF-tissue named "posterior neural plate" (NPp, solid blue and hatched blue-red) (Fig 3A). Similarly, anterior (red) and lateral (beige) NB were merged into a single NMF-predicted tissue named "neural border" (NB, red). The comparison between the dissection pattern and the deconvoluted NMF profile was schematized in Fig 3A. Besides these main components (in bright red), when secondary contributions were considered (indicated in shades of light blue to pink in the mixing matrix, Fig 3A), St. 12.5 analysis by NMF also identified that the NMF-predicted NB tissue (red) displays a minor contribution to the dissected NBp (pink, in dotted square, ***) and that the NMF-predicted NPa domain (navy blue) contributed to adjacent dissected regions, the NPp (light blue) and the NBa (red) (in dotted squares * and **). In a similar manner, the NMF-defined ventral ectoderm partly contributed to the lateral NB component (beige, ****). In conclusion, when similarities were considered (and not differences, as for PCA), unsupervised deconvolution by NMF distinguished 5 main tissues in the late gastrula ectoderm, giving rise to the simplified pattern depicted in Fig 3A. Moreover, NMF analysis was also able to identify minor contributions of NMF-tissues to samples from adjacent dissected regions. In contrast, NMF-tissues did not contribute to more distant dissected regions (meaning that there was no cross contribution between NNE and NP). This indicated that NMF-tissues indeed corresponded to spatially restricted tissue types.

**Fig 4:**
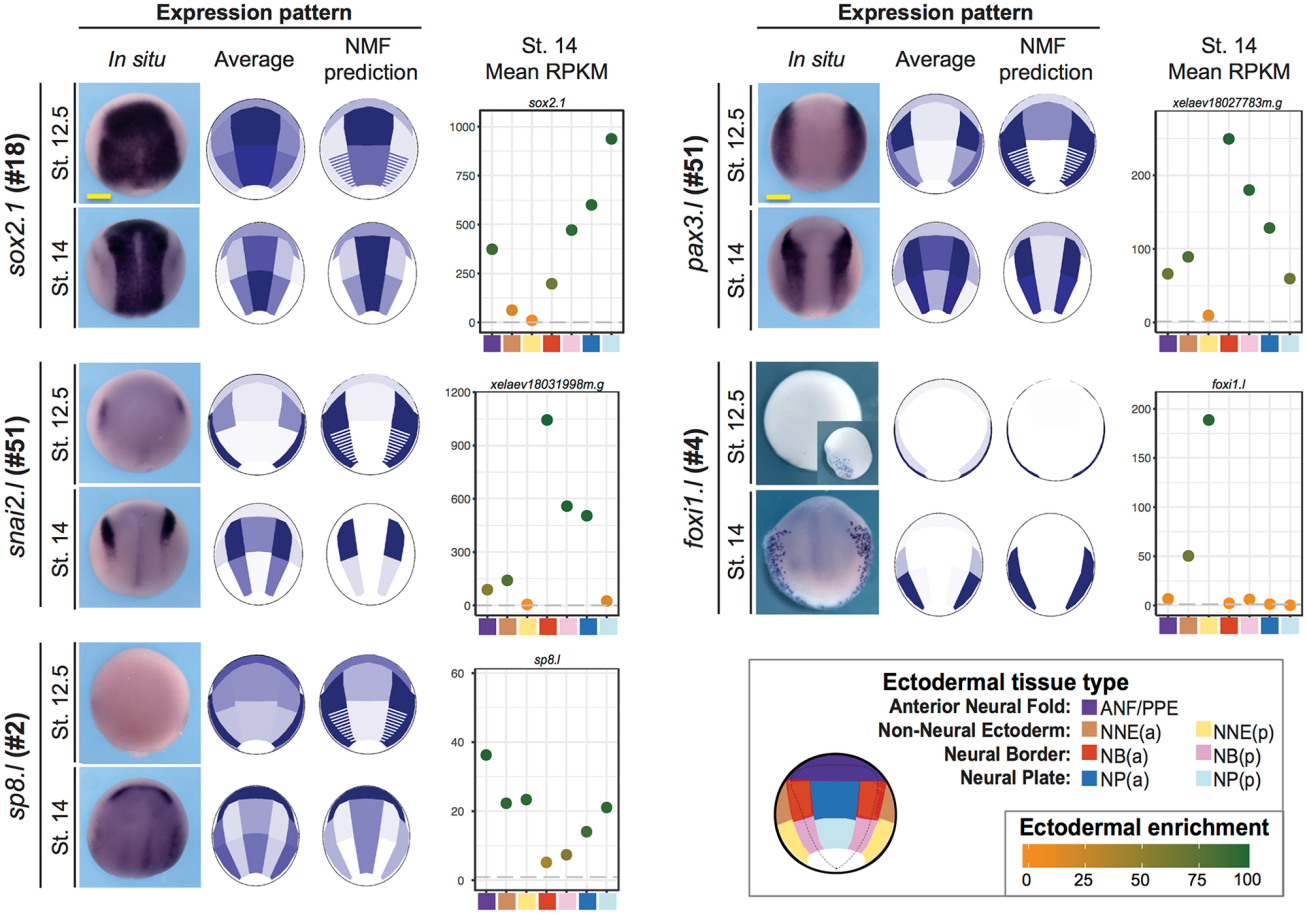
Nonnegative matrix factorization (NMF) predicts accurate expression patterns for neural border (NB) genes. In order to represent gene expression levels on the map of the developing ectoderm, we used 2 approaches: averaging the expression values for each of 7 domains or using NMF deconvolution predictions in 5 domains. We found those 2 approaches complementary. Average patterns directly describe raw data but are sensitive to shifts in dissection. NMF deconvolution predicted expression levels in each NMF-tissue. In the case of the anterior neural border (NBa), the NMF pattern is closer to in situ hybridization patterns than average patterns, e.g., for known genes expressed in various ectoderm areas, *sox2*, *snail2*, *pax3*, and *foxi1e* (also see Fig 8, S4 and S7 Figs for more examples). Moreover, these patterns predict expression for genes of unknown pattern and with low expression level, such as *sp8*. We present the average pattern and NMF-predicted pattern (NMF pattern); each is normalized on a percent scale, 100% being maximal expression of the gene of interest. This implies that genes with low expression will be represented in similar shades as genes expressed at high level, which allows better visualization but could be misleading if the gene is very weakly expressed in all ectoderm (e.g., *sp8* at Nieuwkoop and Faber stage [St.] 12.5). To complete pattern prediction with expression level information, we thus also include the average expression levels per dissected sample for each gene (reads per kilobase per million [RPKM] shown for St. 14 here). Robustly expressed genes (>100 RPKM, e.g., *sox2*, *snail2*, and *pax3* at St. 14) are predicted as well as genes with very low expression (e.g., *snail2* at St. 12.5 or *sp8* at St. 14). In addition, the color of the dot indicates high ectoderm specificity (green) to low ectoderm enrichment (orange). See text and Materials and methods for details, see S11 Table for numerical data.

At St. 14, using a similar interpretation, the algorithm also identified 5 tissues with a different grouping of dissected tissues as compared to St. 12.5. The ANF/PPE (violet), the anterior lateral NB (red), and the posterior lateral NB (pink) were defined as anticipated by dissections. In contrast, the anterior and posterior NP (blue) as well as anterior and posterior NNE (brown and yellow) were merged (Fig 3B). Thus, by using NMF analysis, we retrieved information on spatial gene pattern within 5 expression domains, allowing us to define tissue-specific gene signatures on the genome scale, albeit with less spatial sensitivity than PCA (which recognized variations between 7 domains). When compared to the results of plotting averaged gene expression (made from averaging biological replicate expression levels, as more usual), NMF-defined spatial gene expression was useful to detect the areas of high expression, when dissection shifts may have occurred between adjacent regions or when genes may be expressed with high variability between 2 adjacent regions (S3 Fig), compared to somewhat noisier averaged expression. In particular, this improved inference of gene expression in the NBa at St. 14 (see, for example, *snai2* or *sox10* expression at St. 14, with low expression in the NPa on average pattern, while NMF-predicted pattern matches better with in situ hybridization pattern, Fig 4, S8 Fig).

Above all, NMF was advantageous because it retrieved semiquantitative information on sample identity on the genome scale, even when a statistical approach for defining differential expression could not distinguish between adjacent areas. As mentioned above, in addition to the identification of each sample with a major contributing dissected tissue (in red), NMF deconvolution highlighted minor contributions to the transcriptome signatures of adjacent tissue samples (shades of pink and light blue, Fig 3A and 3B). Using this mixing matrix, which is based on the subset of genes with high variation in expression, we could thus extrapolate the NMF information to generate a semiquantitative, coarse-grained spatial map of gene expression for all of the 31,597 transcripts expressed in our samples. We then validated that the NMF algorithm was able to accurately predict the expression pattern of specific *X*. *laevis* transcripts in the ectoderm, post hoc, by comparing the NMF-predicted expression patterns with in situ hybridization patterns for known genes (*pax3*, *sox8*, and *foxi1*), or predictively, after cloning and examining novel genes of previously unknown pattern (*sp8* and *sh3pxd2a*) (Fig 4, S4 and S7 Figs).

Thus, using NMF, we could present sensitive and semiquantitative coarse-grained (5 regions at each stage) expression predictions for each of the 31,597 transcripts expressed in the developing ectoderm of a frog embryo at the neurula stage (Fig 4). When compared to in situ hybridization patterns in vivo, the predicted NMF patterns closely resembled the in vivo patterns and were sharper compared to the average patterns. As seen for genes expressed at low/very low level, such as *snai2*, *sh3pxd2a*, and *sp8* at St. 12.5, the predicted patterns were rescaled to enhance them: color intensity in each domain is a percent of highest expression (compare the rendering for low *sp8* expression in whole ectoderm to the difference with *foxi1e*, highly expressed ventrally but absent dorsally).

In order to provide an accurate description for each gene expression pattern, we complemented the expression pattern prediction with 2 additional levels of information: the averaged level of expression in each dissected region (in mean reads per kilobase per million [RPKM], matching the average pattern in a quantitative manner) and an index of gene expression enrichment in the ectoderm dissected region compared to WE at the same stage (Fig 4). We obtained this "ectoderm enrichment" indication by dividing the average expression level of a gene in each dissected region by the expression level in the WE (Fig 4, S8 Fig). This was plotted for each transcript with the normalized and averaged expression level on the y-axis and the dissected region on the x-axis; the relative enrichment in the ectoderm is indicated as a color gradient. Genes expressed highly in the ectoderm compared to WE average expression appear in dark green, while genes expressed at similar levels appear in dark orange. This allowed identifying ectoderm-specific gene expression (e.g., *sox10*.*l* displays high ectoderm enrichment, S8 Fig) compared to genes expressed in other germ layers, as well, or genes found because of mesoderm contamination (especially in posterior dissected samples; e.g., *myod*.*l* and *myf5*.*l*, found in NPp samples, are displayed in dark orange, indicating very low ectoderm enrichment, S8 Fig) [40,41]. Finally, these patterns at early and mid-neurula stages were completed with a time course of WE expression level at 5 neurula stages (S8 Fig).

Finally, we used the Gini coefficient (an index classically used in Economics to measure income distribution [42]) to evaluate the tissue-specific enrichment in gene expression for each NMF-predicted region and retrieved a tissue-specific gene signature list defining each NMF-predicted domain (S5 Table, S7 Fig). This signature consisted of the genes with the highest Gini index (0.8 for our dataset) or close to the maximum (0.7–0.8 on a scale of 0 to 0.8), as listed in S5 Table. At early neurula stage, this is the first genomic annotation of specific ectoderm spatial domains. In sum, we have described a genome-scale optimized pattern prediction using NMF, accompanied by an ectodermal enrichment score. Using the Gini index, we have defined the first tissue-specific gene signature for each ectoderm region.

### Clustering of spatial and temporal expression data identified synexpression groups associated to important cell and developmental biology processes

**Fig 5:**
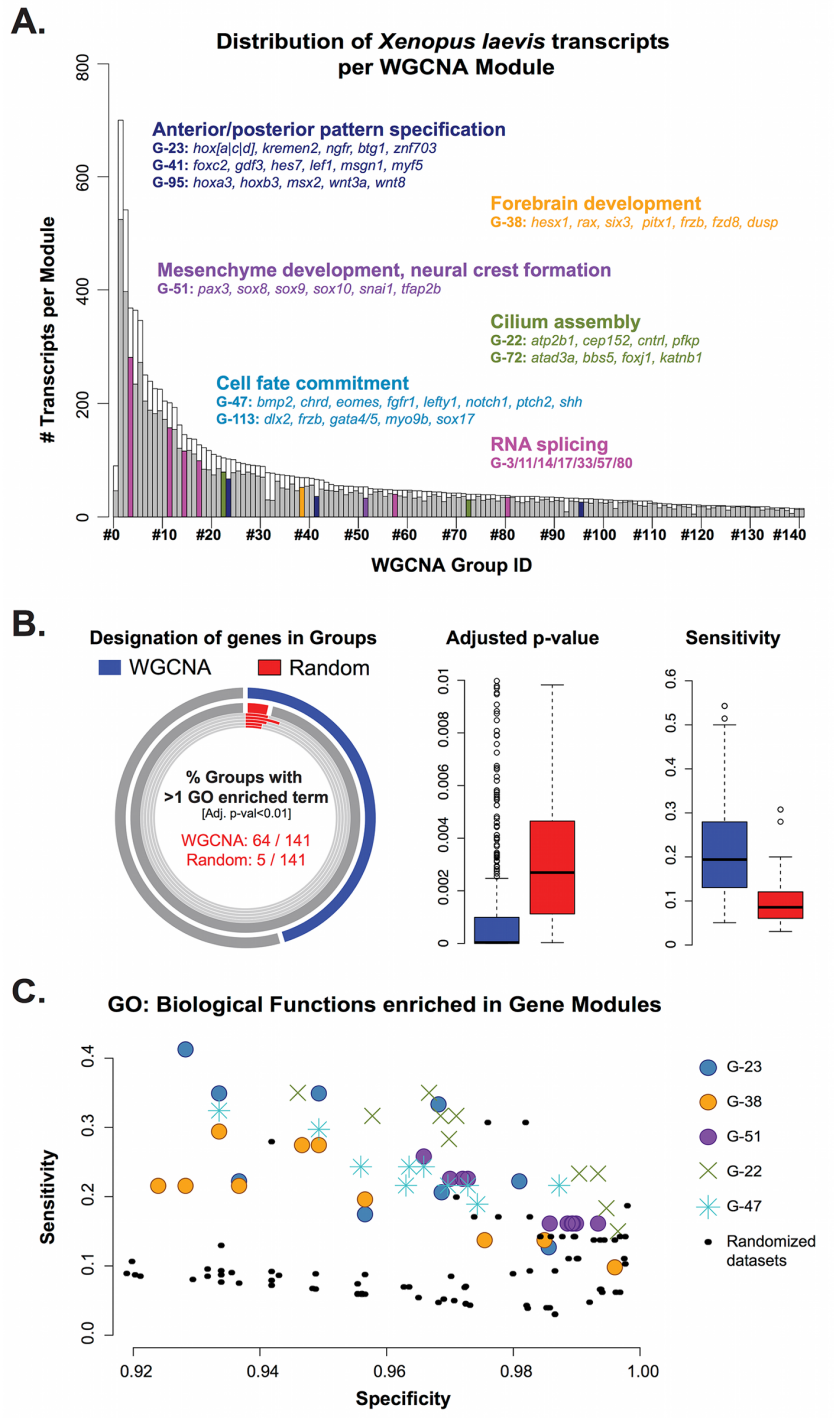
Identification of gene ontology (GO) biological functions enriched in weighted gene co-expression network analysis (WGCNA) groups. (A) Distribution of *Xenopus laevis* transcripts in WGCNA groups. The color-filled bars on the histogram indicate the number of unique Entrez geneID records per group. Functions of some developmentally relevant groups are highlighted: the Wnt responding genes in blue (Groups #23 and #41 in blue), neural crest (#51, purple), forebrain (#38, orange), mRNA splicing (#80, pink), and cilium morphogenesis in green (#22, green). Refer to S6 and S6B Table for the full list of functions associated with each group. (B) Comparison between groups generated by WGCNA (blue) and those created by random assignment of transcripts (red). About 45% of the WGCNA groups associate to at least 1 biological function, which is significantly more than the average obtained for the random dataset groups (4%; the 5 random datasets of 141 groups). The distribution of adjusted *p*-values and sensitivities (1-β) of overrepresented terms show that, overall, WGCNA groups are more likely to contain a relevant biological function than groups assigned randomly. (C) Specificity versus sensitivity plot, in which each of the data points represents a single GO function enriched in a group. Black circles represent all 80 biological functions enriched in the 5 random datasets (total of 705 groups). The Wnt responding genes (Groups #23, blue), neural crest (#51, purple), forebrain (#38, orange) are highlighted. For further description of the function, refer to S6B Table; see S11 Table for numerical data.

In eukaryotes, genes involved in related biological processes are often co-regulated and share similar regulatory inputs that coordinate their expression, temporally and spatially [43,44]. To gain insights into the temporal and spatial dynamics of the genes expressed at neurulation, we used weighted gene co-expression network analysis (WGCNA), a data-mining method widely used in biology to identify groups that share similar expression profiles from large gene expression datasets [45]. For this part of analysis, we used 10,073 transcripts, expressed above a background threshold level (see Materials and methods), using the whole dataset, i.e., 79 samples, including the dissected samples at 3 neurula stages (St. 12.5, St. 14, and St. 17) and the WE series (Fig 1C and S1 Table). These transcripts corresponded to a subset of 6,035 unique human orthologous genes, identified by their respective HUGO names. WGCNA generated 141 synexpression groups, which were ordered according to their number of transcripts (Fig 5; S9 Fig). Group #1 (referred as G-1) was the largest, with 653 transcripts, and G-140 the smallest, with 15 transcripts (15 being the minimal number required to form a cluster), with the exception of group #0 (G-0, *n* = 90, unclassified transcripts) (Fig 5A, S9 Fig).

In order to identify biological functions associated to each WGCNA generated gene module, we used an unsupervised functional enrichment test using gene ontology (GO). We found that 64/141 groups were significantly enriched for important biological functions (see Materials and methods, S6A and S6B Table). We next tested if this enrichment was significant over a randomized result. Overall, these overrepresented functions were more significant and more sensitive than random datasets (Fig 5B and 5C). We challenged the biological significance of WGCNA synexpression groups by exploring the distribution of 2 distinct sets of genes across the WGCNA modules: genes related to canonical Wnt signaling and genes involved in NC and placode GRNs.

**Fig 6:**
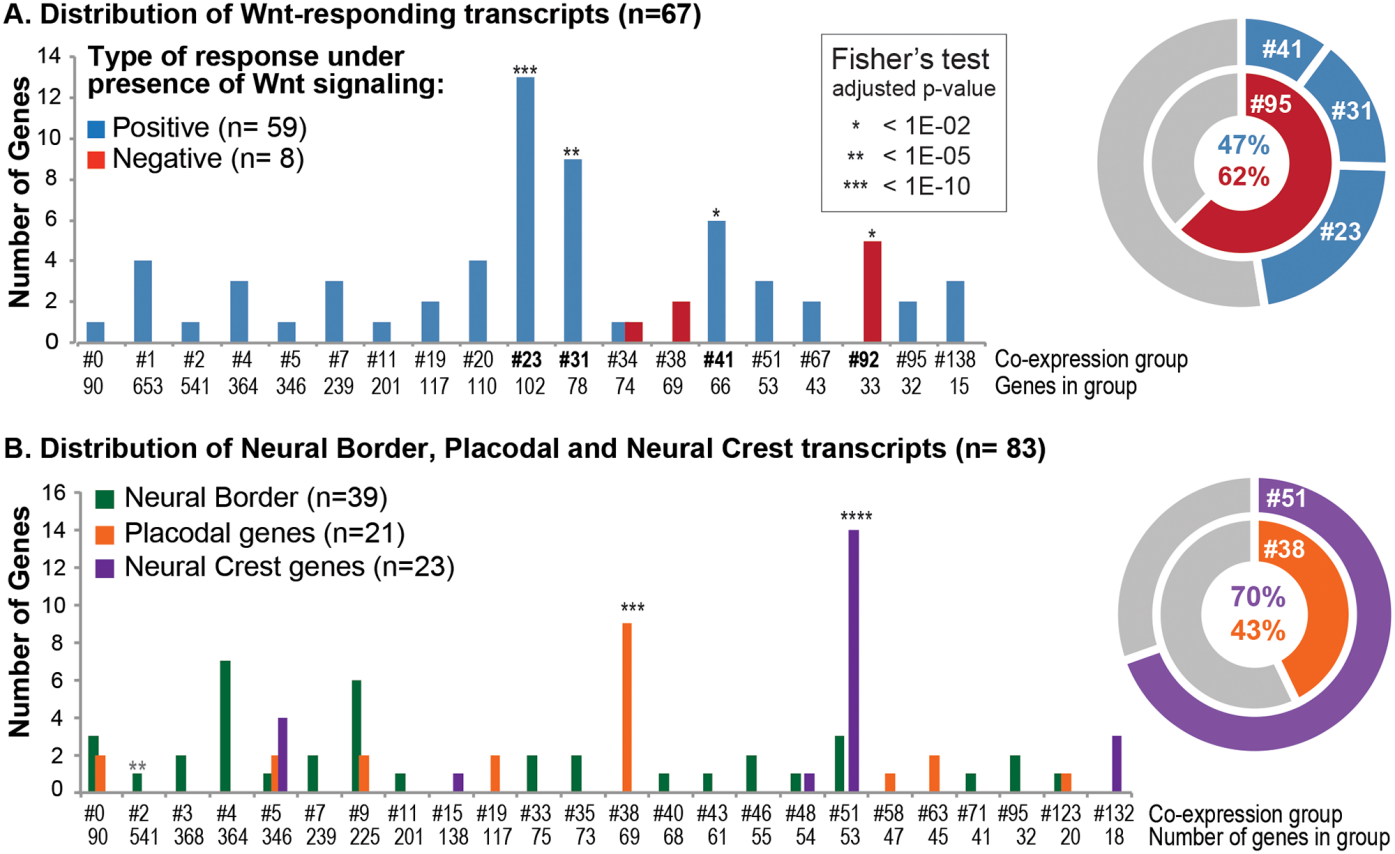
Distribution of Wnt-responding genes and genes involved in neural, neural crest (NC), and placodal development across weighted gene co-expression network analysis (WGCNA) synexpression groups. The histogram shows how many transcripts were found in each synexpression group and if this represented a significant enrichment compared to uniform distribution in all groups; see S11 Table for numerical data. (A) The Wnt-responsive transcripts identified by Kjolby and Harland [49] were mainly distributed in 4 co-expression groups: 47% of the genes positively regulated by Wnt signaling from the input list were found in groups #23, #31, and #41, while 62% of genes negatively regulated by Wnt were found in group #92. Group #23 contained posterior genes (e.g., *cdx1/2/4*, *hes5/9*, *ngfr*, *kremen2*); group #31, Wnt-signaling components (*xarp*, *tcf7*); group #41, meso-endoderm specifiers (e.g., *msgn1*, *myf5*, *t*, *vegt*); and group #92, genes up-regulated in absence of Wnt signaling. (B) Placodal and NC genes were enriched in groups #38 (43% of the input list) and #51 (70% of the input list), respectively. Contrarily to NC markers, the neural border genes, many of which are more broadly expressed, were distributed across many different co-expression groups; however, *pax3* and *myc* were part of group #51.

Wnt signaling is essential in embryonic development and cancer [46,47]. In development, canonical Wnt signaling acts during gastrulation and neurulation to induce posterior ectoderm structures and NC [48]. To examine how WGCNA would cluster genes implicated in Wnt signaling, we retrieved a list of 97 *X*. *laevis* transcripts responsive to Wnt signaling inhibition in gastrulas (S7B Table) [49]. From this initial set, 67 transcripts (70%) were expressed in our neurula-stage dataset above the minimal threshold. We found that half of these Wnt-responding transcripts (33/67) were significantly overrepresented in 4/141 groups: G-23, G-31, G-41, and G-92 (Fig 6A, S7 and S7A Table, see Materials and methods for statistical tests). In particular, G-23 contained 62% of genes previously associated with Wnt signaling in cell cycle, cancer, or patterning including many transcription factors expressed posteriorly and known to directly respond to canonical Wnt signaling and participate in posterior embryo patterning, such as *meis3*, *cdx2/4*, *hes5*, *hoxa1*, and *znf703* (S7A-B [49,50]). These observations and the GO functions associated to this group (S6A and S7 Tables) suggest that G-23 gathered known and novel genes responding positively to canonical Wnt signaling and involved in posterior patterning or skeletal morphogenesis. G-31 contained genes expressed in the endoderm and mesoderm, which directly and positively respond to Wnt signaling (*msgn1*, *myf5*, *t*, *vegT*) [49]. G-41 did not associate to a particular GO term but contained genes encoding putative components of Wnt signaling pathway (*fdz10*, *tcf7*, *xarp*). Finally, the transcripts negatively regulated by Wnt signaling were significantly enriched in G-92 (no GO association) (Fig 6A). This first example showed that WGCNA synexpression grouping is biologically meaningful: it groups together, based on their spatial and temporal synexpression, genes with related biological functions. This could prompt further research on the novel genes clustered in G-23 with known Wnt-responsive genes, to test their response to Wnt and their function in development or other cell contexts (S7 Table).

**Fig 7:**
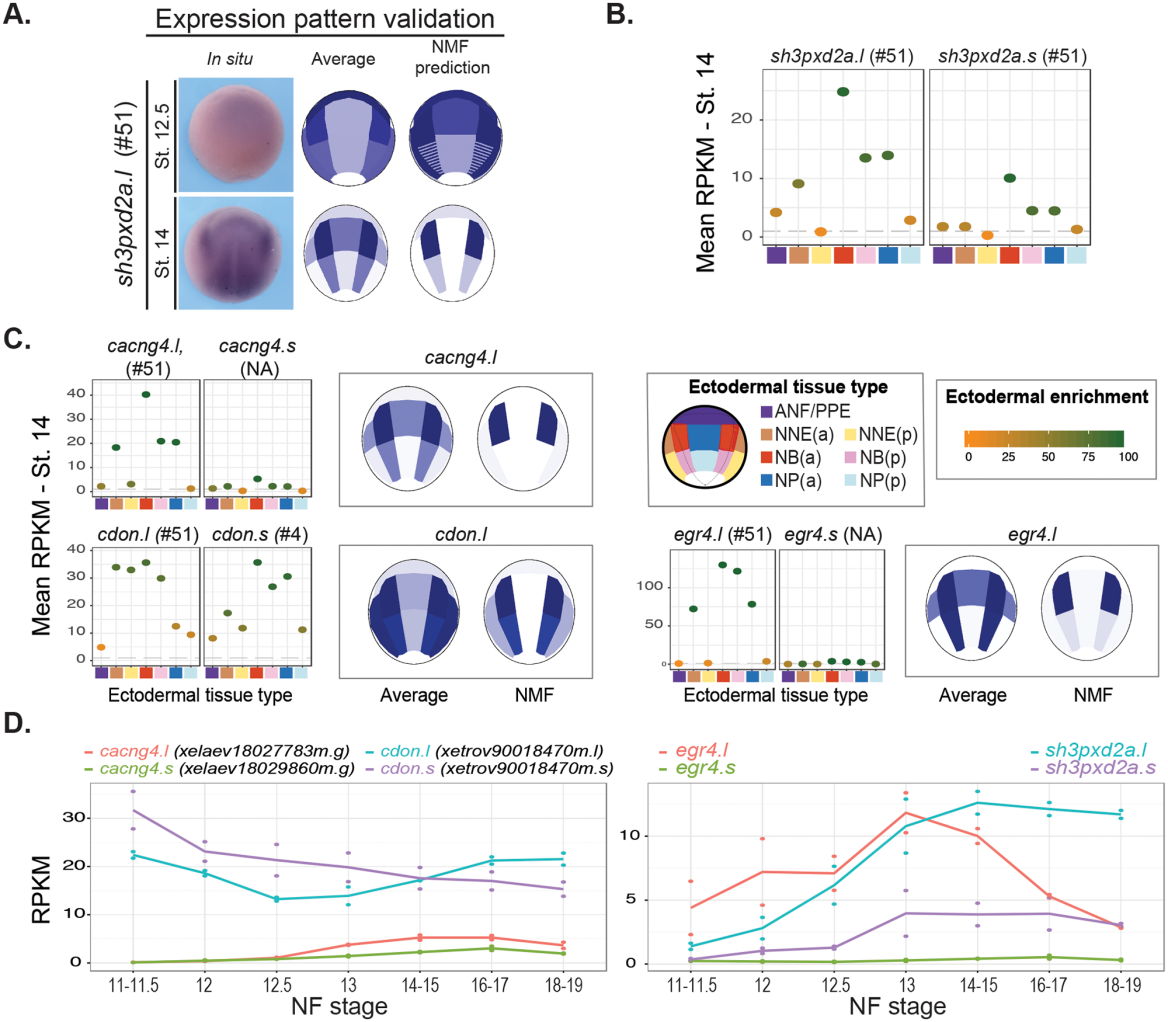
Genes with predicted expression and functional association to the neural crest (NC). (A) Validation of the expression pattern of *sh3pxd2a* at Nieuwkoop and Faber stages (St.) 12.5 and St. 14. Upon prediction of NC expression by nonnegative matrix factorization (NMF), we cloned a probe for novel gene *sh3pxd2a* and analyzed its in vivo pattern: while expressed at very low levels (B, 25 reads per kilobase per million [RPKM] in the anterior neural plate border [NBa] St. 14), it was readily detected, at low level, in the prospective NC in vivo. (B) Average RPKM expression per tissue dissection highlighting *sh3pxd2a* ectodermal enrichment in the NBa. Expression level for each homeologous gene copy is indicated. (C) Expression pattern predictions for 2 novel genes grouped in G-51, a group associated with NC development: *cacng*, *cdon*. *Egr4* was recently shown expressed in cranial NC [35]. Mean expression of both homeologous copies is indicated for each dissected region. (D) Temporal expression during neurulation (mean expression levels in whole embryos) for each homeologous copy of *cacng4*, *cdon*, and *egr4*. See S11 Table for numerical data.

We next investigated the efficiency of WGCNA clustering on a list gathering genes expressed in progenitors of neural tube, NC-GRN, and placodes (PPE-GRN) (S7C Table, [10,24,48,51–54]. The regional and temporal segregation of these progenitors during neurulation should be retrieved in our dissections as shown by PCA or NMF analyses. In total, we tested the distribution of 99 transcripts, encoding 56 vertebrate homologs across the 140 WGCNA groups (S7 Table). The selected transcripts were overrepresented in G-38 and G-51 (Fig 6B, S7D Table). G-38 contained 43% of input placodal transcripts and other genes required for forebrain and anterior development (*fzd*, *lhx2*, *rax*, *fezf2*, S4 Table) [55–58]. This grouping was thus similar to NMF deconvolution for ANF/PPE and matched with forebrain and placodes development. G-51 gathered 70% of input NC genes (Fig 6B, S7 and S10 Tables). We then looked for the spatial expression of G-51 genes not present in the input list. Published in situ hybridization experiments confirmed that 87% of the G-51 annotated transcripts were expressed in the pre-migratory NC (S7D Table). Moreover, the NMF analysis predicted that the remaining uncharacterized G-51 genes were expressed in NC progenitors at St. 14 (Fig 7, S11 Fig). Finally, we cloned a PCR fragment of *sh3pxd2a*, one of the uncharacterized genes in G-51, and confirmed its NC expression in neurula-stage WEs, as predicted by both WGCNA and NMF (Fig 7). Interestingly, this gene was expressed at very low levels (mean of 20 RPKM in NBa, compared to 1,000 RPKM for *snail2* in the same tissue, Figs 4 and 7); it would probably not have been picked up by an approach based on in situ hybridization in vivo, showing how our large-scale analysis followed by pattern predictions could help in identifying novel genes of interest. Also, in this case, due to low expression levels, the average pattern was much less clear cut than the NMF-deconvoluted pattern (Fig 7).

All together, these results show how a list of genes, taken either from a broadly used pathway (Wnt) or merged from diverse pathways and GRNs (neural, NC, placodes, mesoderm patterning, and various signaling), was distributed across the WGCNA groups. As a whole, this grouping displayed biologically relevant co-expression groups from the temporal and spatial profiles extracted from our neurula dataset. For further research, we provide a list of all WGCNA groups (S7B Table). Moreover, we concluded that the combination of WGCNA, GO enrichment analyses, and NMF was a powerful and reliable approach to point out interesting gene candidates from large-scale genomics datasets and prioritize gene analysis.

### Differential expression and WCGNA identified spatial and temporal variation in homeologous genes expression

*X*. *laevis* genome is allotetraploid, with 56% of the genes retained as 2 copies, named homeologous genes [16]. Polyploidy, while rare in amniotes, is frequently found in amphibians, fish, and plants. *X*. *laevis* genome arose from an interspecific hybridization between 2 diploid progenitor species about 17–18 million years (My) ago [16]. The chromosomes originating from each species are identified and named L (long) and S (short):.l or.s suffix is added accordingly to each gene name. This redundancy in the gene set may relax functional constraints on the 2 copies of a gene, either in protein or in regulatory sequences. In the latter situation, this could result in variation in gene expression. So far, a few studies have compared homeologous gene expression levels in developing WEs; no study of spatial expression was available [59,60]. We have here used 2 complementary approaches to evaluate differential expression in homeologous gene pairs.

First, we used WGCNA clustering to get a global look at differences in homeologous gene expression by assessing their co-clustering. In the gene set used for WGCNA, we identified a subset of 2,520 homeologous gene pairs, which exactly matched to the same HUGO name (S10 Fig, S8B Table, [16]). This approach detected 740 gene pairs found in the same WGCNA group (29%, S8 Table). Many of these pairs encoded transcription factors, in agreement with [59] (S11 Fig). This indicated that the remaining 1,780 pairs exhibited expression differences (S8 Table). When a few transcription factors were evaluated qualitatively on spatial dissections and temporal WEs profiles, most tested genes showed comparable expression for homeologous pairs (e.g., *pax3*.*l* versus *pax3*.*s* and six3.l versus six3.s; S11 Fig), but some showed clear differences in spatial and/or temporal profiles (e.g., *snai2*.*l* versus *snai2*.*s* and *six4*.*l* versus *six4*.*s*; S11 Fig). Interestingly, a closer look at Snail2.l and Snail2.s protein sequences revealed increased amino acid substitutions associated with reduced transcript level in Snail2.s, suggesting that a relaxation of functional constraints is occurring on this copy (S12 Fig).

**Fig 8:**
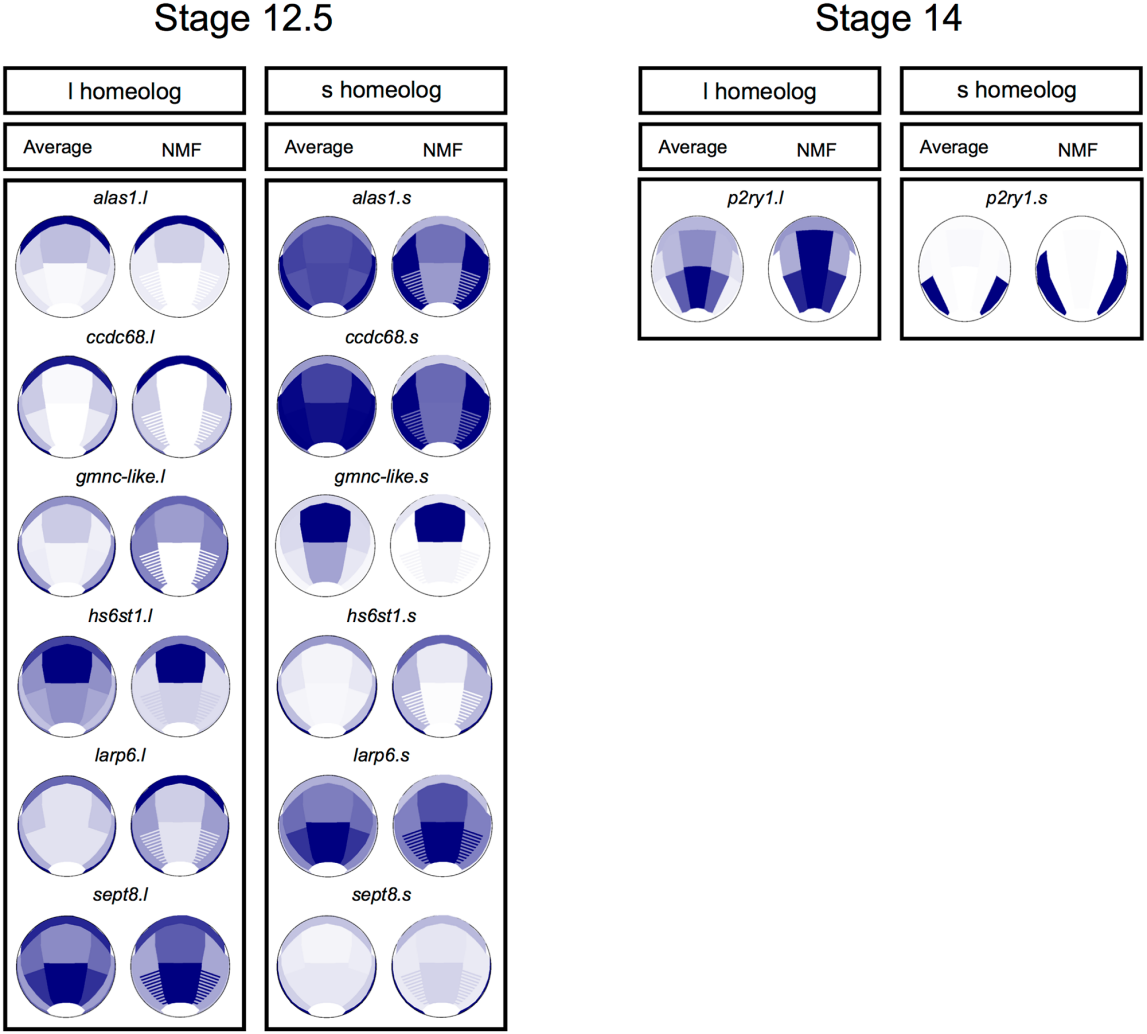
A few *Xenopus laevis* homeologous gene pairs display spatial subfunctionalization. We have studied how homeologous genes are expressed in the whole dataset (using weighted gene co-expression network analysis [WGCNA]) or in the dissected regions. We found that most pairs with differential expression exhibit asymmetrical decrease of 1 copy (e.g., *egr4*, *sh3pxd2a*, *snail2*, Fig 7, S11 Fig). We found 5 pairs with spatial differential expression, suggesting spatial subfunctionalizations: at Nieuwkoop and Faber stage (St.) 12.5, *alas1*, *ccdc68*, *gmnc-like*, and *hs6st1* and at St. 14, *p2ry1*. Average and nonnegative matrix factorization (NMF) expression patterns are shown at the relevant stage.

Differential expression could reflect 2 different phenomena: the first is decreased expression of 1 homeologous copy, while the other copy retains expression (asymmetrical decrease), which is a means for maintaining gene dosage in polyploid species; alternatively, differential expression could reflect genuine spatial subfunctionalization. To get a more quantitative view and identify precise spatial subfunctionalization during ectoderm formation, we used differential expression (*p* < 5%) between homeologous pairs in each dissected ectoderm region at either stage. In order to avoid detecting differences in expression level due to variations in gene size, we selected the 2,001 pairs whose gene length differed by less than 20%, with at least 10 counts, from the whole 7,949 homeologous gene list. Among these, 1,036 gene pairs were differentially expressed within at least 1 dissected region at St. 12.5 and 1,118 pairs at St. 14. When differential expression between at least 2 dissected regions was tested in order to identify subfunctionalization from asymmetrical decrease [61], we found 640 gene pairs at St. 12.5 and 602 pairs at St. 14. At St. 12.5, 634 homeologous pairs exhibited asymmetrical decrease. This decrease significantly affected the S chromosome in 364/634 of the pairs, (*p* < 0.01%). At this stage, we found only 6 gene pairs with spatial subfunctionalization in the ectoderm (*alas1*, *ccdc68*, *gmnc-like*, *larp6*, *sept8*, and *hs6st1*). All but *gmnc-like* were also found in different WGCNA groups above (S8 Table). NMF pattern predictions for these genes are shown in Fig 8. At St. 14, similar analysis found 601 gene pairs showing asymmetrical decrease (with S chromosome copy down-regulated in 336/601 cases, *p*-value < 0.2%) and a single gene (*p2ry1)* with spatial subfunctionalization (Fig 8).

We concluded that these 2 complementary approaches, spatial differential expression analysis and WGCNA, highlighted heterogeneity in gene expression among homeologous pairs resulting from *X*. *laevis* genome evolution. In-depth analysis provided temporal and spatial resolution for differential expression within a given gene pair. Asymmetrical decrease was observed in the vast majority of cases, with diminished expression occurring statistically more often for copies located on the S chromosomes. Finally, tissue-specific gene subfunctionalization was observed in a few rare cases.

### A gene co-expression network enhanced clustering and highlighted potential functional relationships between clusters

In an effort to expand and customize the previous analysis, we implemented a web interface to query and visualize the gene co-expression network from a user-defined list of seed transcripts. This retrieved the co-expression relationships between the genes of interest (seed) and all transcripts in the dataset that shared strong correlations (positive or negative). In particular, this would allow visualizing the relationships between genes from different co-expression groups. To create the network, the user simply specifies the list of gene names and a minimal *p*-value cutoff that will be used to determine the number of transcripts (nodes) connected with each of the inputs. The large nodes in the network represented each of the input transcripts. The lines (edges) connected 2 correlated transcripts: a thick line for a negative correlation and thin line for a positive correlation. The color of each node corresponded to the gene co-expression group determined by WGCNA in the previous section. With such network, one could explore the hierarchical temporal and spatial relationships between the factors involved in neurula ectodermal patterning, as exemplified below.

**Fig 9:**
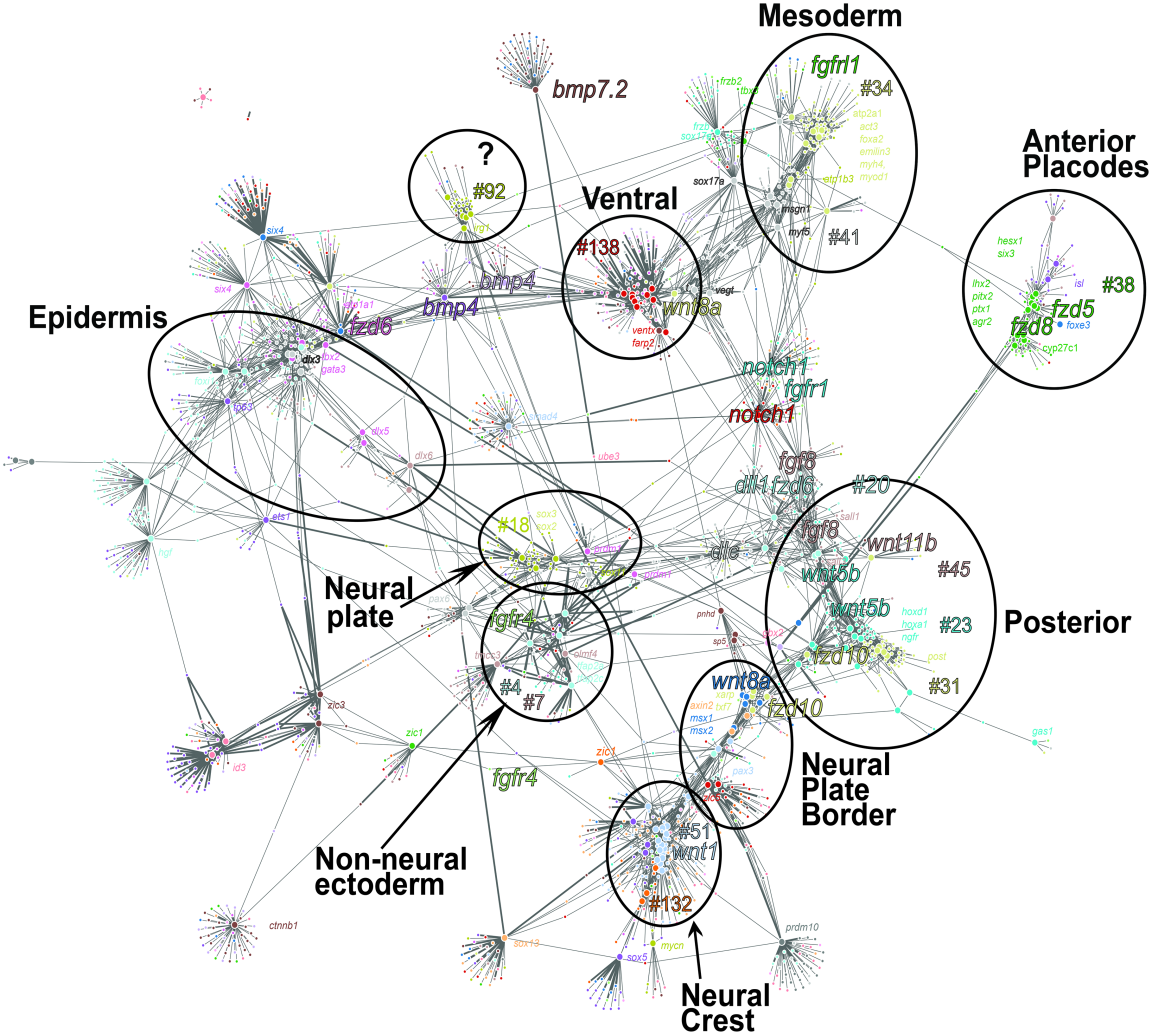
Gene co-expression network of *Xenopus* neurula visualized biologically meaningful relationships between weighted gene co-expression network analysis (WGCNA) groups and subsets of genes. We obtained a gene co-expression network using a large input gene list, including Wnt-related transcripts, neural, neural crest (NC), placode, and mesoderm genes as input nodes (large points, S10 Table). The *p*-value cutoff <1E-09 generated 2,001 nodes (small points indicate closely correlated genes; the color of the points refers to a given WGCNA group) and 4,945 edges. Circles are drawn to groups of genes with close spatial/temporal expression patterns. The group was named based on known members of the group. Groups are linked to other groups with positive (thin line) or negative (thick line) correlation. As an example, NC genes (groups #51 and #132) and placodes (group #38, in green) display negative correlation of expression with posterior genes (groups #23, #20, thick lines). The network also indicates interactions between ventral genes such as *ventx* genes (group #138), positively correlated with *bmp4* and *bmp7*.*2* (thin lines). When running the EctoMap application, the network is interactive and indicates each gene name upon selection.

We challenged the gene co-expression network by entering a large list of genes, mixing several distinct biological processes, and testing how they would be subgrouped by unsupervised clustering: we included 54 genes (large nodes) associated with various signaling pathways (bone morphogenetic protein [BMP], Wingless-Int [WNT], and fibroblast growth factors [FGF] pathways), neural patterning, placode GRN, NC-GRN, mesoderm patterning, and endoderm formation (S10 Table). With a *p*-value cutoff of 1E-09, we obtained a network comprising 2,001 nodes and 4,945 edges (Fig 9). As expected, the network construction grouped genes from the same WGCNA cluster and linked them to other biologically relevant clusters. For example, the NC groups (G-51 and G-132) were close to the NB genes (*msx1/2*, *pax3*, *tfap2a/c*, and *zic1/5*), which were positively connected to their inducers, Wnt and FGF (in G-23, G-31, and G-45): this close linkage reflected relationships within the NC-GRN. Similarly, the genes downstream of canonical Wnt signaling, required for posterior tissue specification (G-23, G-41), positively associated to several posterior signaling pathways, Wnt (G-31, G-95, e.g., *wnt8a* and *wnt3a*), retinoic acid (G-23, e.g., *rara*), and Fgf pathways (G-45, e.g., *fgf8*) [62,63]. In contrast, negative correlations were observed between posterior (G-23) and anterior expressed genes (e.g., forebrain and anterior sensory placodes, G-38). We concluded that the grouping of known genes agreed with their related function. This strongly suggested that this network, based on spatial and temporal gene synexpression during neurulation, would predict meaningful novel relationships between known and unknown genes at the genome scale.

### Comparing human cancer-related genes to developmental gene co-expression facilitates data mining of large-scale datasets

Finally, we evaluated whether this neurula embryo dataset, with its accompanying data-mining tools, could shed interesting light on other types of genome-scale approaches, either in cell biology or human pathologies. Genome-wide association studies (GWASs) aim to identify genes correlated with a specific phenotype or pathology, with the goal of identifying diagnosis markers [64]. One of the biggest challenges of a GWAS is to identify the genes primarily driving the pathology, among thousands of altered genes, and to understand how these contribute to disease development. We propose that the GWAS could be coupled with the developmental co-expression networks to get a better understanding of the etiology of complex traits. Because our samples collection focuses on ectoderm, this dataset would be most suitable for phenotypes associated with ectodermal-derived neonatal disorders (e.g., neurocristopathies, ciliopathies, neural tube defects), tumors of ectodermal origin (i.e., melanoma, schwanoma, neuroblastoma), and pathologies related to epithelium-to-mesenchyme transition (EMT) such as cancer and fibrosis [1,65].

Melanoma is an aggressive, NC-derived tumor, initiation of which involves a NC-like state, i.e., the reactivation of early, neurula-stage NC-specific genes such as *sox10* in adults [47]. This correlates with the reactivation of "super-enhancers" proximal to NC genes. Super-enhancers are regulatory elements highly enriched in active chromatin marks (K27-acetylated histone 3 [H3K27Ac] mark) and involved in cell differentiation and cancer [66,67]. The presence of super-enhancers close to the *sox10* gene was associated with elevated *sox10* expression both human melanoma cells and in a zebrafish melanoma model [47]. Additionally, these authors provided a list of 843 super-enhancers present in human melanoma cell lines in close proximity to 778 other human genes. Here, we asked if we could extract information on the genes associated with those super-enhancers using the gene co-expression network derived from the neurula-stage embryo (S9 Table).

**Fig 10:**
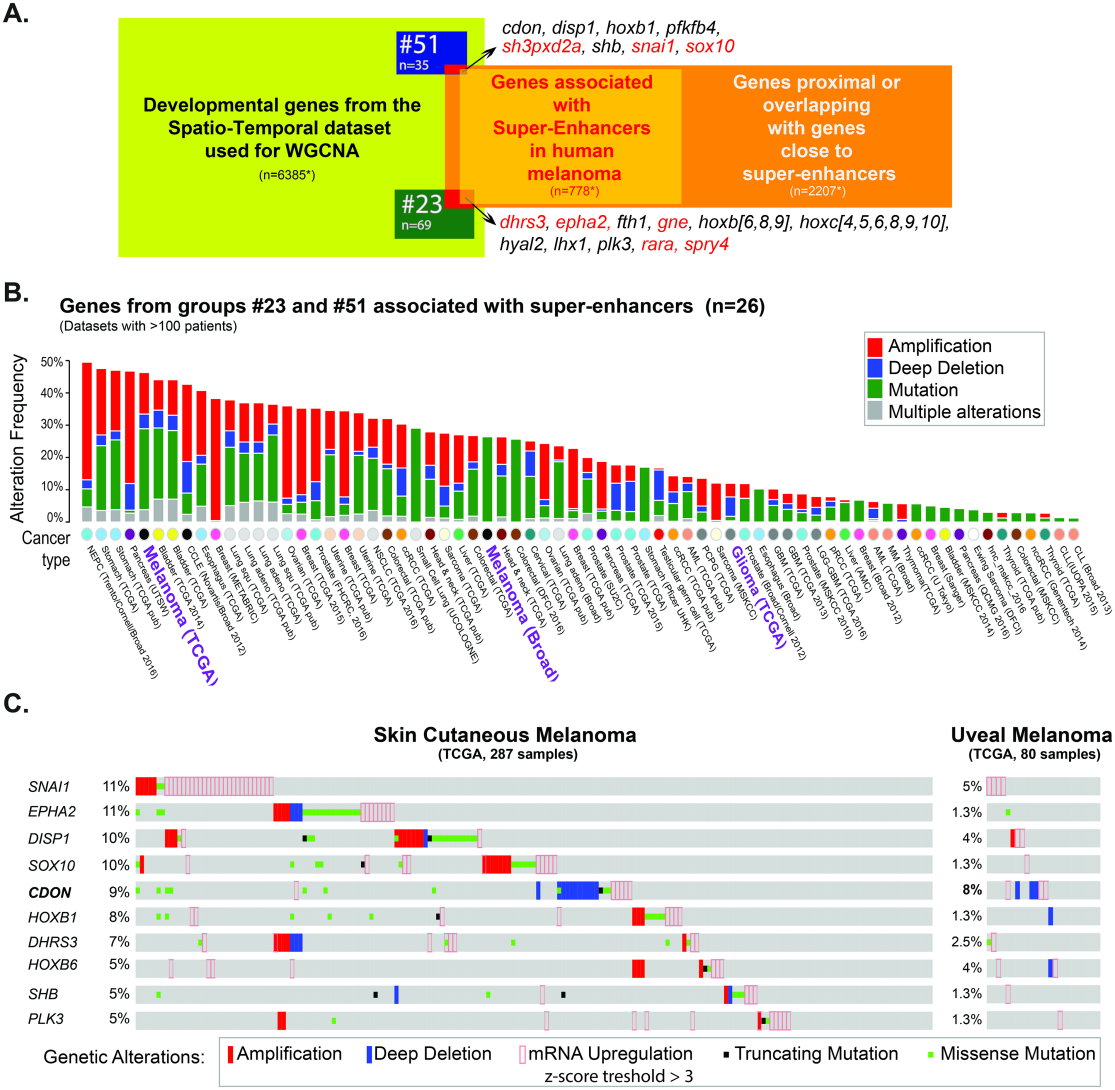
Genes linked to human melanoma are tightly associated with Wnt-associated genes and neural crest (NC) genes during development. (A) Several (26) genes linked to melanoma super-enhancers defined by Kauffman et al. (2016) were found among the posterior/Wnt genes group (#23) and the NC group (#51). (B) Cumulative frequency of genetic alterations found in these 26 genes in various cancer types: NC-derived cancers are outlined in purple. High mutation frequency (>45%) is found in melanomas. The nature of the genetic alteration is plotted along y-axis. We used 70 studies describing at least 100 tumors of each kind (TCGA). (C) These 26 genes were frequently mutated in cutaneous and uveal melanoma: this diagram displays genetic alterations found for each gene above 5% frequency, in 191 out of 287 cutaneous melanomas and in 24 out of 80 uveal melanomas. See S11 Table for numerical data. *Panels (B) and (C) are based on data obtained by the TCGA Research Network*: http://cancergenome.nih.gov *and visualized by cBioPortal*.

We selected *X*. *laevis* genes corresponding to human gene names in immediate proximity with the top super-enhancers in human melanoma cell lines (ranked by enrichment levels of H3K27Ac) [47]. Sixty-five *Xenopus* orthologs were expressed in our neurula-stage developmental dataset. Using WGCNA, we found that transcripts highlighted in red were significantly enriched in groups associated with posterior patterning and canonical Wnt signaling (G-23, *p*-value = 3E-02) and NC (G-51, *p*-value = 2E-02) (Fig 10A). In contrast, no enrichment was found for the 64 genes close but not immediately adjacent to the top 200 super-enhancers ([47], not shown). We then asked the complementary question: which of the genes associated to the 843 super-enhancers defined in human melanoma were part of G-23 and G-51, and found 26 genes falling in these groups (Fig 10; S9 Table). This analysis further supports the notion that melanoma initiation, WNT signaling, and NC development share common molecular signatures [47,68] and supports investigating the roles of uncharacterized genes either in NC development or during melanoma initiation.

To investigate how often these 26 genes were altered in cancer, we looked in public repositories of human tumor datasets and found that melanomas have one of the largest proportions of genomic alterations in at least 1 of these genes (Fig 10B). When focusing only on melanoma patients, we observed that the vast majority of alterations correspond to up-regulation or amplification events [69–72] (Fig 10C). We found complex results for 1 of the genes, *CDON*, as it appeared either up-regulated (>2%) or deleted (>3.5%) in uveal and skin cutaneous melanoma (Fig 10C). Deletion of both copies of CDON was also commonly observed in other types of cancer: 11% of testicular germ cell cancer and 5% of cervical cancer (TCGA Research Network: http://cancergenome.nih.gov [TCGA] not shown). Such mixed observations, in adults, have complicated the understanding of CDON function. Cdon serves as a multifunctional co-receptor with different roles in signaling transduction. On the one hand, it acts as co-receptor for Hedgehog proteins; on the other hand, it interacts with N-cadherin to activate p38α/β mitogen-activated protein kinase (MAPK) [73]. Currently, the functional characterization of frog *cdon* is lacking, but our expression predictions and analysis on other model organisms [74,75] support potential roles of CDON in human melanoma. Our NMF expression pattern predictions localize *cdon* predominantly in the NB in neurula (St. 12.5 and St. 14) (Fig 7C). Likewise, the adjacency co-expression matrix and the network positions *cdon* in close relationship with other NC transcripts (e.g., *sh3pxd2a*, *sox9*, *rapgef2*, and *gdf5*), Sonic Hedgehog receptor (*disp1*), and genes related to Wnt signaling pathway (e.g., *axin2* and *xarp*). Hence, we conclude that a combined strategy of mining human cancer databases and searching our developmental gene co-expression network may be useful to prioritize gene candidates for further functional studies.

### Integration of the temporal and spatial transcriptomic datasets in EctoMap, a user-friendly online tool

**Fig 11:**
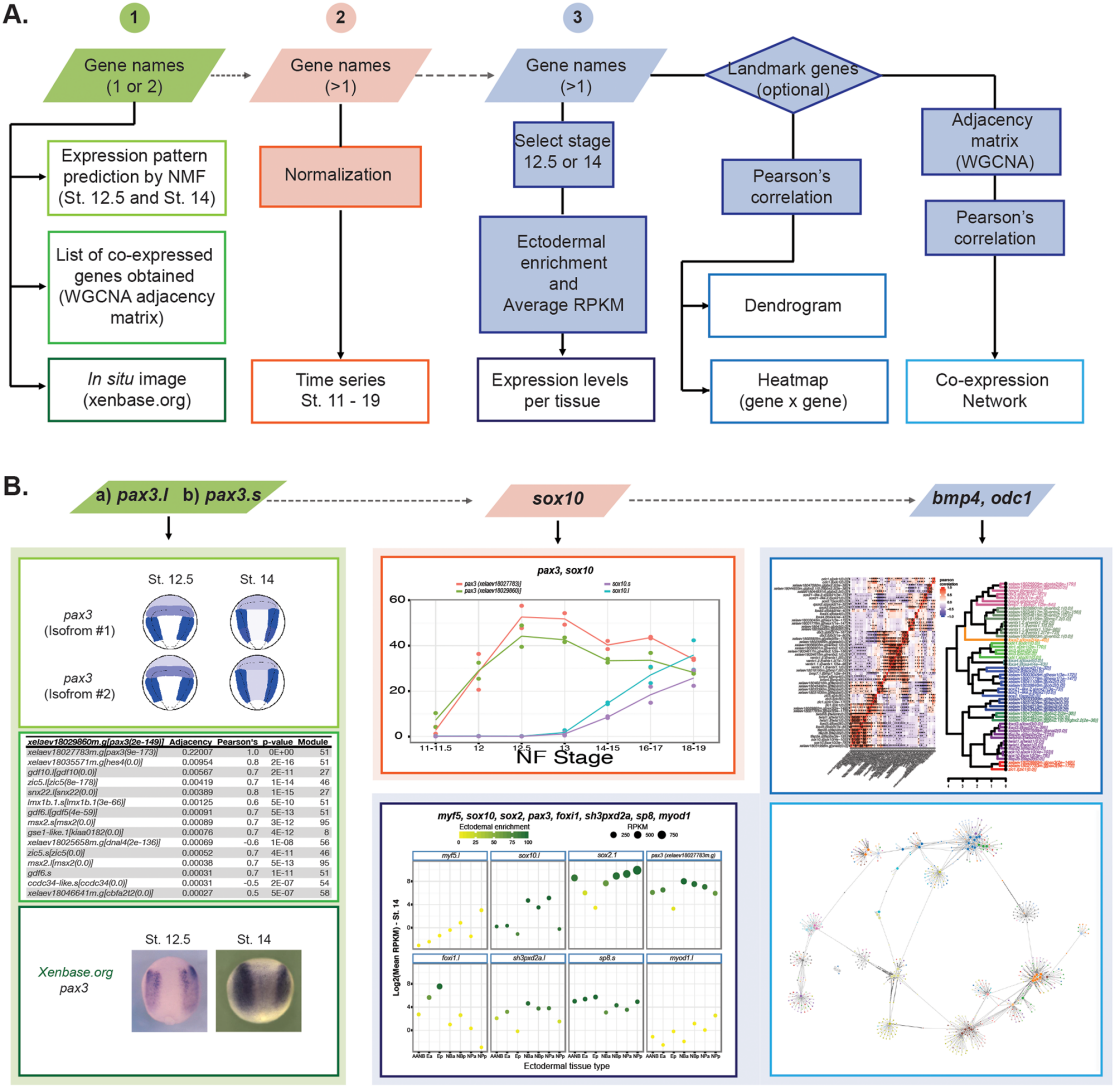
EctoMap: An application to explore gene synexpression during neurulation. (A) Workflow for the EctoMap application, indicating the input options and the files needed to produce the output plots. (B) Example of outputs (see also S1 Web Archive).

We integrated this large transcriptomic dataset as a comprehensive suite of interactive tools to explore the expression of 1 or more genes of interest (Fig 11). The application was called "EctoMap" and it was intended to allow users to explore gene expression levels over different developmental time points and tissues and retrieve expression pattern prediction obtained by NMF along with a list of co-expressed genes identified by WGCNA analysis (see example in S1 Web Archive). We also provided the interactive gene co-expression network, to which we recommend adding landmark genes for a more informative interpretation of the temporal and spatial relationships between genes. The tool was implemented in R using the Shiny-app and the dynamic network visualization using the Network-D3 library (please download the full application and run locally). A simplified version of EctoMap (EctoMap Lite) is made publicly available (https://monsoro-lab-ectomap.shinyapps.io/EctoMAP/).

## Discussion

In vertebrate late gastrula and neurula embryos, dynamic cell fate diversification is initiated in the ectoderm by progressive individualization of broad progenitor areas positioned along the A-P and D-V axes. These processes are highly conserved from basal vertebrates to mammals [76–78]. Before the definitive establishment of specific cell fate markers in the late neurula, no sharp boundary can be drawn in the ectoderm, as progenitor cells globally share substantial gene expression with cells from the neighboring areas. This property is inherent to the early neurula and precludes, so far, the reliable development and use of lineage-specific tracers. Rather, there is an urgent need to document the progressive spatial and temporal diversification of genome expression across the ectoderm during early neurulation. This study defines the regional transcriptome of a single germ layer, the ectoderm, at 2 developmental stages, early and mid-neurula. Microdissecting small ectoderm regions from single embryos for low-input RNA-sequencing allowed retaining precise spatial information for each sample. We provide a comprehensive searchable interface, EctoMap, integrating gene co-expression dynamics during ectoderm early patterning into progenitors for nervous system, skin, sensory placodes, and NC. EctoMap provides enhanced in silico quantitative pattern prediction and generates synexpression networks for any gene expressed during early neurulation. We characterized the regional and temporal molecular signature in each area of the neurula ectoderm, which corresponds to the regionalization of cell fates. Customized networks retrieve gene expression relationships relevant for developmental biology or other research fields including evolution, stem cells, or cancer biology.

### A molecular map of vertebrate ectoderm during neurulation

Besides WE transcriptome analyses, previous genome-wide gene expression studies in vertebrates using low-input RNA sequencing have focused on cleavage, gastrulation, or early organogenesis [5,7,8,11,79,80]. At the end of neurulation in particular, transcriptome of definitive NC cells has been obtained just prior to or during their migration [81–83]. Other studies have taken advantage of naive ectoderm reprogrammed in vitro into different ectoderm cell fates such as neural, NC, or placodes cells [10,84–87]. Because collecting small ectoderm-restricted areas in vivo was a technical challenge, this study is the first germ-layer specific transcriptome analysis in early neurula. We used the tetrapod *X*. *laevis* embryo, a classical and prototypical vertebrate embryo, allowing stereotypical in vivo microdissections of the ectoderm during neurulation in reference to known gene expression patterns and previous fate maps (Fig 1). We document the transcriptome-wide signature for spatially and temporally defined ectoderm areas at 2 developmental time points when critical cell fate decisions are made: at the end of gastrulation, as the ectoderm cells respond to key signaling cues from the surrounding cells and underlying tissues, and at mid-neurula stage, as the initial positional information has been established across the ectoderm and is followed by further subdivision of the ectoderm-derived tissues [4,77]. Hence, we report quantitative expression data for the 31,700 transcripts expressed in early neurula (i.e., 82% of the genome), corresponding at least to 11,616 unique human homologs (based on HUGO nomenclature). With the advent of single-cell transcriptomics, this study provides an unprecedented spatial reference and a deep sequencing coverage that are needed for analysis of isolated cells transcriptome.

### A transcriptome-scale referential for positional information in the developing neurula ectoderm

Unsupervised analysis of transcriptome variations between neurula-stage ectoderm regions might implicate diverse biological processes; for example, cell proliferation, polarity, metabolism, signaling, or patterning might distinguish ectoderm areas. To enhance sensitivity, we focused on genes with high variation in expression between different ectoderm areas. PCA revealed that the major transcriptome-level variations between 2 different neurula ectoderm areas were strongly and primarily related to their position along the D-V and A-P axes, rather than to other cellular processes (Fig 2). Hence, at the end of gastrulation and during neurulation, the differential transcriptional response of ectoderm cells to the surrounding developmental cues and signaling gradients was clearly readable in their transcriptome and was precisely related to their position in the plane of ectoderm. This was coherent with previous analyses for selected genes [76,88–91]. This result allows precise spatial positioning of any given ectoderm sample, based its transcriptome coordinates, prior to morphological feature appearance in the ectoderm. In addition, we provide gene signatures correlated to the D-V and A-P axes (S4 Table, S4 Fig). The third PCA component was ectoderm subregionalization, with the emergence of the NB, identified as a separate territory, supported by the expression of transcription factors such as *pax3*, *snail1*, *sox9*, and *msx* in the late gastrula. This confirmed previous analyses showing NB emergence at the end of gastrulation, characterized by *pax3/7* expression [18,19,36,92]. Known and novel gene expression also correlate with this third PCA component. Moreover, the timing of cell specification into different progenitor cell types was also readable in their transcriptome and identified by PCA. The novel gene functions supporting this temporal differentiation remain to be explored. We conclude that similar use of transcriptome coordinates could allow precise spatial allocation in the ectoderm plane, either for wild-type ectoderm samples (e.g., selected ectoderm areas or ectoderm single cells at similar or different developmental stages) or for understanding the genome-scale modifications following mutation or other experimental gene perturbations. A similar strategy could also be used to understand the main features of embryonic tissues undergoing precise patterning within morphogenetic fields [88].

### An enhanced atlas of individual gene expression and regional gene signatures across the developing ectoderm

Next, we provided a comprehensive quantitative atlas of individual gene expression in the single ectoderm germ layer, enhanced by NMF deconvolution. We anticipate that this repertoire will be of broad use in vertebrate embryos, given the high conservation of developmental mechanisms and gene expression patterns in vertebrates, in general, and in tetrapods, in particular [93]. We have compared the usual averaging strategies (i.e., averaging expression level between biological replicates for a given dissected region) to an unsupervised approach using NMF, designed to define homogeneous domains in the neurula ectoderm, independently of potential variations between replicate samples for a given dissected region, especially in the case of closely located regions. Using this approach, we provide a better estimate of gene expression patterns than by simple averaging for complex regions such as the NPa and NBa at St. 14 (Fig 4). Both average patterns and NMF patterns are coarse-resolution renderings of fine gene expression patterns and are meant to give a quick and global view of gene expression in an ectoderm domain, which cannot reach the refinement of an in situ hybridization pattern. However, we can now predict the pattern of all 31,700 genes expressed during early neurulation.

When compared to NMF patterns, average patterns are more sensitive to variability in dissections. The main example is for NBa at St. 14, the average patterns for which show larger expression than the in situ hybridization pattern (Figs 4 and 7, S8 Fig). In St. 14 embryos, NBa, NPa, and ANF/PPE are closely located in the ectoderm, and the corresponding samples tend to display dispersion (as shown by PCA or tSNE). In this case, average patterns will show contribution to NPa and ANF/PPE, even for genes expressed only in NBa (e.g., NC-specific genes *snail2* and *sox10)*. In contrast, NMF distinguishes neatly NBa from NP and ANF/PPE, because it is designed to deconvolute contributions between neighboring tissues. In this case, NMF pattern is closer to in situ hybridization pattern. For example, *snail2/st14* is indicated in NBa only, while a gene such as *sox2/st14* is predicted in NP, ANF/PPE, and at lower level in NBa/p, very similarly to average and NMF patterns. On the other hand, NMF is less sensitive than other approaches to discriminate some dissected regions; it does not distinguish between NBp and NPp at St. 12.5 or between NPa/NPp and anterior NNE [NNE(a)]/posterior NNE [NNE(p)]. In this case, average pattern combined with ectoderm specificity index (to indicate potential contribution of underlying mesoderm) would provide better information.

We designed a searchable web tool, EctoMap, to share this information, using human gene name nomenclature (HUGO names) and the latest genome annotations. For each gene, EctoMap enables an immediate visualization of semiquantitative gene expression pattern in the 7 key ectoderm regions defined for deep sequencing, at early and mid-neurulation stage, and of gene expression dynamics during neurulation in WEs. This predicted pattern is accompanied by an average pattern and an ectoderm specificity index for each region to provide maximum information in queries regarding unknown gene spatial and temporal expression.

As orthologs for over 79% of genes implicated in human disease are conserved in frog [16,94], the discovery of novel genes implicated either in cancers derived from ectoderm or during stem cell reprogramming into ectoderm-derived fate could prompt immediate in silico analysis of their expression during development, even in absence of in situ hybridization data in the current databases.

Importantly, NMF defined 5 basic areas from the transcriptome of the 7 dissected domains, unlike PCA, which discriminated all 7 tissues at each stage. Theoretically, this could either reflect mixed cellular contributions in the dissected samples, due to slight spatial variations between replicates or due to cell heterogeneity within the tissue, or it could reflect that a given ectoderm domain transcriptome can be explained by a combination of 2 basic transcriptomes. Future single cell transcriptome analysis should help in resolving between these options.

With NMF, we also defined a specific gene signature for each area using the Gini coefficient [42] to evaluate enrichment in gene expression for each domain. We retrieved a characteristic gene signature defining each NMF-predicted ectoderm domain (S5 Table, S7 Fig), thus providing the first genome-scale unsupervised annotation of the different ectoderm spatial domains. These results will allow the derivation of accurate area-specific lineage reporters for these early neurula stages. A recently study in late chick neurula identified a few transcription factors controlling NC heterogeneity along the A-P axis, based on their differential expression in transcriptome datasets [95]. Similarly, but in earlier embryos, our spatial and temporal ectoderm gene signature should stimulate functional analyses on differential fate determination during early stages of neural, NC, and placode formation.

Finally, *X*. *laevis* genome arose from recent genome allotetraploidization [16], with 56% of genes retaining 2 copies from the ancestral genomes. EctoMap provides spatial and temporal expression for each homeologous copy. Using spatial differential expression, we found that about half of homeologous pairs display significant differential expression due to asymmetrical decrease, a potential means to maintain gene dosage. Only a handful of pairs were subjected to spatial subfunctionalization (Fig 8, S11 Fig, S8 Table). This strong bias towards asymmetrical decrease is also found in mammals after gene tandem duplication [61]. Tissue-specific gene subfunctionalization could involve complex context-specific regulation of gene expression and potential creation of novel functions for 1 gene in the homeologous pair. In both cases, our gene lists could allow investigating the evolution of gene regulatory elements, following a 17–18 My-old whole genome duplication, as compared to older genome duplications in vertebrate or teleost lineage.

### A dynamic co-expression network to explore gene synexpression during neurulation

During development as well as in adult life, control of gene co-expression is an efficient and widely used way of coordinating spatial and temporal activity of groups of factors involved in related functions. Consequently, gene synexpression in time and space may indicate potential related activity between a set of factors. We thus completed this analysis by unsupervised WGCNA clustering and co-expression network analysis. We retrieved gene groups and validated the biological relevance of several groups, including Wnt-responsive gene group, premigratory NC group, and placode group. Further analyses of our clustering would shed light on potentially related molecules in multiple pathways or processes. As an example, we shortlisted interesting candidates among novel genes involved in melanoma based on their synexpression with NC, Wnt signaling, and EMT-related factors in neurula. Finally, we have developed an online searchable network application allowing querying the synexpression and correlation among groups of genes. This query could involve genes acting in development, stem cells reprogramming, epithelium-mesenchyme transition, signaling, or various pathologies. We propose that the analysis of their expression in the developing ectoderm and the identification of their potential partners during neurulation will provide, with ease, novel and important insights into their biological roles and trigger relevant functional studies.

## Materials and methods

### Ethics statement

All animal care and experimentation were conducted in accordance with institutional guidelines, under the institutional license #C91-471-108, project # AP AFIS#7043–20 160930 14156646 v2 (Direction Départementale de la Protection des Populations, Courcouronnes, France).

### Embryos

*X*. *laevis* embryos were obtained by in vitro fertilization using standard procedures [96] and grown until the desired stage according to the Nieuwkoop and Faber developmental table [17].

### Sample microdissection

The embryo dissections were done as described in [26]. To ensure accurate dissections, a drawing of *X*. *laevis* embryo was made with respect to known in situ hybridization expression patterns for *pax3*, *snai2*, and *sox2* at St. 12.5, St. 14, and St. 17. This ectodermal map is shown in Fig 1A. The ectodermal regions were dissected using an eyebrow knife inserted first between the mesoderm and ectoderm layer prior to cutting. The dissected tissue was immediately lysed in Trizol (Life Technologies). A single dissection from a single embryo was used for each library preparation, after assessing RNA quality and confirming tissue sample identity by qPCR. A total of 79 dissected fragments were selected out of about 300 individual dissections.

### In situ hybridization

Embryos were fixed and prepared for whole mount in situ hybridization according to [97]. Antisense digoxigenin-labelled RNA probes were used at a final concentration of 10 μg/ml. The following probes were used: *snai2*, *pax3*, *sox2*, and *foxi1e*[36,98–100]. PCR fragments for *sh3pxd2a* and *sp8* were cloned from neurula stage cDNA and used as probes.

### RNA extraction and quality control

Total RNA from each individual dissected tissue was isolated by Trizol extraction (Life Technologies) with a step of shearing through a 25G needle. The aqueous phase was recovered using Phase Lock Gel (Eppendorf). Nucleic acids were precipitated with isopropanol and Linear polyacrylamide (LPA, Ambion), then washed in 70% Ethanol. RNA was resuspended in 30 μl RNAse-free water (Ambion) and dosed using a Nanodrop 2000 (Thermo Scientific). RNA quality was assessed on Bioanalyzer 2100 chips (Agilent).

### Quantitative real-time PCR

Reverse transcription was used M-MLV-Reverse Transcription (Promega). Quantitative real-time PCR (qPCR) reactions used SsoFast EvaGreen Supermix (Bio-Rad) on a C1000 thermal cycler (CFX96 real-time system, Bio-Rad). We quantified the relative expression levels of several dorsal ectoderm markers (*sox2*, *xk81a1*, *pax3*, and *snai2*) and a mesodermal marker (*myod1*) in order to confirm the sample ectodermal identity and detect a potential mesoderm contamination. Relative expressions were normalized using reference markers (*odc1* and *ef1a1*). Sequences for qPCR primers are listed in S2 Table.

### RNA sequencing and gene expression quantitation

Samples were selected for RNA library preparation if they met the following 3 requirements: (1) a ratio of the ribosomal 18S over 28S greater than 1.7-fold on bioanalyzer total RNA traces, (2) a confirmed tissue identity, and (3) a total RNA amount greater than 40 ng. RNA-seq libraries were prepared using the TruSeq Stranded mRNA library preparation kit (Illumina), starting with 40–300 ng of total RNA and sequenced on a HiSeq 2000 (Illumina) with a target of 15–20 million 100 bp paired reads per sample. Reads were mapped on the *X*. *laevis* genome (9.1) [16] using tophat 2.0.14 [101], with the following parameters (—rg-library Illumina—rg-platform Illumina—keep-fasta-order -N 6—read-gap-length 6—read-edit-dist 6—segment-mismatches 3 -i 5 -I 500000 -r 155—mate-std-dev 80—no-coverage-search -p 6—library-type fr-firststrand -g 2). 92% of the reads mapped on *X*. *laevis* genome. Paired reads mapping multiple times on the genome (approximately 4% of them) were excluded from the downstream analysis, leaving 88% of the reads for analysis. Putative transcript models were predicted using cufflinks 2.2.1 with the following parameters (—library-type fr-firststrand —3-overhang-tolerance 0—intron-overhang-tolerance 0), then merged with the annotation of the *X*. *laevis* genome (annotation version 1.8 of the 9.1 version of the genome, Xenbase ftp://ftp.xenbase.org/pub/Genomics/JGI/Xenla9.1/) using cuffmerge v2.2.1 [102]. Transcript annotation was done with a custom script BLASTing transcript nucleotide sequences against the *X*. *tropicalis* transcript nucleotide sequence database (JGI 4.2) [94] retaining only the first hit for each transcript with an E-value lower than 1E-100. Gene models and annotations were computed by merge overlapping transcript models and their annotation using BedTools [103] and a custom Perl script. Read counts were computed on each gene model using HTSeq [104]. Only genes with at least 10 counts in at least 2 samples were further considered. Read counts were then normalized using library size with trimmed mean of M-value normalization [105], as implemented in EdgeR [106], then transformed to log2-CPM (counts per million), and processed by the limma/voom package [107] to estimate the mean-variance relationship of the log-counts for subsequent statistical analysis. The data discussed in this publication have been deposited in NCBI's Gene Expression Omnibus [108] and are accessible through GEO Series accession number GSE103240 (https://www.ncbi.nlm.nih.gov/geo/query/acc.cgi?acc=GSE103240).

### Gene filtering

Many genes display little variation in expression across dissected samples (S1E and S1F Fig). For such genes, variations in expression between samples will be dominated by technical or biological noise. To filter out these genes, we have tested 3 commonly used methods: the range of log2(expression) (range), the variance (var), and the interquartile range (iqr). We have used the silhouette number (a clustering quality measure, https://cran.r-project.org/web/packages/cluster/) to assess how samples cluster according to their dissected region in the space defined by the first 3 PCA components. Silhouette number allows us to compare these 3 methods and define the appropriate number of genes to select (S1A and S1B Fig). All 3 methods gave similar silhouette profiles as a function of number of genes, with a sharp drop as genes with lower differential expression were included (S1A–S1D Fig). We used these curves to define that between 60 and 1,200 genes should be selected for St. 12.5, and between 1,100 and 6,000 genes at St. 14. The highest ranked genes were thus selected, using their range of log2(expression) and a minimum range threshold of 5, at both stages; 1,174 genes and 1,859 genes were selected at each of St. 12.5 and St. 14, for a total list of 2,198 genes.

### PCA, tSNE

PCA was computed with the standard R library on the sample expression vectors. The gene expression matrix was normalized by subtracting from each gene expression vector its average across all samples considered and dividing it by its standard deviation. To identify significant eigenvalues and genes displaying significant loading in each eigenvector, we generated a null distribution of eigenvectors by scrambling sample expression vectors through random permutation and computing PCA eigenvectors on the scrambled dataset 10,000 times. We inferred from this null distribution the 1% *p*-value threshold for each eigenvalue and the range of significant loading values for each eigenvector.

tSNE (perplexity parameter = 7 or 9) plots were computed from the gene expression matrix using, respectively, the R tSNE package Rtsne version 0.11 (https://github.com/jkrijthe/Rtsne) and the cmdscale function for the R standard library (package stats).

To help visualizing sample position along PCA components and in tSNE plots and whether samples from the same dissected region grouped together, we computed the barycenter of samples position for each tissue and drew the Voronoi tessellation of that set of points; this allows us to display the areas where points are closer to each barycenter in the plane of chosen PCA component or tSNE dimension.

### NMF matrix deconvolution

To compute the NMF deconvolution of the sample expression matrix (restricted to genes selected as differentially expressed at that stage), log2-transformed gene expression levels were converted back to expression levels and normalized by setting maximum expression of each gene to 1. NMF deconvolution was then computed on that normalized expression matrix with the R package NMF [109]. NMF rank was chosen by running the NMF algorithm on 20 random initializations of the component and mixing matrices and clustering rows of the resulting mixing matrices to visually identify if the algorithm converges robustly to the same minimum. The chosen NMF mixing and component matrices were computed as the median mixing and component matrices out of 100 runs with random initializations at the selected rank.

The computed component matrix contains only NMF-tissue expression data for the selected differentially expressed genes. We therefore computed an approximate measure of NMF-tissue gene expression for all genes by simply solving the NMF linear system (component matrix × mixing matrix = sample expression matrix) using the known sample expression matrix and the chosen mixing matrix and assigning all negative expression values in the estimated component matrix to 0. The approximate NMF-tissue expression values in this computed component matrix were found to be within 10% (relative to the highest expression level) of the expression values computed by the NMF algorithm for the selected differentially expressed genes.

NMF expression patterns were displayed on the NMF-tissue map by assigning the blue color to the NMF-tissue with highest expression and computing the relative color of other NMF-tissues on a white to blue scale using their relative expression level and the RcolorBrewer package (https://cran.r-project.org/web/packages/RcolorBrewer/). To measure NMF-tissue expression specificity and identify genes selectively expressed in a single NMF-tissue, we computed an inequality in gene expression distribution index using the Gini coefficient formula (https://en.wikipedia.org/wiki/Gini_coefficient). Among these, genes that did not display ectodermal enrichment (i.e., difference between highest median log2-expression in dissected tissues and median log2-expression in WE <0) were not included in S5 Table.

### WGCNA analysis

For the WGCNA analysis, we filtered out 80% of the transcripts with very low read counts across all 79 tissue and whole samples (S1 Table). We kept 10,073 informative transcripts out of a total of 50,974, as they passed 1 of 2 selection filters: the normalized CPM value for at least 1 of the all samples was above 50 CPM, or the absolute sum of normalized CPM values across all samples was greater than 40 CPM. The normalized CPM values from this set of “informative” transcripts were log-transformed and used as input for all WGCNA analyses.

To create the co-expression groups, we determined that a soft threshold power of 22 approximated the input data to a scale-free topology network (R^2^ = 0.91) and provided a mean connectivity of 3.92 (S9 Fig). From the signed adjacency matrix (power = 22, type = "signed," corFac = "bicor"), we calculated the dissimilarity topology overlap matrix (TOMType = "signed"), which was subsequently used to create a distance tree (method = "average"). To determine the co-expression groups, we dynamically cut the branches of the tree with a minimal cluster size of 15 transcripts per group (method = "hybrid", deepSplit = 4, pamRespectsDendro = FALSE, minClusterSize = 15).

### Biological process enrichment in WGCNA gene groups

To identify overrepresented GO terms in gene groups, we used "clusterProfiler v1.9" R library [110], which relies on the Fisher’s exact test. As background dataset, we provided a list of nonredundant genes from the frog genome, which was obtained by retrieving, for each of the *X*. *laevis* transcripts, the HUGO and corresponding Entrez IDs. All GO biological function terms used were from the ensembl88 human annotation database. We required *p*-value and q-value cutoffs of 5%, and the *p*-values were adjusted by applying the Bonferroni multiple sample correction test. S6B Table has GO biological functions associated with the WGCNA groups. Randomized datasets were produced by swapping the group assignments from each transcript while maintaining the number of transcripts per group, similar to those from the WGCNA. The complete list of GO biological functions enriched per WGCNA group can be found on S6B Table.

### Analysis of differential expression in homeologous gene pairs

We used the homeologous gene pairs defined by the *X*. *laevis* genome annotation 9.1 as duplicated copies of a gene present both in the long and short genome (7,949 pairs). Pairs that were not expressed (0 counts in both copies) were discarded. Because gene expression measured by counts depend on transcript length (longer transcripts generate more fragments for sequencing), we decided to compare only paired duplicates whose transcript lengths were close (<20% difference) so the comparison of their expression would not be affected by their length difference. At each stage, we then tested the difference in expression between duplicates for each tissue with the limma package [107] (5% *p*-value threshold corrected for multiple testing). Pairs for which 1 of the duplicates was significantly more expressed in at least 2 dissected regions and similarly expressed in the other regions were termed asymmetrically expressed pairs. Pairs for which 1 of the duplicates was significantly more expressed in at least 1 region and significantly less in at least another region were termed subfunctionalized pairs.

### Co-expression network

To create a customizable co-expression network, the user needs to provide 1 or more transcript names and select a *p*-value cutoff associated with the Pearson’s correlation (rcorr function of the Hmisc library from R). To visualize the gene co-expression network, we used the networkD3 library (https://cran.r-project.org/package=networkD3) in the R shiny app (https://cran.r-project.org/package=shiny). NetworkD3 creates a dynamic network after receiving 2 data frames, one that describes the nodes and the other one, the edges (links). Each transcript of interest (input node) is represented as a large node. The absolute value of the correlation coefficients defines the distance between the transcript of interest (input node) and its associated transcripts (associated node). Positive correlations are represented as thin lines, while negative correlations are represented by a thick line. The node colors are assigned according to the co-expression group obtained by WGCNA. The user can modulate the number of links by changing the *p*-value cutoff used for the correlation. To create such a gene co-expression network, we generated an unsigned adjacency matrix (power = 22, corFnc = "bicor") with WGCNA [44]. The unsigned adjacency matrix allows identifying positive and negative associations for each of the transcripts. For the network visualization, the program retrieves the 50 most adjacent transcripts and computes pairwise comparisons (Pearson’s correlation and *p*-value) between the input gene and the associated transcripts using the log-transformed normalized CPM values from all 79 samples. The number of transcripts associated with a node is determined by the *p*-value cutoff defined by the user (1E-10 as default).

### Databases

Xenbase (http://www.xenbase.org/, RRID:SCR_003280). Data generated by the TGCA (https://cancergenome.nih.gov/) was facilitated by the cBioPortal data visualization website [111,112].

### EctoMap application

In order to allow broad access to these data, we developed an application, EctoMap, either as full version to download and run locally (full version, Fig 11) or as light version online. Below are indicated the characteristics of each version. Given a list of genes (or a single gene for the light online version of EctoMap), the application provides different complementary information extracted from the sequencing data: (1) Ectodermal expression pattern prediction using either the average pattern or the NMF pattern, both normalized as percent of the highest expression level, for each of St. 12.5 and St. 14. In the full application, this is accompanied by the list of genes with the closest expression profile (spatial and temporal) and their respective correlation values. (2) Expression values (FPKM) for WEs throughout neurulation (St. 11–St. 19) describe the temporal dynamics of gene expression. (3) The absolute expression levels (log-2 [mean RPKM] values) and an index of ectoderm enrichment are plotted for each dissected region at St. 12.5 or St. 14. Ectoderm enrichment ranges from bright orange (value of 0) to dark green (value of 100). This enrichment is the ratio of expression in a given tissue compared to global expression in WE at the same stage. For example, a score over 90 indicates that the gene is ranked above 90% of all genes enriched in that particular tissue, whereas a rank of 10 suggests the gene is expressed elsewhere in the embryo (mesoderm, endoderm). This function is implemented in the full version; in EctoMap Lite online, WE average expression level is simply indicated by a dotted line. A heat plot of hierarchical cluster based on the Pearson or Spearman’s correlation on 79 samples, i.e., from dissected tissues and WEs at different developmental time points, is provided to gain insights into the expression profile similarity of a particular gene compared to other well-characterized genes that serve as ectodermal landmarks (e.g., NC, NB, epidermal, ventral, anterior). (5) A gene co-expression network shows the expression pattern relationships between the genes selected by the user (input nodes) and genes having strong correlations.

The application tool was built in R using the shiny app (http://shiny.rstudio.com/). The code and its documentation are deposited in Github and can be downloaded from supplementary materials (EctoMap.v1.3.zip). The link for online access of a light version of EctoMap is https://monsoro-lab-ectomap.shinyapps.io/EctoMAP/. The scripts can be adopted by any user who would like to include additional RNA-seq datasets, e.g., WEs or single tissues from wild types or gene knockdowns. The scripts can be modified and submitted for revision via Github.

## Supporting information

S1 Fig Silhouette analysis. (A, B) We have used the silhouette number (a clustering quality measure) to assess how samples cluster according to their dissected region in the space defined by the first 3 principal component analysis (PCA) components. We have compared 3 commonly used methods at stage 12.5 and stage 14: the range of log2(expression) (range), the variance (var), and the interquartile range (iqr). The silhouette number allows comparing these 3 methods and defining the appropriate number of genes to select (red line). All 3 methods gave similar silhouette profiles as a function of number of genes, with a sharp drop as genes with lower differential expression were included. We used these curves to define that between 60 and 1,200 genes should be selected for stage 12.5 and between 1,100 and 6,000 genes for stage 14. (C, D) PCA plots along component 1 (PC1) and component 2 (PC2) illustrate the influence of the number of genes selected on the quality of PCA results. (E, F) At both stages 12.5 and 14, a common threshold of 5 (red dotted line) in the range of gene expression was used to define the set of genes used in the first analyses: 1,174 genes at stage 12.5 and 1,859 genes at stage 14.
(TIFF)

S2 Fig Distribution of principal component analysis (PCA) components. Each PCA component captures a decreasing part of the variance in gene expression between samples. Significance is computed as indicated in Materials and methods, and 1% significance line is drawn. PCA component contribution to variance is plotted for stage 12.5 samples (A), stage 14 samples (B), and both stages together (C).
(TIFF)

S3 Fig Principal component analysis (PCA) identifies genes whose expression is strongly correlated with PCA components 1 and 2. As we matched PCA components 1 and 2 to dorsal-ventral (D-V) and anterior-posterior (A-P) axes, we looked for the genes best correlated to each component (S4 Table). Color code indicates dissection region identity of the sample according to Fig 1 and S1 Table. (A, B) Neural plate gene *sox2*.*1* expression is correlated to PCA component 1 at both stages, more weakly at stage 12.5 (A, correlation coefficient = −0.86) than at stage 14 (B, correlation coefficient = −0.91). (C, D) Novel gene zc4h2.l expression is even more highly correlated to PCA component 1 at stage 12.5 (C, correlation coefficient = −0.98) and stage 14 (D, correlation coefficient = −0.96). (E, F) Posterior gene *cdx2*.*s* presents a high negative correlation with PCA component 2, both at stage 12.5 (E, correlation coefficient = −0.93) and 14 (F, correlation coefficient = −0.95). (G, H) Anterior gene fzd8.s is highly positively correlated with PCA component 2, both at stage 12.5 (G, correlation coefficient = 0.91) and 14 (H, correlation coefficient = 0.88). See S11 Table for numerical data.
(TIFF)

S4 Fig Nonnegative matrix factorization (NMF)-predicted expression for genes most correlated to principal component analysis (PCA) axes 1 and 2. Dissected regions are projected along PCA components 1 and 2 in a pattern matching with embryonic dorsal-ventral (D-V) and anterior-posterior (A-P) axes, respectively. We have selected the 5 genes most correlated to these components (from list in S4 Table) and predicted their expression pattern using NMF. Patterns for the 5 genes with best positive or negative correlation are presented. This independent analysis confirms that the selected genes show clear D-V or A-P pattern restrictions and could thus be good diagnostic markers for position along each axis, respectively.
(TIFF)

S5 Fig Expression level distribution between biological replicates. (A, B) Expression level for each biological replicate is plotted for *snail2* and *sox2*, for each dissected tissue. This highlights the variability of expression that complicates the establishment of region-specific gene signatures for adjacent regions. Ec = NNE, nonneural ectoderm. See S11 Table for numerical data.
(TIFF)

S6 Fig Nonnegative matrix factorization (NMF) convergence. Convergence of NMF to a single minimum was assessed by clustering rows of the mixing matrix obtained by running NMF deconvolution with random initialization 20 times and checking that the number of tight clusters obtained equal NMF rank. This procedure shows that a single minimum is obtained up to rank 5 both at stage 12.5 (A) and stage 14 (B), although for stage 12.5, rank 4 does not lead to tight clusters. Higher ranks do not lead to a single solution, as the number of clusters recovered exceeds the rank and/or clusters are not tight (rank 6 at stage 12.5). Mixing matrices for each solution from 2 to 7 ranks are shown. Stars (*) point out clusters that are not tight.
(TIFF)

S7 Fig Gini index defines a specific gene signature for each nonnegative matrix factorization (NMF)-predicted ectoderm region. Gini enrichment index was used to define the genes most specifically enriched in 1 of the 5 tissues predicted by NMF deconvolution (S5 Table). Five genes with highest Gini index are shown for each NMF-tissue. This defined known and novel genes to characterize each ectoderm region.
(TIFF)

S8 Fig Distinction between ectoderm-enriched genes and posterior mesoderm-expressed genes by combining nonnegative matrix factorization (NMF) pattern and average expression analysis. In complement to average or NMF-predicted expression patterns, we indicate the enrichment of gene expression in ectoderm, compared to whole embryo expression. Ectoderm enrichment index allows distinguishing between genes highly and specifically expressed in the ectoderm germ layer (e.g., *sox10*, dark green, expression of which is initiated at stage 14) and genes found in the posterior neural tissue because of attached mesoderm cells (*myod*, *myf5*, yellow, low level). All intermediate situations are found, including ubiquitously expressed genes or genes enriched in 1 dissected region but not in the others. Ec = NNE, nonneural ectoderm. See S11 Table for numerical data.
(TIFF)

S9 Fig Weighted gene correlation network analysis (WGCNA). (A) Selection of the soft threshold power used to obtain the signed matrix. (B) Mean connectivity for each of the soft threshold powers. (C) Dendrogram displaying the 141 gene co-expression groups obtained by the high topological overlap using the dynamic tree cut algorithm, using a soft power threshold of 22, hybrid cut, deep split = 4. A minimum of 15 genes were required to belong to a cluster; otherwise, genes were assigned to Group #0. See S11 Table for numerical data.
(TIFF)

S10 Fig Weighted gene correlation network analysis (WGCNA) identifies homeologous gene pairs expressed with different spatial or temporal dynamics during neurulation. (A) The gene set used for WGCNA contained 2,520 gene pairs, most of which display different expression dynamics, thus falling into different WGCNA groups. (B) Global analysis of groups: groups associated with developmental processes (G51, G23, G34, and G38) tend to contain both copies of homeologous pairs, while groups associated with global cell biology retained few pairs. (C) Gene ontology (GO) terms associated with groups retaining few pairs (terms related to cell—cell relationships) or with groups retaining pairs (terms related to transcription). See S11 Table for numerical data.
(TIFF)

S11 Fig Expression dynamics of homeologous gene copies during neurulation. Expression pattern comparison between selected homeologous transcription factor pairs. The spatial and temporal expression of both copies of *pax3*, *sox10*, and *six3* highly correlate in all 79 tissue samples (R^2^ > 96, *p*-value = 0). Other genes exhibit expression level differences over different time points (*id2*, *zic1*, *six4*) or across space and time (*snai2*, *id2*). The lowest correlations observed between the displayed homeologous pairs are *snai2*.*l* and *snai2*.*s* (R^2^ = 0.60, *p*-value = 4e-09) and *zic1*.*s* and *zic1*.*l* (R^2^ = 0.65, *p*-value = 4e-11). The parentheses after the gene names correspond to the co-expression groups assigned by weighted gene correlation network analysis (WGCNA). See S11 Table for numerical data.
(TIFF)

S12 Fig Analysis of evolution of *snail2* homeologous gene pair. *Snail2*.*l* and *snail2*.*s* exhibit differential expression with asymmetrical decrease of *snail2*.*s* (S11 Fig). (A) Tree for *snail2* genes. Snail2.l, the copy with retained expression in *X*. *laevis*, is closer to *X*. *tropicalis* gene than to *X*. *laevis snail2*.*s*. (B) In *X*. *laevis*, mutations are accumulating in the low complexity regions of Snail2.s protein compared to *Snail2*.*l or X*. *tropicalis* Snail2. (C) Detail of the mutation observed on Snail2 proteins. (D) Protein alignment confirms that the mutations in Snail2.s are found on residues conserved in other vertebrates.
(TIFF)

S1 Application EctoMap and EctoMap Lite codes. (ZIP)

S2 Application Codes for generating article figures. (ZIP)

S1 Web Archive Example of EctoMap run on *bmp4* gene, showing the different features in the complete application (not interactive). (ZIP)

S1 Table Samples used for deep sequencing. The individual samples used for each stage and each area are listed and color coded to match patterns in Figs 1 and 3. The number of biological replicates for each sample is indicated.
(XLSX)

S2 Table Primers used for sample validation by quantitative PCR prior to sequencing. Sequences and references for the published primer pairs used to validate sample quality.
(XLSX)

S3 Table Ectoderm enrichment filtering. Genes expressed at a higher level in the whole embryo than in any ectoderm sample were filtered out. This would remove potential minor mesoderm contamination from neural plate and neural border posterior halves.
(XLSX)

S4 Table Genes most correlated to principal component analysis (PCA) axes 1 and 2. At each stage, PCA axes indicate which variables discriminate better each sample (dissected tissue). Genes most correlated to axes 1 and 2 are retrieved for each stage (positive and negative correlation). Genes most correlated to axis 3 at stage 14 are *msx2*, *pfkfb4*, and *sox9* (positive correlation, all expressed in the neural border) and *tll1*, *traf4*, *dag1*, and *sox21* (negative correlation).
(XLSX)

S5 Table Genes most enriched in nonnegative matrix factorization (NMF)-tissues. Gini enrichment index was used to define the genes most specifically enriched in 1 of the 5 tissues predicted by NMF deconvolution.
(XLSX)

S6 Table (A) Main weighted gene correlation network analysis (WGCNA) groups with associated gene ontology (GO) terms. (B) All GO terms with associated WGCNA groups.
(XLSX)

S7 Table (A) Genes contained into the weighted gene correlation network analysis (WGCNA) groups described in the Result section. (B) Fisher’s tests for enrichment in Wnt signaling-associated genes. (C) Genes related to neural crest and placodes: repartition into WGCNA groups. (D) Fisher’s tests for enrichment in neural crest and placode-related genes in WGCNA groups.
(XLSX)

S8 Table (A) Homeologous gene pairs distribution into weighted gene correlation network analysis (WGCNA) groups. The 2,520 homeologous pairs were selected by choosing the human gene ortholog (HUGO) gene names that had exactly 2 annotations that corresponded to the homeologous pairs. Columns (A) and (D) are colored based on whether both homeologous genes are found in a single WGCNA group (white); yellow means the genes are found in a different module. (B) List of *Xenopus laevis* gene pairs associated to 1 HUGO name.
(XLSX)

S9 Table Analysis linked to super-enhancers in melanoma. (XLSX)

S10 Table Gene list selected for illustrating the network analysis. Gene list used as an input for WGCNA clustering and to generate the co-expression network. For generating the network, a mixed list of genes was used as an input, including neural, neural crest, placodes, mesoderm, and signaling genes.
(XLSX)

S11 Table Numerical data to build each figure. (XLSX)

## Abbreviations

ANB: anterior neural border
ANF: anterior neural fold
A-P: anterior-posterior
BMP: bone morphogenetic protein
D-V: dorsal-ventral
EctoMap: ectodermal map of differential gene expression
EMT: epithelium-to-mesenchyme transition
FGF: fibroblast growth factors
GEO: Gene Expression Omnibus
GO: gene ontology
GRN: gene regulatory network
GWAS: genome-wide association study
H3K27Ac: K27-acetylated histone 3
HUGO: human gene ortholog
MAPK: mitogen-activated protein kinase
My: million years
NB: neural border
NBa: anterior neural border
NBp: posterior neural border
NC: neural crest
NMF: nonnegative matrix factorization
NNE: nonneural ectoderm
NP: neural plate
NPa: anterior neural plate
NPp: posterior neural plate
PCA: principal component analysis
PPE: preplacodal ectoderm
qPCR: quantitative PCR
RPKM: reads per kilobase per million
St.: Nieuwkoop and Faber stage
tSNE: t-distributed stochastic neighbor embedding
WE: whole embryo
WGCNA: weighted gene correlation network analysis
WNT: Wingless-Int

## References

[1] Liu JA, Cheung M. Neural crest stem cells and their potential therapeutic applications. Dev Biol. 2016;419: 199–216. doi: 10.1016/j.ydbio.2016.09.006 27640086 10.1016/j.ydbio.2016.09.006

[2] Schlosser G. Vertebrate cranial placodes as evolutionary innovations—the ancestor’s tale. Curr Top Dev Biol. 2015;111: 235–300. doi: 10.1016/bs.ctdb.2014.11.008 25662263 10.1016/bs.ctdb.2014.11.008

[3] Harland R. Neural induction. Curr Opin Genet Dev. 2000;10: 357–362. 10889069 10.1016/s0959-437x(00)00096-4

[4] Pla P, Monsoro-Burq A-H. Xenopus Embryo: Neural Induction In: Wiley John & Ltd Sons, editor. eLS. Chichester, UK: John Wiley & Sons, Ltd; 2015 pp. 1–10. http://doi.wiley.com/10.1002/9780470015902.a0000731.pub2

[5] Blitz IL, Paraiso KD, Patrushev I, Chiu WTY, Cho KWY, Gilchrist MJ. A catalog of Xenopus tropicalis transcription factors and their regional expression in the early gastrula stage embryo. Dev Biol. 2016; doi: 10.1016/j.ydbio.2016.07.002 27475627 10.1016/j.ydbio.2016.07.002 PMC5596316

[6] Pegoraro C, Figueiredo AL, Maczkowiak F, Pouponnot C, Eychène A, Monsoro-Burq AH. PFKFB4 controls embryonic patterning via Akt signalling independently of glycolysis. Nat Commun. 2015;6: 5953 doi: 10.1038/ncomms6953 25601028 10.1038/ncomms6953

[7] Junker JP, Noël ES, Guryev V, Peterson KA, Shah G, Huisken J, et al Genome-wide RNA Tomography in the zebrafish embryo. Cell. 2014;159: 662–675. doi: 10.1016/j.cell.2014.09.038 25417113 10.1016/j.cell.2014.09.038

[8] Peng G, Suo S, Chen J, Chen W, Liu C, Yu F, et al Spatial Transcriptome for the Molecular Annotation of Lineage Fates and Cell Identity in Mid-gastrula Mouse Embryo. Dev Cell. 2016;36: 681–697. doi: 10.1016/j.devcel.2016.02.020 27003939 10.1016/j.devcel.2016.02.020

[9] Combs PA, Eisen MB. Sequencing mRNA from cryo-sliced Drosophila embryos to determine genome-wide spatial patterns of gene expression. PLoS ONE. 2013;8: e71820 doi: 10.1371/journal.pone.0071820 23951250 10.1371/journal.pone.0071820 PMC3741199

[10] Riddiford N, Schlosser G. Dissecting the pre-placodal transcriptome to reveal presumptive direct targets of Six1 and Eya1 in cranial placodes. eLife. 2016;5 doi: 10.7554/eLife.17666 27576864 10.7554/eLife.17666 PMC5035141

[11] Kojima Y, Kaufman-Francis K, Studdert JB, Steiner KA, Power MD, Loebel DAF, et al The transcriptional and functional properties of mouse epiblast stem cells resemble the anterior primitive streak. Cell Stem Cell. 2014;14: 107–120. doi: 10.1016/j.stem.2013.09.014 24139757 10.1016/j.stem.2013.09.014

[12] Satija R, Farrell JA, Gennert D, Schier AF, Regev A. Spatial reconstruction of single-cell gene expression data. Nat Biotechnol. 2015;33: 495–502. doi: 10.1038/nbt.3192 25867923 10.1038/nbt.3192 PMC4430369

[13] Forouzmand E, Owens NDL, Blitz IL, Paraiso KD, Khokha MK, Gilchrist MJ, et al Developmentally regulated long non-coding RNAs in Xenopus tropicalis. Dev Biol. 2016; doi: 10.1016/j.ydbio.2016.06.016 27418388 10.1016/j.ydbio.2016.06.016 PMC5233649

[14] Yanai I, Peshkin L, Jorgensen P, Kirschner MW. Mapping gene expression in two Xenopus species: evolutionary constraints and developmental flexibility. Dev Cell. 2011;20: 483–496. doi: 10.1016/j.devcel.2011.03.015 21497761 10.1016/j.devcel.2011.03.015 PMC3118554

[15] Patthey C, Clifford H, Haerty W, Ponting CP, Shimeld SM, Begbie J. Identification of molecular signatures specific for distinct cranial sensory ganglia in the developing chick. Neural Develop. 2016;11: 3 doi: 10.1186/s13064-016-0057-y 26819088 10.1186/s13064-016-0057-y PMC4730756

[16] Session AM, Uno Y, Kwon T, Chapman JA, Toyoda A, Takahashi S, et al Genome evolution in the allotetraploid frog Xenopus laevis. Nature. 2016;538: 336–343. doi: 10.1038/nature19840 27762356 10.1038/nature19840 PMC5313049

[17] Nieuwkoop PD, Faber J. Normal table of Xenopus laevis (Daudin) : a systematical and chronological survey of the development from the fertilized egg till the end of metamorphosis. Amsterdam: North-Holland Pub. Co; 1967.

[18] de Crozé N, Maczkowiak F, Monsoro-Burq AH. Reiterative AP2a activity controls sequential steps in the neural crest gene regulatory network. Proc Natl Acad Sci U S A. 2011;108: 155–160. doi: 10.1073/pnas.1010740107 21169220 10.1073/pnas.1010740107 PMC3017139

[19] Basch ML, Bronner-Fraser M, García-Castro MI. Specification of the neural crest occurs during gastrulation and requires Pax7. Nature. 2006;441: 218–222. doi: 10.1038/nature04684 16688176 10.1038/nature04684

[20] Steventon B, Mayor R. Early neural crest induction requires an initial inhibition of Wnt signals. Dev Biol. 2012;365: 196–207. doi: 10.1016/j.ydbio.2012.02.029 22394485 10.1016/j.ydbio.2012.02.029 PMC3657187

[21] Steventon B, Mayor R, Streit A. Neural crest and placode interaction during the development of the cranial sensory system. Dev Biol. 2014;389: 28–38. doi: 10.1016/j.ydbio.2014.01.021 24491819 10.1016/j.ydbio.2014.01.021 PMC4439187

[22] Schlosser G. Early embryonic specification of vertebrate cranial placodes. Wiley Interdiscip Rev Dev Biol. 2014;3: 349–363. doi: 10.1002/wdev.142 25124756 10.1002/wdev.142

[23] Eagleson GW, Harris WA. Mapping of the presumptive brain regions in the neural plate of Xenopus laevis. J Neurobiol. 1990 4;21(3):427–40. doi: 10.1002/neu.480210305 2351962 10.1002/neu.480210305

[24] Simões-Costa M, Bronner ME. Establishing neural crest identity: a gene regulatory recipe. Dev Camb Engl. 2015;142: 242–257. doi: 10.1242/dev.105445 25564621 10.1242/dev.105445 PMC4302844

[25] Moody SA, LaMantia A-S. Transcriptional regulation of cranial sensory placode development. Curr Top Dev Biol. 2015;111: 301–350. doi: 10.1016/bs.ctdb.2014.11.009 25662264 10.1016/bs.ctdb.2014.11.009 PMC4425424

[26] Milet C, Monsoro-Burq AH. Dissection of Xenopus laevis neural crest for in vitro explant culture or in vivo transplantation. J Vis Exp JoVE. 2014; doi: 10.3791/51118 24637938 10.3791/51118 PMC4123508

[27] Theveneau E, Steventon B, Scarpa E, Garcia S, Trepat X, Streit A, et al Chase-and-run between adjacent cell populations promotes directional collective migration. Nat Cell Biol. 2013;15: 763–772. doi: 10.1038/ncb2772 23770678 10.1038/ncb2772 PMC4910871

[28] Millet S, Campbell K, Epstein DJ, Losos K, Harris E, Joyner AL. A role for Gbx2 in repression of Otx2 and positioning the mid/hindbrain organizer. Nature. 1999;401: 161–164. doi: 10.1038/43664 10490024 10.1038/43664

[29] Diez del Corral R, Storey KG. Opposing FGF and retinoid pathways: a signalling switch that controls differentiation and patterning onset in the extending vertebrate body axis. BioEssays News Rev Mol Cell Dev Biol. 2004;26: 857–869. doi: 10.1002/bies.20080 15273988 10.1002/bies.20080

[30] Simeone A, Acampora D, Gulisano M, Stornaiuolo A, Boncinelli E. Nested expression domains of four homeobox genes in developing rostral brain. Nature. 1992;358: 687–690. doi: 10.1038/358687a0 1353865 10.1038/358687a0

[31] Kablar B, Vignali R, Menotti L, Pannese M, Andreazzoli M, Polo C, et al Xotx genes in the developing brain of Xenopus laevis. Mech Dev. 1996;55: 145–158. 8861095 10.1016/0925-4773(96)00497-2

[32] Vignali R, Colombetti S, Lupo G, Zhang W, Stachel S, Harland RM, et al Xotx5b, a new member of the Otx gene family, may be involved in anterior and eye development in Xenopus laevis. Mech Dev. 2000;96: 3–13. doi: 10.1016/S0925-4773(00)00367-1 10940620 10.1016/s0925-4773(00)00367-1

[33] Matsuo-Takasaki M, Lim JH, Beanan MJ, Sato SM, Sargent TD. Cloning and expression of a novel zinc finger gene, Fez, transcribed in the forebrain of Xenopus and mouse embryos. Mech Dev. 2000;93: 201–204. 10781957 10.1016/s0925-4773(00)00264-1

[34] Hashimoto H, Yabe T, Hirata T, Shimizu T, Bae Y, Yamanaka Y, et al Expression of the zinc finger gene fez-like in zebrafish forebrain. Mech Dev. 2000;97: 191–195. 11025224 10.1016/s0925-4773(00)00418-4

[35] Bae C-J, Jeong J, Saint-Jeannet J-P. A novel function for Egr4 in posterior hindbrain development. Sci Rep. 2015;5: 7750 doi: 10.1038/srep07750 25583070 10.1038/srep07750 PMC4291570

[36] Monsoro-Burq A-H, Wang E, Harland R. Msx1 and Pax3 Cooperate to Mediate FGF8 and WNT Signals during Xenopus Neural Crest Induction. Dev Cell. 2005;8: 167–178. doi: 10.1016/j.devcel.2004.12.017 15691759 10.1016/j.devcel.2004.12.017

[37] Spokony RF, Aoki Y, Saint-Germain N, Magner-Fink E, Saint-Jeannet J-P. The transcription factor Sox9 is required for cranial neural crest development in Xenopus. Dev Camb Engl. 2002;129: 421–432. 10.1242/dev.129.2.421 11807034

[38] Lee DD, Seung HS. Learning the parts of objects by non-negative matrix factorization. Nature. 1999;401: 788–791. doi: 10.1038/44565 10548103 10.1038/44565

[39] Brunet J-P, Tamayo P, Golub TR, Mesirov JP. Metagenes and molecular pattern discovery using matrix factorization. Proc Natl Acad Sci U S A. 2004;101: 4164–4169. doi: 10.1073/pnas.0308531101 15016911 10.1073/pnas.0308531101 PMC384712

[40] Sharpe CR, Fritz A, De Robertis EM, Gurdon JB. A homeobox-containing marker of posterior neural differentiation shows the importance of predetermination in neural induction. Cell. 1987;50: 749–758. 2441873 10.1016/0092-8674(87)90333-3

[41] Innocenzi A, Latella L, Messina G, Simonatto M, Marullo F, Berghella L, et al An evolutionarily acquired genotoxic response discriminates MyoD from Myf5, and differentially regulates hypaxial and epaxial myogenesis. EMBO Rep. 2011;12: 164–171. doi: 10.1038/embor.2010.195 21212806 10.1038/embor.2010.195 PMC3049428

[42] Gini C. Variabilità e mutabilità. Repr Mem Metodol Stat Ed Pizetti E Salvemini T Rome Libr Eredi Virgilio Veschi. 1912;1.

[43] Niehrs C, Pollet N. Synexpression groups in eukaryotes. Nature. 1999;402: 483–487. doi: 10.1038/990025 10591207 10.1038/990025

[44] Dequéant M-L, Fagegaltier D, Hu Y, Spirohn K, Simcox A, Hannon GJ, et al Discovery of progenitor cell signatures by time-series synexpression analysis during Drosophila embryonic cell immortalization. Proc Natl Acad Sci U S A. 2015;112: 12974–12979. doi: 10.1073/pnas.1517729112 26438832 10.1073/pnas.1517729112 PMC4620889

[45] Langfelder P, Horvath S. WGCNA: an R package for weighted correlation network analysis. BMC Bioinformatics. 2008;9: 559 doi: 10.1186/1471-2105-9-559 19114008 10.1186/1471-2105-9-559 PMC2631488

[46] Kaur A, Webster MR, Weeraratna AT. In the Wnt-er of life: Wnt signalling in melanoma and ageing. Br J Cancer. 2016;115: 1273–1279. doi: 10.1038/bjc.2016.332 27764844 10.1038/bjc.2016.332 PMC5129830

[47] Kaufman CK, Mosimann C, Fan ZP, Yang S, Thomas AJ, Ablain J, et al A zebrafish melanoma model reveals emergence of neural crest identity during melanoma initiation. Science. 2016;351: aad2197–aad2197. doi: 10.1126/science.aad2197 26823433 10.1126/science.aad2197 PMC4868069

[48] Milet C, Monsoro-Burq AH. Neural crest induction at the neural plate border in vertebrates. Dev Biol. 2012;366: 22–33. doi: 10.1016/j.ydbio.2012.01.013 22305800 10.1016/j.ydbio.2012.01.013

[49] Kjolby RAS, Harland RM. Genome-wide identification of Wnt/β-catenin transcriptional targets during Xenopus gastrulation. Dev Biol. 2016; doi: 10.1016/j.ydbio.2016.03.021 27091726 10.1016/j.ydbio.2016.03.021 PMC6288011

[50] Nakamura Y, de Paiva Alves E, Veenstra GJC, Hoppler S. Tissue- and stage-specific Wnt target gene expression is controlled subsequent to β-catenin recruitment to cis-regulatory modules. Dev Camb Engl. 2016;143: 1914–1925. doi: 10.1242/dev.131664 27068107 10.1242/dev.131664 PMC4920159

[51] Ogino H, Ochi H, Reza HM, Yasuda K. Transcription factors involved in lens development from the preplacodal ectoderm. Dev Biol. 2012;363: 333–347. doi: 10.1016/j.ydbio.2012.01.006 22269169 10.1016/j.ydbio.2012.01.006

[52] Groves AK, LaBonne C. Setting appropriate boundaries: Fate, patterning and competence at the neural plate border. Dev Biol. 2014;389: 2–12. doi: 10.1016/j.ydbio.2013.11.027 24321819 10.1016/j.ydbio.2013.11.027 PMC3972267

[53] Betancur P, Bronner-Fraser M, Sauka-Spengler T. Assembling neural crest regulatory circuits into a gene regulatory network. Annu Rev Cell Dev Biol. 2010;26: 581–603. doi: 10.1146/annurev.cellbio.042308.113245 19575671 10.1146/annurev.cellbio.042308.113245 PMC4040144

[54] Simões-Costa M, Tan-Cabugao J, Antoshechkin I, Sauka-Spengler T, Bronner ME. Transcriptome analysis reveals novel players in the cranial neural crest gene regulatory network. Genome Res. 2014;24: 281–290. doi: 10.1101/gr.161182.113 24389048 10.1101/gr.161182.113 PMC3912418

[55] Fischer T, Guimera J, Wurst W, Prakash N. Distinct but redundant expression of the Frizzled Wnt receptor genes at signaling centers of the developing mouse brain. Neuroscience. 2007;147: 693–711. doi: 10.1016/j.neuroscience.2007.04.060 17582687 10.1016/j.neuroscience.2007.04.060

[56] Viczian AS, Bang AG, Harris WA, Zuber ME. Expression of Xenopus laevis Lhx2 during eye development and evidence for divergent expression among vertebrates. Dev Dyn Off Publ Am Assoc Anat. 2006;235: 1133–1141. doi: 10.1002/dvdy.20708 16470628 10.1002/dvdy.20708

[57] Andreazzoli M, Gestri G, Angeloni D, Menna E, Barsacchi G. Role of Xrx1 in Xenopus eye and anterior brain development. Dev Camb Engl. 1999;126: 2451–2460. 10.1242/dev.126.11.2451 10226004

[58] Zhang S, Li J, Lea R, Vleminckx K, Amaya E. Fezf2 promotes neuronal differentiation through localised activation of Wnt/β-catenin signalling during forebrain development. Dev Camb Engl. 2014;141: 4794–4805. doi: 10.1242/dev.115691 25468942 10.1242/dev.115691 PMC4299278

[59] Watanabe M, Yasuoka Y, Mawaribuchi S, Kuretani A, Ito M, Kondo M, et al Conservatism and variability of gene expression profiles among homeologous transcription factors in Xenopus laevis. Dev Biol. 2016 10 31 pii: S0012-1606(16)30053-7. doi: 10.1016/j.ydbio.2016.09.017 27810169 10.1016/j.ydbio.2016.09.017

[60] Michiue T, Yamamoto T, Yasuoka Y, Goto T, Ikeda T, Nagura K, et al High variability of expression profiles of homeologous genes for Wnt, Hh, Notch, and Hippo signaling pathways in Xenopus laevis. Dev Biol. 2017 1 12 pii: S0012-1606(16)30056-2. doi: 10.1016/j.ydbio.2016.12.006 28089430 10.1016/j.ydbio.2016.12.006

[61] Lan X, Pritchard JK. Coregulation of tandem duplicate genes slows evolution of subfunctionalization in mammals. Science. 2016 5 20;352(6288):1009–13. doi: 10.1126/science.aad8411 27199432 10.1126/science.aad8411 PMC5182070

[62] McGrew LL, Hoppler S, Moon RT. Wnt and FGF pathways cooperatively pattern anteroposterior neural ectoderm in Xenopus. Mech Dev. 1997;69: 105–114. 9486534 10.1016/s0925-4773(97)00160-3

[63] Blumberg B, Bolado J, Moreno TA, Kintner C, Evans RM, Papalopulu N. An essential role for retinoid signaling in anteroposterior neural patterning. Dev Camb Engl. 1997;124: 373–379. 10.1242/dev.124.2.373 9053313

[64] Hirschhorn JN, Daly MJ. Genome-wide association studies for common diseases and complex traits. Nat Rev Genet. 2005;6: 95–108. doi: 10.1038/nrg1521 15716906 10.1038/nrg1521

[65] Maguire LH, Thomas AR, Goldstein AM. Tumors of the neural crest: Common themes in development and cancer. Dev Dyn Off Publ Am Assoc Anat. 2015;244: 311–322. doi: 10.1002/dvdy.24226 25382669 10.1002/dvdy.24226

[66] Hnisz D, Abraham BJ, Lee TI, Lau A, Saint-André V, Sigova AA, et al Super-enhancers in the control of cell identity and disease. Cell. 2013;155: 934–947. doi: 10.1016/j.cell.2013.09.053 24119843 10.1016/j.cell.2013.09.053 PMC3841062

[67] Whyte WA, Orlando DA, Hnisz D, Abraham BJ, Lin CY, Kagey MH, et al Master transcription factors and mediator establish super-enhancers at key cell identity genes. Cell. 2013;153: 307–319. doi: 10.1016/j.cell.2013.03.035 23582322 10.1016/j.cell.2013.03.035 PMC3653129

[68] Hendrix MJC, Seftor EA, Seftor REB, Kasemeier-Kulesa J, Kulesa PM, Postovit L-M. Reprogramming metastatic tumour cells with embryonic microenvironments. Nat Rev Cancer. 2007;7: 246–255. doi: 10.1038/nrc2108 17384580 10.1038/nrc2108

[69] Kuphal S, Palm HG, Poser I, Bosserhoff AK. Snail-regulated genes in malignant melanoma. Melanoma Res. 2005;15: 305–313. 16034310 10.1097/00008390-200508000-00012

[70] Asnaghi L, Gezgin G, Tripathy A, Handa JT, Merbs SL, van der Velden PA, et al EMT-associated factors promote invasive properties of uveal melanoma cells. Mol Vis. 2015;21: 919–929. 26321866 PMC4548792

[71] Shakhova O, Zingg D, Schaefer SM, Hari L, Civenni G, Blunschi J, et al Sox10 promotes the formation and maintenance of giant congenital naevi and melanoma. Nat Cell Biol. 2012;14: 882–890. doi: 10.1038/ncb2535 22772081 10.1038/ncb2535

[72] Shirley SH, Greene VR, Duncan LM, Torres Cabala CA, Grimm EA, Kusewitt DF. Slug expression during melanoma progression. Am J Pathol. 2012;180: 2479–2489. doi: 10.1016/j.ajpath.2012.02.014 22503751 10.1016/j.ajpath.2012.02.014 PMC3378849

[73] Lu M, Krauss RS. N-cadherin ligation, but not Sonic hedgehog binding, initiates Cdo-dependent p38alpha/beta MAPK signaling in skeletal myoblasts. Proc Natl Acad Sci U S A. 2010;107: 4212–4217. doi: 10.1073/pnas.0908883107 20160094 10.1073/pnas.0908883107 PMC2840122

[74] Delloye-Bourgeois C, Rama N, Brito J, Le Douarin N, Mehlen P. Sonic Hedgehog promotes the survival of neural crest cells by limiting apoptosis induced by the dependence receptor CDON during branchial arch development. Biochem Biophys Res Commun. 2014;452: 655–660. doi: 10.1016/j.bbrc.2014.08.134 25193697 10.1016/j.bbrc.2014.08.134

[75] Powell DR, Williams JS, Hernandez-Lagunas L, Salcedo E, O’Brien JH, Artinger KB. Cdon promotes neural crest migration by regulating N-cadherin localization. Dev Biol. 2015;407: 289–299. doi: 10.1016/j.ydbio.2015.07.025 26256768 10.1016/j.ydbio.2015.07.025 PMC4663112

[76] Kiecker C, Niehrs C. A morphogen gradient of Wnt/beta-catenin signalling regulates anteroposterior neural patterning in Xenopus. Dev Camb Engl. 2001;128: 4189–4201. 10.1242/dev.128.21.4189 11684656

[77] Kiecker C, Lumsden A. The role of organizers in patterning the nervous system. Annu Rev Neurosci. 2012;35: 347–367. doi: 10.1146/annurev-neuro-062111-150543 22462542 10.1146/annurev-neuro-062111-150543

[78] Silbereis JC, Pochareddy S, Zhu Y, Li M, Sestan N. The Cellular and Molecular Landscapes of the Developing Human Central Nervous System. Neuron. 2016;89: 248–268. doi: 10.1016/j.neuron.2015.12.008 26796689 10.1016/j.neuron.2015.12.008 PMC4959909

[79] Ding Y, Colozza G, Zhang K, Moriyama Y, Ploper D, Sosa EA, et al Genome-wide analysis of dorsal and ventral transcriptomes of the Xenopus laevis gastrula. Dev Biol. 2016 3 23 pii: S0012-1606(15)30329-8. doi: 10.1016/j.ydbio.2016.02.032 27016259 10.1016/j.ydbio.2016.02.032 PMC5033668

[80] Sennett R, Wang Z, Rezza A, Grisanti L, Roitershtein N, Sicchio C, et al An Integrated Transcriptome Atlas of Embryonic Hair Follicle Progenitors, Their Niche, and the Developing Skin. Dev Cell. 2015 9 14;34(5):577–91. doi: 10.1016/j.devcel.2015.06.023 26256211 10.1016/j.devcel.2015.06.023 PMC4573840

[81] Simoes-Costa M, Bronner ME. Insights into neural crest development and evolution from genomic analysis. Genome Res. 2013 7;23(7):1069–80. doi: 10.1101/gr.157586.113 23817048 10.1101/gr.157586.113 PMC3698500

[82] Simoes-Costa M, Bronner ME. Reprogramming of avian neural crest axial identity and cell fate. Science. 2016 6 24;352(6293):1570–3. doi: 10.1126/science.aaf2729 27339986 10.1126/science.aaf2729 PMC5100669

[83] Rabadan MA, Usieto S, Lavarino C, Marti E. Identification of a putative transcriptome signature common to neuroblastoma and neural crest cells. Dev Neurobiol. 2013 11;73(11):815–27. doi: 10.1002/dneu.22099 Epub 2013 Sep 17. 23776185 10.1002/dneu.22099

[84] Plouhinec J-L, Roche DD, Pegoraro C, Figueiredo AL, Maczkowiak F, Brunet LJ, et al Pax3 and Zic1 trigger the early neural crest gene regulatory network by the direct activation of multiple key neural crest specifiers. Dev Biol. 2014;386: 461–472. doi: 10.1016/j.ydbio.2013.12.010 24360906 10.1016/j.ydbio.2013.12.010 PMC3962137

[85] Bae C-J, Park B-Y, Lee Y-H, Tobias JW, Hong C-S, Saint-Jeannet J-P. Identification of Pax3 and Zic1 targets in the developing neural crest. Dev Biol. 2014;386: 473–483. doi: 10.1016/j.ydbio.2013.12.011 24360908 10.1016/j.ydbio.2013.12.011 PMC3933997

[86] Yan B, Neilson KM, Ranganathan R, Maynard T, Streit A, Moody SA. Microarray identification of novel genes downstream of Six1, a critical factor in cranial placode, somite, and kidney development. Dev Dyn Off Publ Am Assoc Anat. 2015;244: 181–210. doi: 10.1002/dvdy.24229 25403746 10.1002/dvdy.24229 PMC4428348

[87] Thélie A, Desiderio S, Hanotel J, Quigley I, Van Driessche B, Rodari A, et al Prdm12 specifies V1 interneurons through cross-repressive interactions with Dbx1 and Nkx6 genes in Xenopus. Dev Camb Engl. 2015;142: 3416–3428. doi: 10.1242/dev.121871 26443638 10.1242/dev.121871 PMC4631751

[88] De Robertis EM, Morita EA, Cho KW. Gradient fields and homeobox genes. Dev Camb Engl. 1991;112: 669–678. 10.1242/dev.112.3.669 1682124

[89] De Robertis EM, Kuroda H. Dorsal-ventral patterning and neural induction in Xenopus embryos. Annu Rev Cell Dev Biol. 2004;20: 285–308. doi: 10.1146/annurev.cellbio.20.011403.154124 15473842 10.1146/annurev.cellbio.20.011403.154124 PMC2280069

[90] Reversade B, De Robertis EM. Regulation of ADMP and BMP2/4/7 at opposite embryonic poles generates a self-regulating morphogenetic field. Cell. 2005;123: 1147–1160. doi: 10.1016/j.cell.2005.08.047 16360041 10.1016/j.cell.2005.08.047 PMC2292129

[91] Wills AE, Choi VM, Bennett MJ, Khokha MK, Harland RM. BMP antagonists and FGF signaling contribute to different domains of the neural plate in Xenopus. Dev Biol. 2010;337: 335–350. doi: 10.1016/j.ydbio.2009.11.008 19913009 10.1016/j.ydbio.2009.11.008 PMC2812634

[92] Nikitina N, Sauka-Spengler T, Bronner-Fraser M. Dissecting early regulatory relationships in the lamprey neural crest gene network. Proc Natl Acad Sci U S A. 2008;105: 20083–20088. doi: 10.1073/pnas.0806009105 19104059 10.1073/pnas.0806009105 PMC2629288

[93] Medina L, Brox A, Legaz I, García-López M, Puelles L. Expression patterns of developmental regulatory genes show comparable divisions in the telencephalon of Xenopus and mouse: insights into the evolution of the forebrain. Brain Res Bull. 2005;66: 297–302. doi: 10.1016/j.brainresbull.2005.02.003 16144605 10.1016/j.brainresbull.2005.02.003

[94] Hellsten U, Harland RM, Gilchrist MJ, Hendrix D, Jurka J, Kapitonov V, et al The genome of the Western clawed frog Xenopus tropicalis. Science. 2010;328: 633–636. doi: 10.1126/science.1183670 20431018 10.1126/science.1183670 PMC2994648

[95] Simoes-Costa M, Bronner ME. Reprogramming of avian neural crest axial identity and cell fate. Science. 2016;352: 1570–1573. doi: 10.1126/science.aaf2729 27339986 10.1126/science.aaf2729 PMC5100669

[96] Sive HL, Grainger RM, Harland RM. Early development of Xenopus laevis: a laboratory manual. CSHL Press; 2000.

[97] Monsoro-Burq AH. A rapid protocol for whole-mount in situ hybridization on Xenopus embryos. CSH Protoc. 2007;2007: pdb.prot4809. 10.1101/pdb.prot4809 21357147

[98] Grammer TC, Liu KJ, Mariani FV, Harland RM. Use of large-scale expression cloning screens in the Xenopus laevis tadpole to identify gene function. Dev Biol. 2000;228: 197–210. doi: 10.1006/dbio.2000.9945 11112324 10.1006/dbio.2000.9945

[99] Mizuseki K, Kishi M, Shiota K, Nakanishi S, Sasai Y. SoxD: an essential mediator of induction of anterior neural tissues in Xenopus embryos. Neuron. 1998;21: 77–85. 9697853 10.1016/s0896-6273(00)80516-4

[100] Mir A, Kofron M, Zorn AM, Bajzer M, Haque M, Heasman J, et al FoxI1e activates ectoderm formation and controls cell position in the Xenopus blastula. Dev Camb Engl. 2007;134: 779–788. doi: 10.1242/dev.02768 17229765 10.1242/dev.02768

[101] Trapnell C, Pachter L, Salzberg SL. TopHat: discovering splice junctions with RNA-Seq. Bioinforma Oxf Engl. 2009;25: 1105–1111. doi: 10.1093/bioinformatics/btp120 19289445 10.1093/bioinformatics/btp120 PMC2672628

[102] Trapnell C, Roberts A, Goff L, Pertea G, Kim D, Kelley DR, et al Differential gene and transcript expression analysis of RNA-seq experiments with TopHat and Cufflinks. Nat Protoc. 2012;7: 562–578. doi: 10.1038/nprot.2012.016 22383036 10.1038/nprot.2012.016 PMC3334321

[103] Quinlan AR, Hall IM. BEDTools: a flexible suite of utilities for comparing genomic features. Bioinforma Oxf Engl. 2010;26: 841–842. doi: 10.1093/bioinformatics/btq033 20110278 10.1093/bioinformatics/btq033 PMC2832824

[104] Anders S, Pyl PT, Huber W. HTSeq—a Python framework to work with high-throughput sequencing data. Bioinforma Oxf Engl. 2015;31: 166–169. doi: 10.1093/bioinformatics/btu638 25260700 10.1093/bioinformatics/btu638 PMC4287950

[105] Robinson MD, Oshlack A. A scaling normalization method for differential expression analysis of RNA-seq data. Genome Biol. 2010;11: R25 doi: 10.1186/gb-2010-11-3-r25 20196867 10.1186/gb-2010-11-3-r25 PMC2864565

[106] Robinson MD, McCarthy DJ, Smyth GK. edgeR: a Bioconductor package for differential expression analysis of digital gene expression data. Bioinforma Oxf Engl. 2010;26: 139–140. doi: 10.1093/bioinformatics/btp616 19910308 10.1093/bioinformatics/btp616 PMC2796818

[107] Ritchie ME, Phipson B, Wu D, Hu Y, Law CW, Shi W, et al limma powers differential expression analyses for RNA-sequencing and microarray studies. Nucleic Acids Res. 2015;43: e47 doi: 10.1093/nar/gkv007 25605792 10.1093/nar/gkv007 PMC4402510

[108] Edgar R, Domrachev M, Lash AE. Gene Expression Omnibus: NCBI gene expression and hybridization array data repository. Nucleic Acids Res. 2002 1 1;30(1):207–10. 11752295 10.1093/nar/30.1.207 PMC99122

[109] Gaujoux R, Seoighe C. A flexible R package for nonnegative matrix factorization. BMC Bioinformatics. 2010;11: 367 doi: 10.1186/1471-2105-11-367 20598126 10.1186/1471-2105-11-367 PMC2912887

[110] Yu G, Wang LG, Han Y, He QY. clusterProfiler: an R package for comparing biological themes among gene clusters. OMICS. 2012 5;16(5):284–7. doi: 10.1089/omi.2011.0118 22455463 10.1089/omi.2011.0118 PMC3339379

[111] Gao J, Aksoy BA, Dogrusoz U, Dresdner G, Gross B, Sumer SO, et al Integrative analysis of complex cancer genomics and clinical profiles using the cBioPortal. Sci Signal. 2013;6: pl1 doi: 10.1126/scisignal.2004088 23550210 10.1126/scisignal.2004088 PMC4160307

[112] Cerami E, Gao J, Dogrusoz U, Gross BE, Sumer SO, Aksoy BA, et al The cBio cancer genomics portal: an open platform for exploring multidimensional cancer genomics data. Cancer Discov. 2012;2: 401–404. doi: 10.1158/2159-8290.CD-12-0095 22588877 10.1158/2159-8290.CD-12-0095 PMC3956037

