# Supplementary figures and images for: A molecular atlas of the developing ectoderm defines neural, neural crest, placode, and nonneural progenitor identity in vertebrates
Source: PLoS Biol. 2017 Oct 19;15(10):e2004045. doi: 10.1371/journal.pbio.2004045 (PMC5663519; doi:10.1371/journal.pbio.2004045)

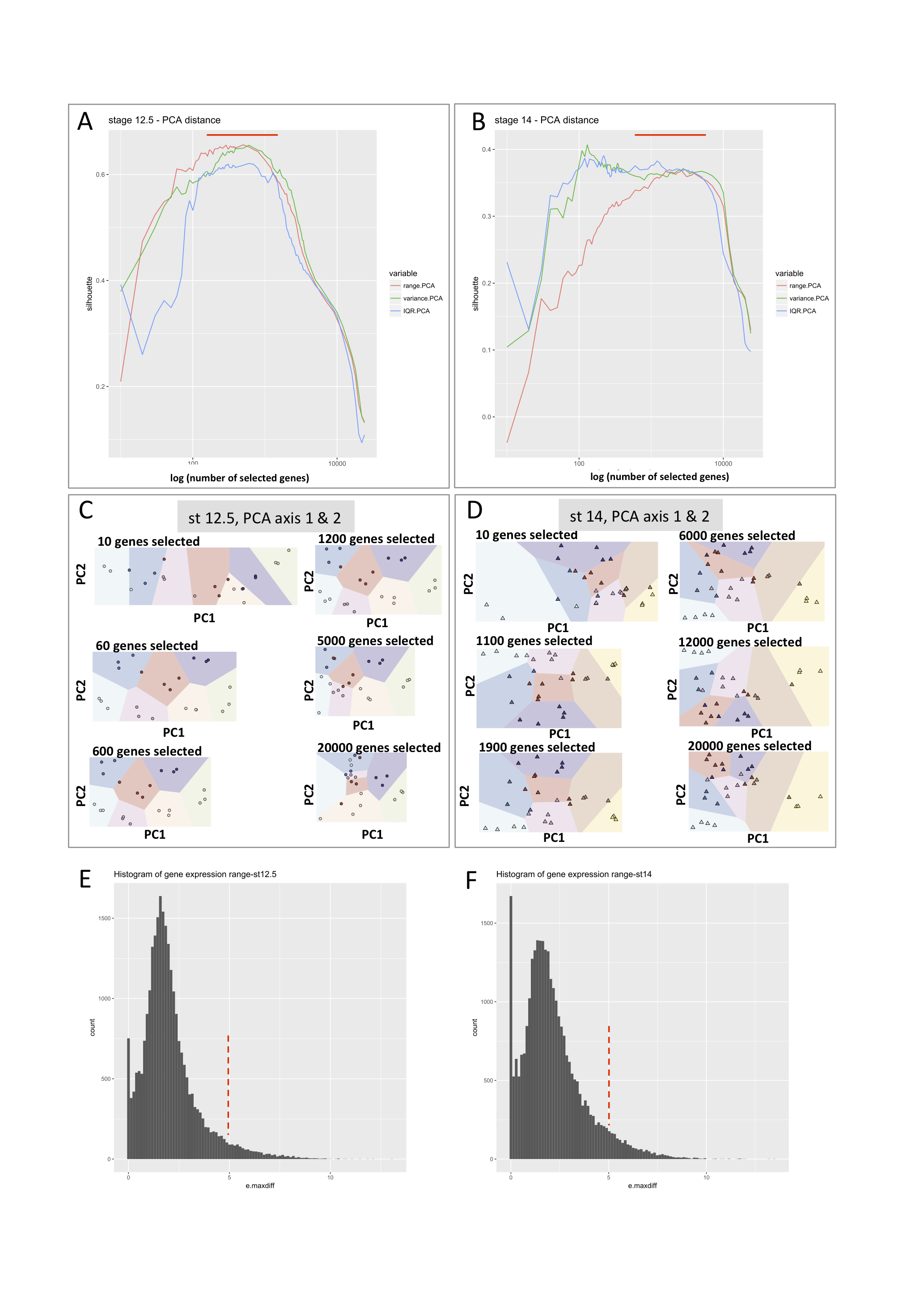

Supplement: S1 Fig — (A, B) We have used the silhouette number (a clustering quality measure) to assess how samples cluster according to their dissected region in the space defined by the first 3 principal component analysis (PCA) components. We have compared 3 commonly used methods at stage 12.5 and stage 14: the range of log2(expression) (range), the variance (var), and the interquartile range (iqr). The silhouette number allows comparing these 3 methods and defining the appropriate number of genes to select (red line). All 3 methods gave similar silhouette profiles as a function of number of genes, with a sharp drop as genes with lower differential expression were included. We used these curves to define that between 60 and 1,200 genes should be selected for stage 12.5 and between 1,100 and 6,000 genes for stage 14. (C, D) PCA plots along component 1 (PC1) and component 2 (PC2) illustrate the influence of the number of genes selected on the quality of PCA results. (E, F) At both stages 12.5 and 14, a common threshold of 5 (red dotted line) in the range of gene expression was used to define the set of genes used in the first analyses: 1,174 genes at stage 12.5 and 1,859 genes at stage 14. (TIFF) [file pbio.2004045.s001.tiff]

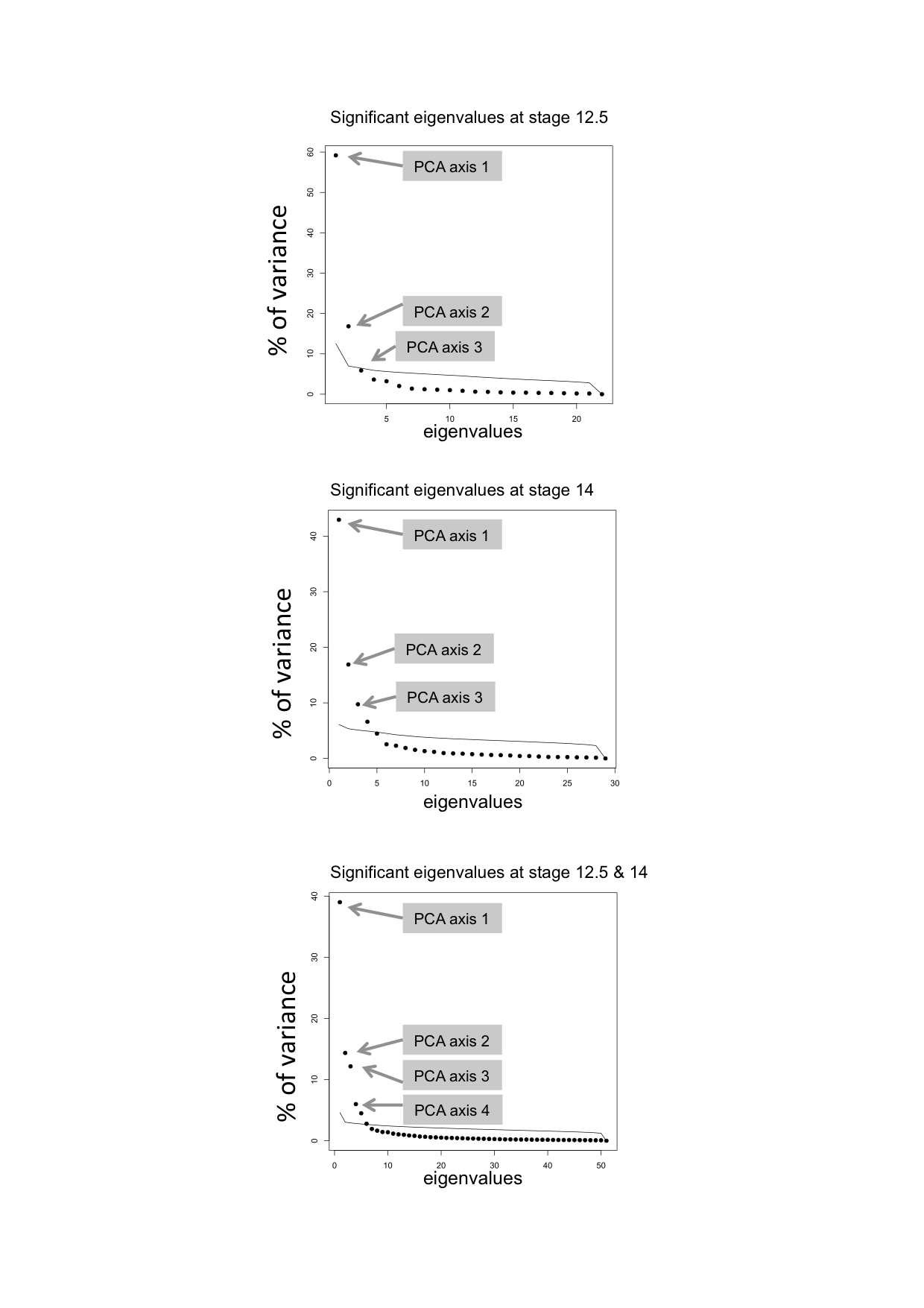

Supplement: S2 Fig — Each PCA component captures a decreasing part of the variance in gene expression between samples. Significance is computed as indicated in Materials and methods, and 1% significance line is drawn. PCA component contribution to variance is plotted for stage 12.5 samples (A), stage 14 samples (B), and both stages together (C). (TIFF) [file pbio.2004045.s002.tiff]

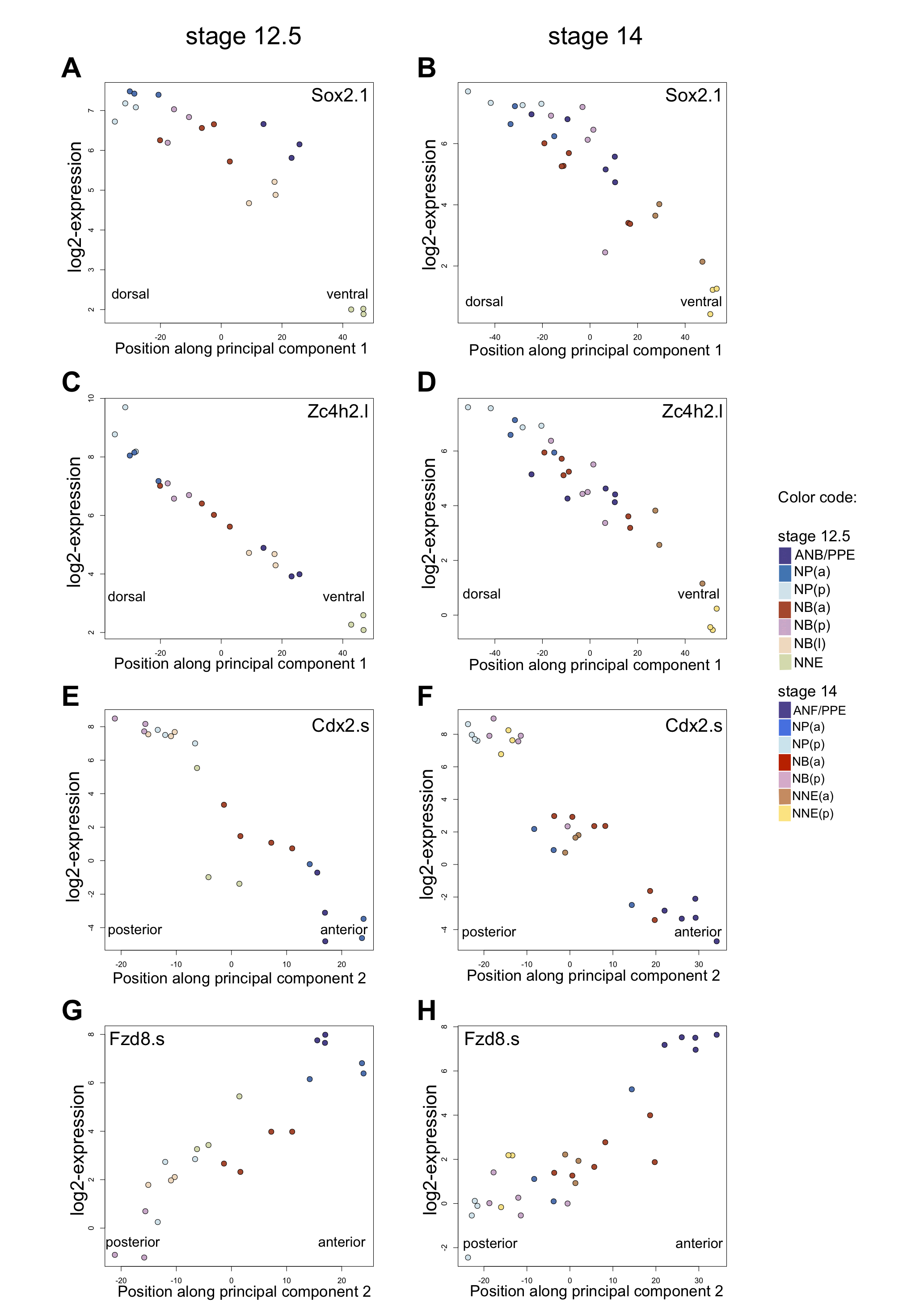

Supplement: S3 Fig — As we matched PCA components 1 and 2 to dorsal-ventral (D-V) and anterior-posterior (A-P) axes, we looked for the genes best correlated to each component (S4 Table). Color code indicates dissection region identity of the sample according to Fig 1 and S1 Table. (A, B) Neural plate gene sox2.1 expression is correlated to PCA component 1 at both stages, more weakly at stage 12.5 (A, correlation coefficient = −0.86) than at stage 14 (B, correlation coefficient = −0.91). (C, D) Novel gene zc4h2.l expression is even more highly correlated to PCA component 1 at stage 12.5 (C, correlation coefficient = −0.98) and stage 14 (D, correlation coefficient = −0.96). (E, F) Posterior gene cdx2.s presents a high negative correlation with PCA component 2, both at stage 12.5 (E, correlation coefficient = −0.93) and 14 (F, correlation coefficient = −0.95). (G, H) Anterior gene fzd8.s is highly positively correlated with PCA component 2, both at stage 12.5 (G, correlation coefficient = 0.91) and 14 (H, correlation coefficient = 0.88). See S11 Table for numerical data. (TIFF) [file pbio.2004045.s003.tiff]

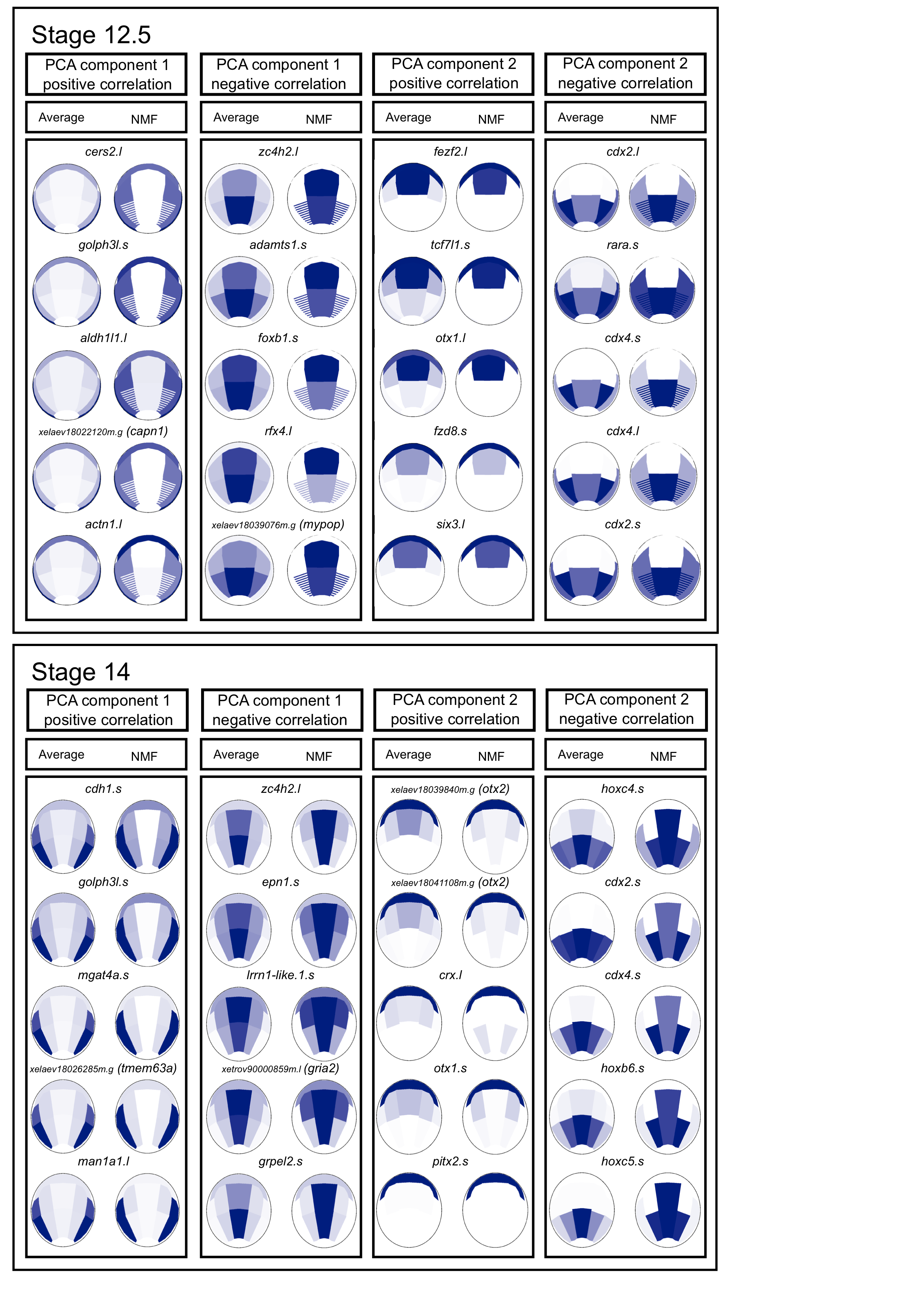

Supplement: S4 Fig — Dissected regions are projected along PCA components 1 and 2 in a pattern matching with embryonic dorsal-ventral (D-V) and anterior-posterior (A-P) axes, respectively. We have selected the 5 genes most correlated to these components (from list in S4 Table) and predicted their expression pattern using NMF. Patterns for the 5 genes with best positive or negative correlation are presented. This independent analysis confirms that the selected genes show clear D-V or A-P pattern restrictions and could thus be good diagnostic markers for position along each axis, respectively. (TIFF) [file pbio.2004045.s004.tiff]

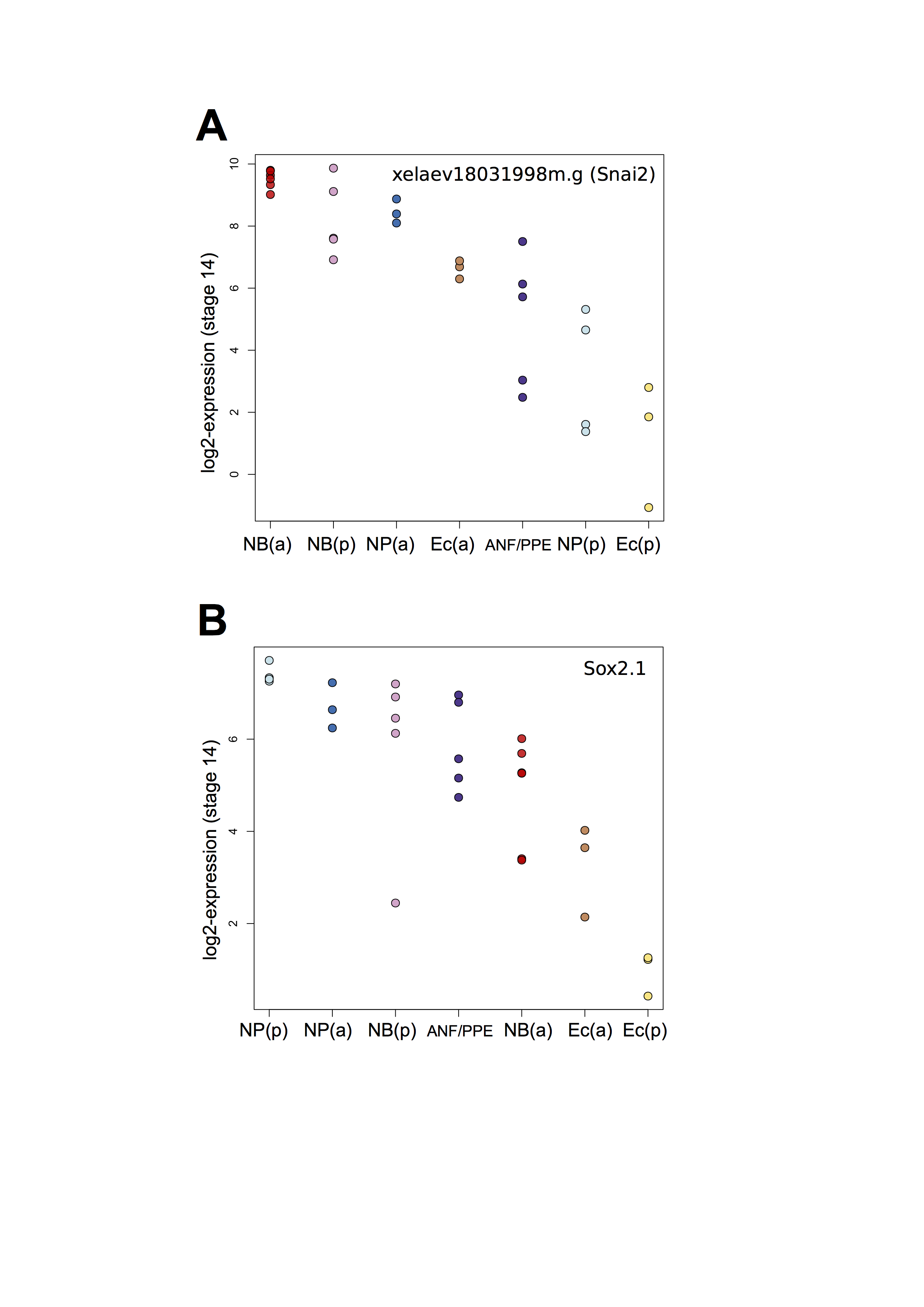

Supplement: S5 Fig — (A, B) Expression level for each biological replicate is plotted for snail2 and sox2, for each dissected tissue. This highlights the variability of expression that complicates the establishment of region-specific gene signatures for adjacent regions. Ec = NNE, nonneural ectoderm. See S11 Table for numerical data. (TIFF) [file pbio.2004045.s005.tiff]

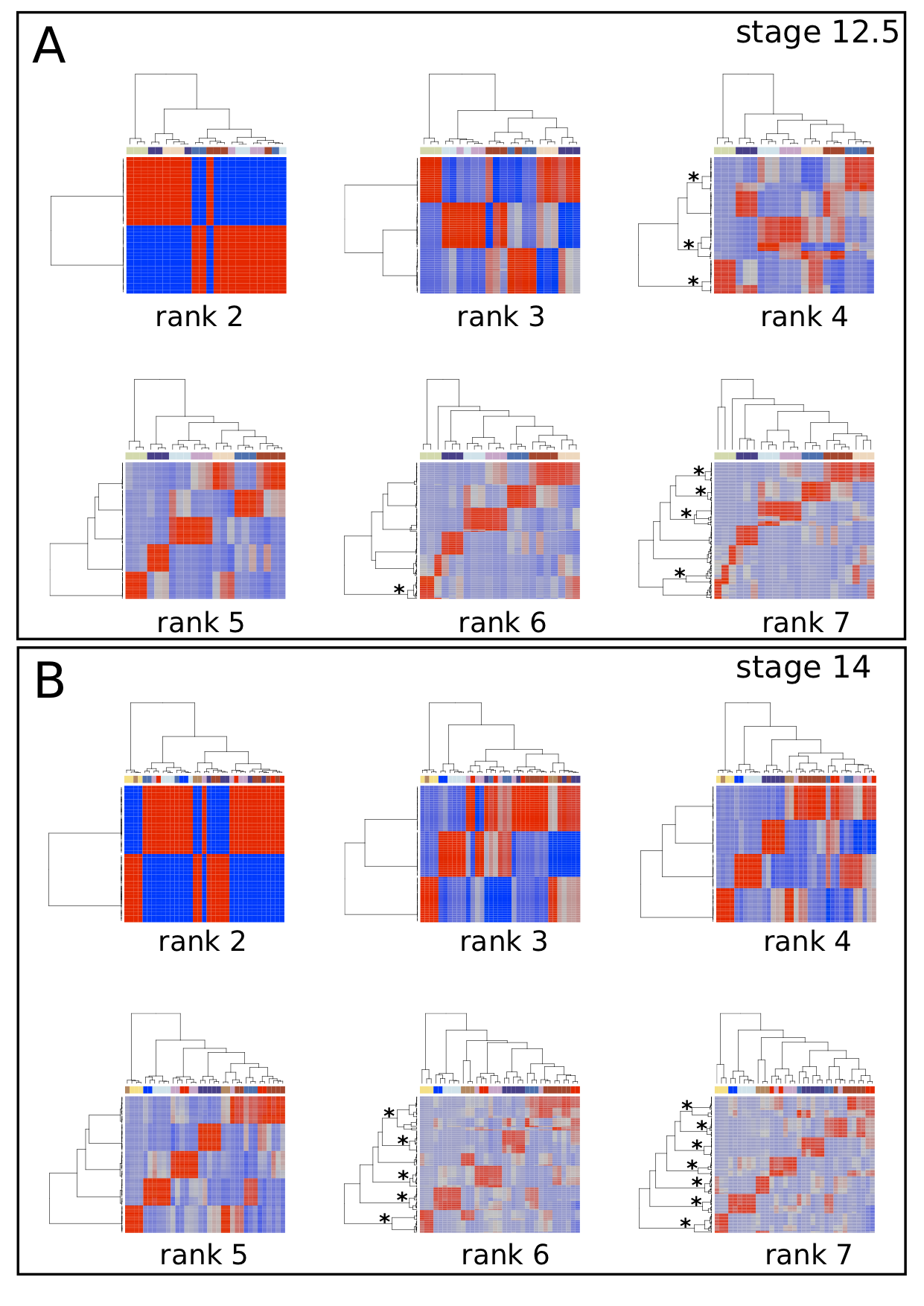

Supplement: S6 Fig — Convergence of NMF to a single minimum was assessed by clustering rows of the mixing matrix obtained by running NMF deconvolution with random initialization 20 times and checking that the number of tight clusters obtained equal NMF rank. This procedure shows that a single minimum is obtained up to rank 5 both at stage 12.5 (A) and stage 14 (B), although for stage 12.5, rank 4 does not lead to tight clusters. Higher ranks do not lead to a single solution, as the number of clusters recovered exceeds the rank and/or clusters are not tight (rank 6 at stage 12.5). Mixing matrices for each solution from 2 to 7 ranks are shown. Stars (*) point out clusters that are not tight. (TIFF) [file pbio.2004045.s006.tiff]

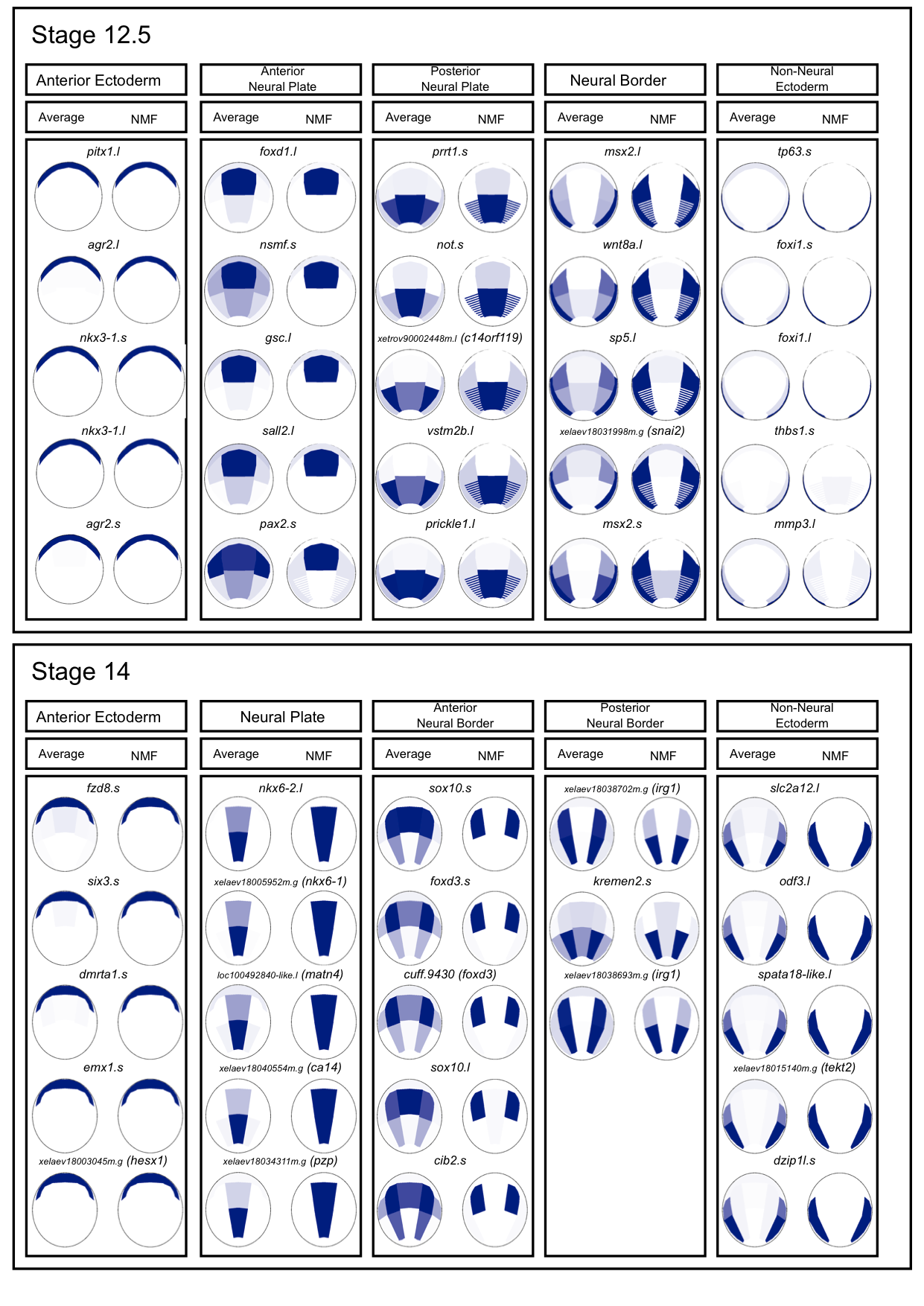

Supplement: S7 Fig — Gini enrichment index was used to define the genes most specifically enriched in 1 of the 5 tissues predicted by NMF deconvolution (S5 Table). Five genes with highest Gini index are shown for each NMF-tissue. This defined known and novel genes to characterize each ectoderm region. (TIFF) [file pbio.2004045.s007.tiff]

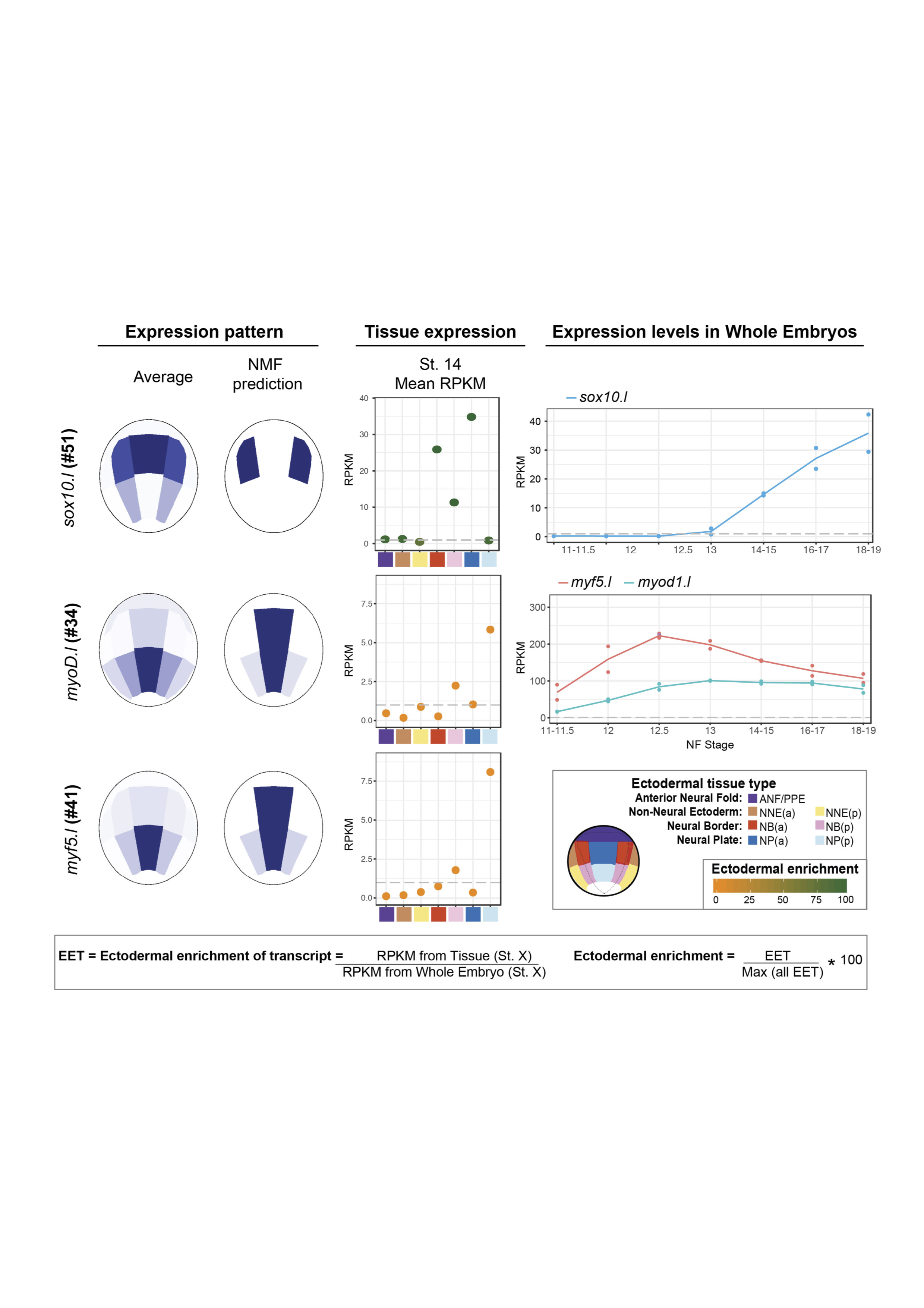

Supplement: S8 Fig — In complement to average or NMF-predicted expression patterns, we indicate the enrichment of gene expression in ectoderm, compared to whole embryo expression. Ectoderm enrichment index allows distinguishing between genes highly and specifically expressed in the ectoderm germ layer (e.g., sox10, dark green, expression of which is initiated at stage 14) and genes found in the posterior neural tissue because of attached mesoderm cells (myod, myf5, yellow, low level). All intermediate situations are found, including ubiquitously expressed genes or genes enriched in 1 dissected region but not in the others. Ec = NNE, nonneural ectoderm. See S11 Table for numerical data. (TIFF) [file pbio.2004045.s008.tiff]

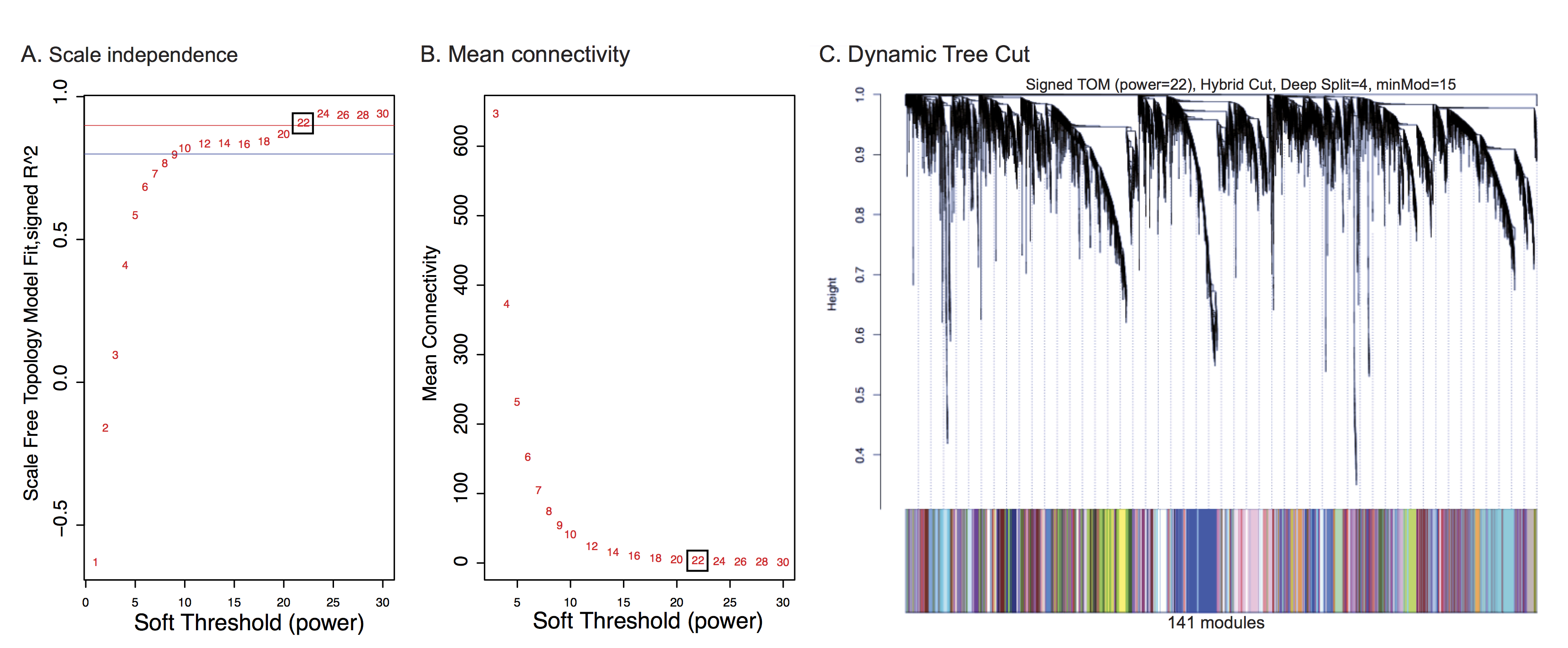

Supplement: S9 Fig — (A) Selection of the soft threshold power used to obtain the signed matrix. (B) Mean connectivity for each of the soft threshold powers. (C) Dendrogram displaying the 141 gene co-expression groups obtained by the high topological overlap using the dynamic tree cut algorithm, using a soft power threshold of 22, hybrid cut, deep split = 4. A minimum of 15 genes were required to belong to a cluster; otherwise, genes were assigned to Group #0. See S11 Table for numerical data. (TIFF) [file pbio.2004045.s009.tiff]

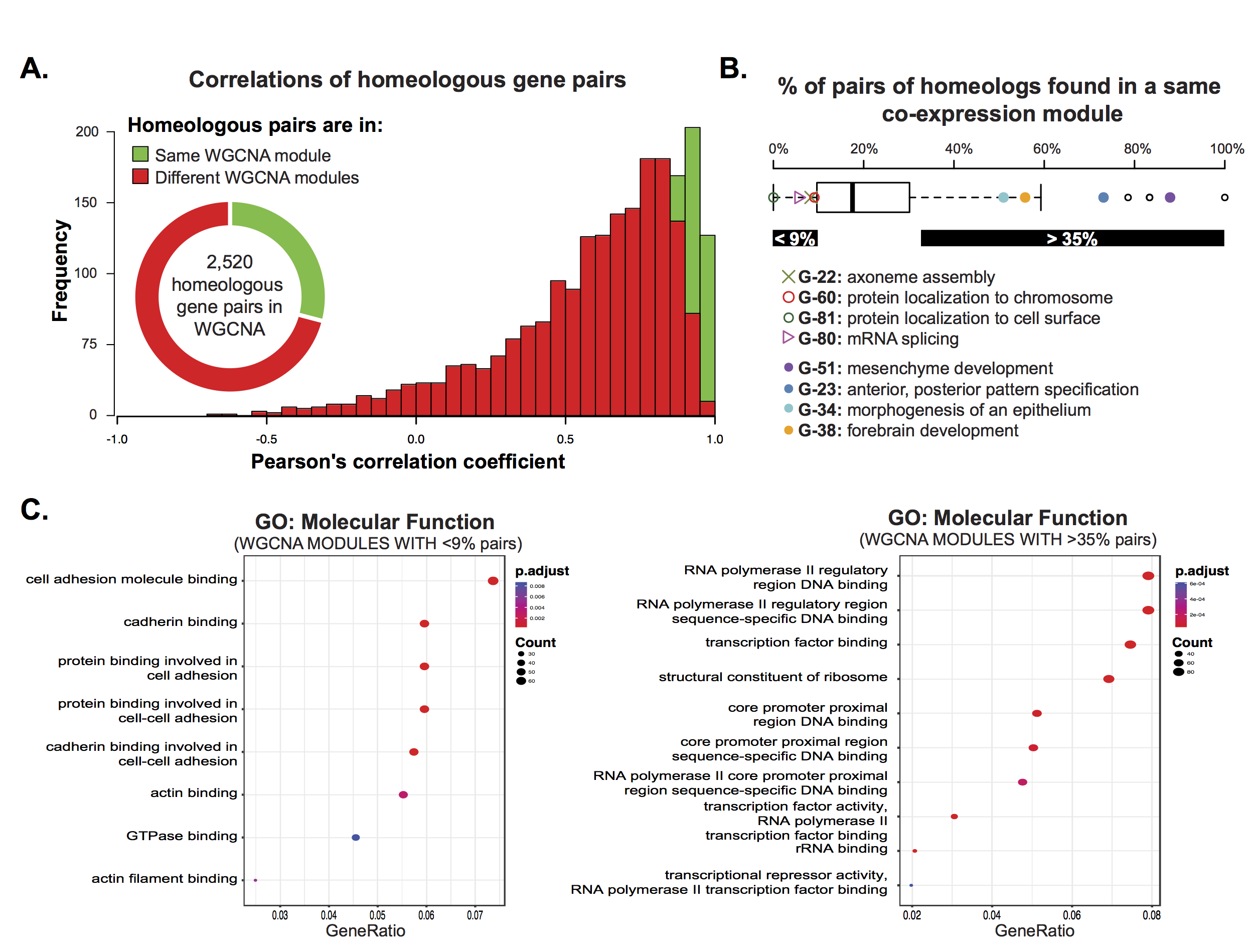

Supplement: S10 Fig — (A) The gene set used for WGCNA contained 2,520 gene pairs, most of which display different expression dynamics, thus falling into different WGCNA groups. (B) Global analysis of groups: groups associated with developmental processes (G51, G23, G34, and G38) tend to contain both copies of homeologous pairs, while groups associated with global cell biology retained few pairs. (C) Gene ontology (GO) terms associated with groups retaining few pairs (terms related to cell—cell relationships) or with groups retaining pairs (terms related to transcription). See S11 Table for numerical data. (TIFF) [file pbio.2004045.s010.tiff]

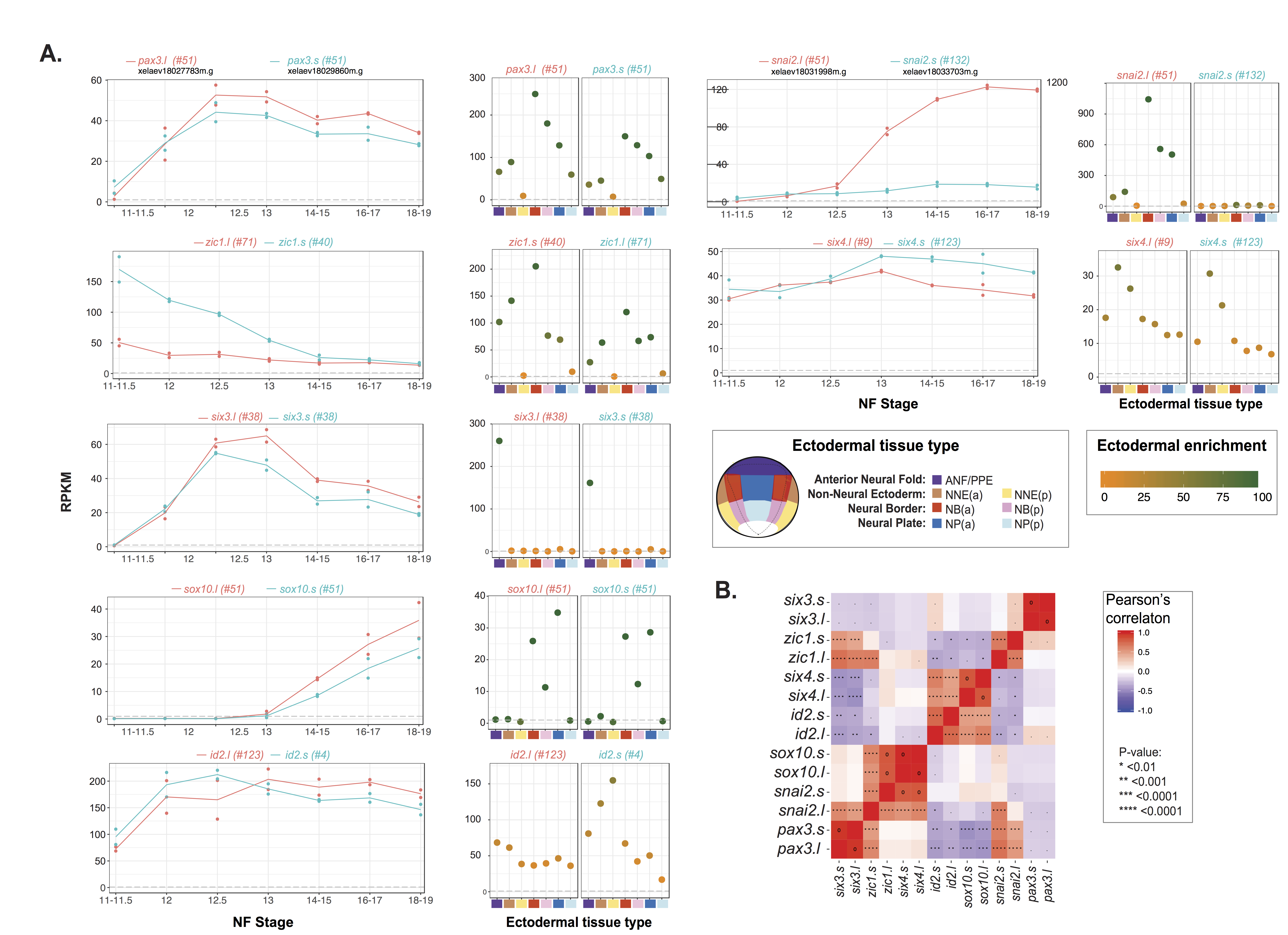

Supplement: S11 Fig — Expression pattern comparison between selected homeologous transcription factor pairs. The spatial and temporal expression of both copies of pax3, sox10, and six3 highly correlate in all 79 tissue samples (R2 > 96, p-value = 0). Other genes exhibit expression level differences over different time points (id2, zic1, six4) or across space and time (snai2, id2). The lowest correlations observed between the displayed homeologous pairs are snai2.l and snai2.s (R2 = 0.60, p-value = 4e-09) and zic1.s and zic1.l (R2 = 0.65, p-value = 4e-11). The parentheses after the gene names correspond to the co-expression groups assigned by weighted gene correlation network analysis (WGCNA). See S11 Table for numerical data. (TIFF) [file pbio.2004045.s011.tiff]

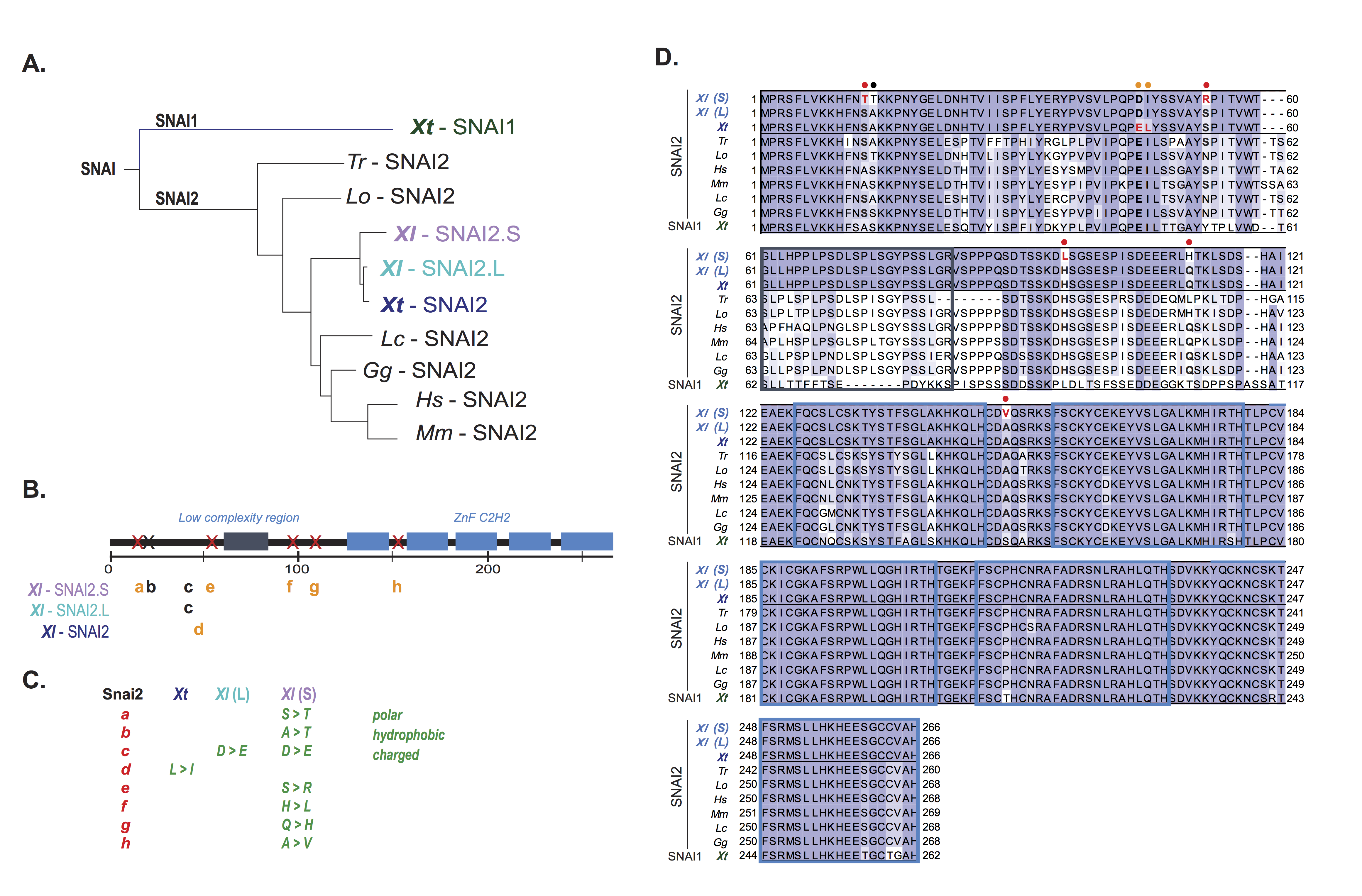

Supplement: S12 Fig — Snail2.l and snail2.s exhibit differential expression with asymmetrical decrease of snail2.s (S11 Fig). (A) Tree for snail2 genes. Snail2.l, the copy with retained expression in X. laevis, is closer to X. tropicalis gene than to X. laevis snail2.s. (B) In X. laevis, mutations are accumulating in the low complexity regions of Snail2.s protein compared to Snail2.l or X. tropicalis Snail2. (C) Detail of the mutation observed on Snail2 proteins. (D) Protein alignment confirms that the mutations in Snail2.s are found on residues conserved in other vertebrates. (TIFF) [file pbio.2004045.s012.tiff]

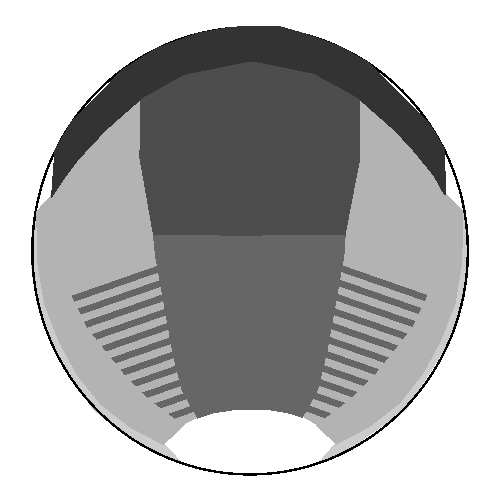

Supplement: S1 Application — (ZIP) [file pbio.2004045.s013.zip › EctoMAP_1.3/data/NMF_drawing_st12_lr.png]

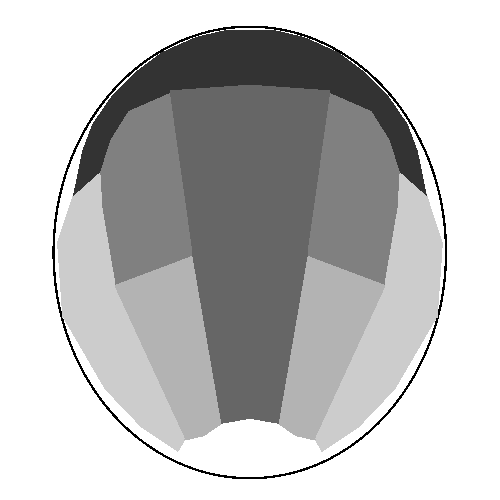

Supplement: S1 Application — (ZIP) [file pbio.2004045.s013.zip › EctoMAP_1.3/data/NMF_drawing_st14_lr.png]

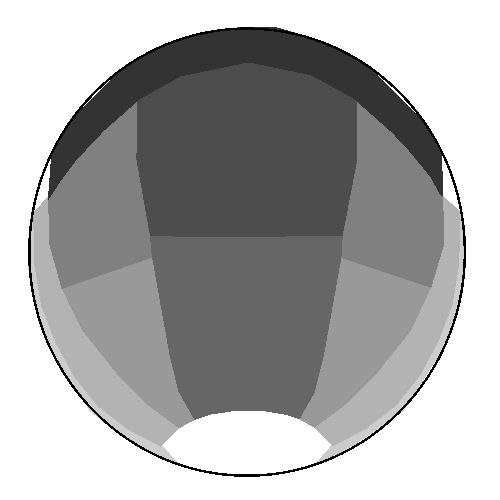

Supplement: S1 Application — (ZIP) [file pbio.2004045.s013.zip › EctoMAP_1.3/data/st12.5-average-500.png]

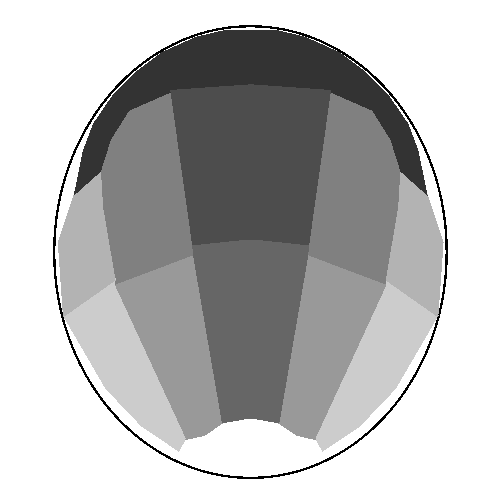

Supplement: S1 Application — (ZIP) [file pbio.2004045.s013.zip › EctoMAP_1.3/data/st14-average-500.png]

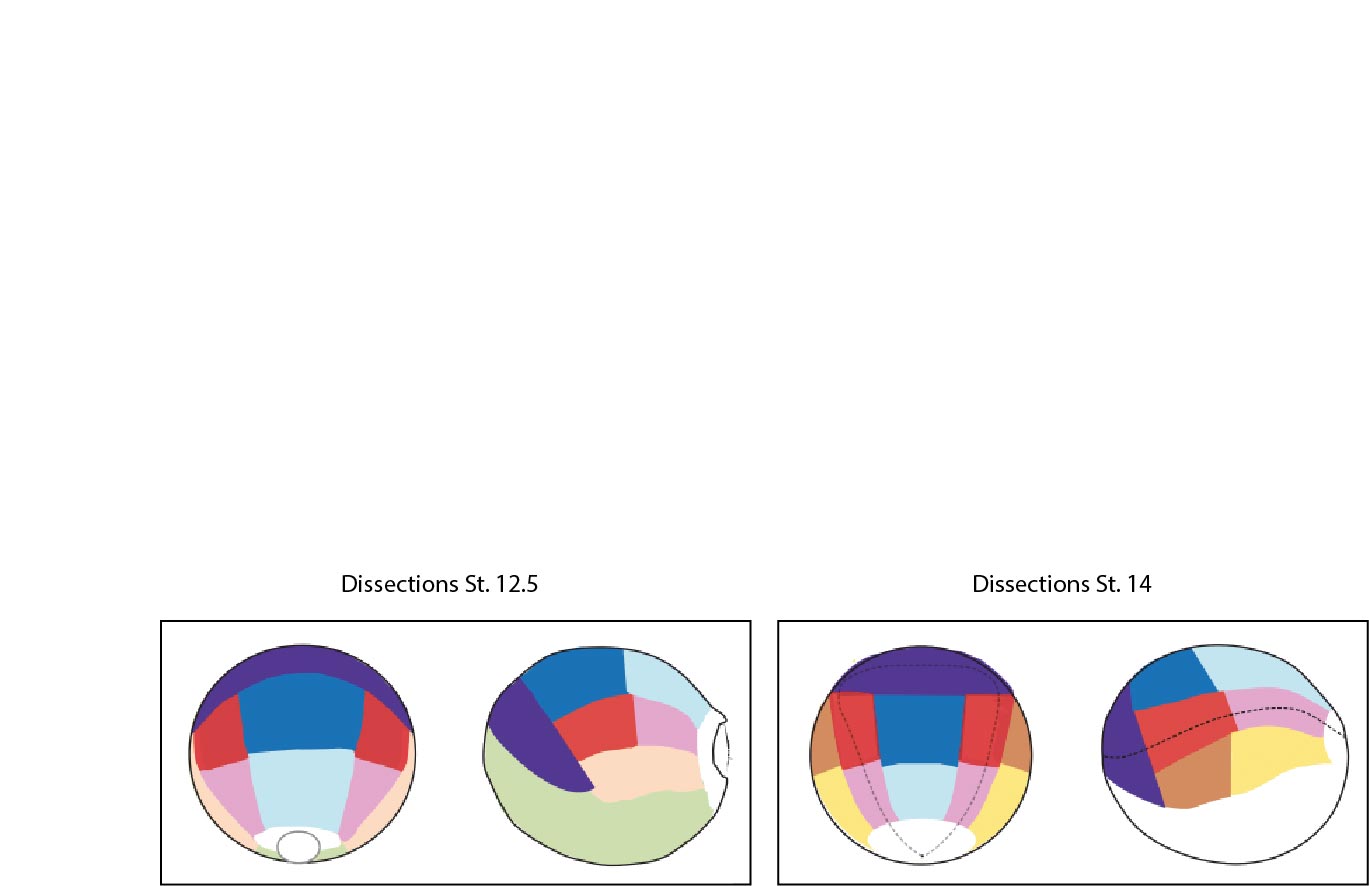

Supplement: S1 Application — (ZIP) [file pbio.2004045.s013.zip › EctoMAP_1.3/www/Disection_DiagramAH.jpg]

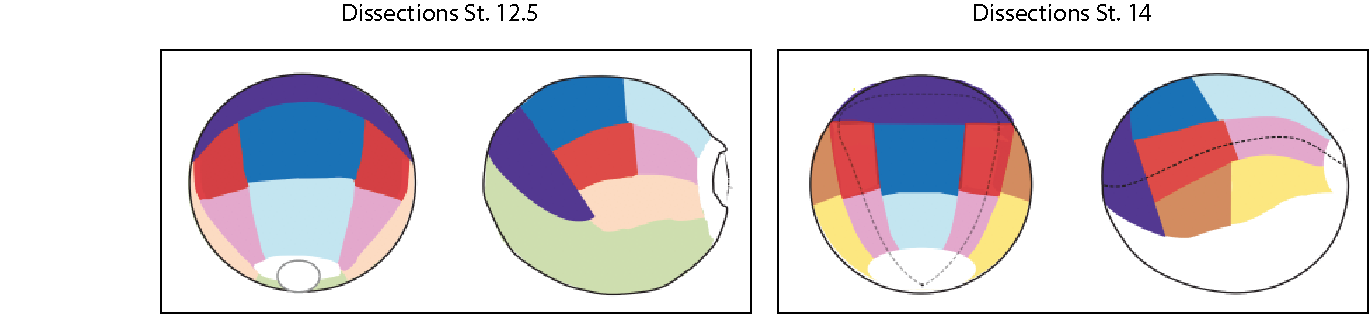

Supplement: S1 Application — (ZIP) [file pbio.2004045.s013.zip › EctoMAP_1.3/www/Disection_DiagramAH.png]

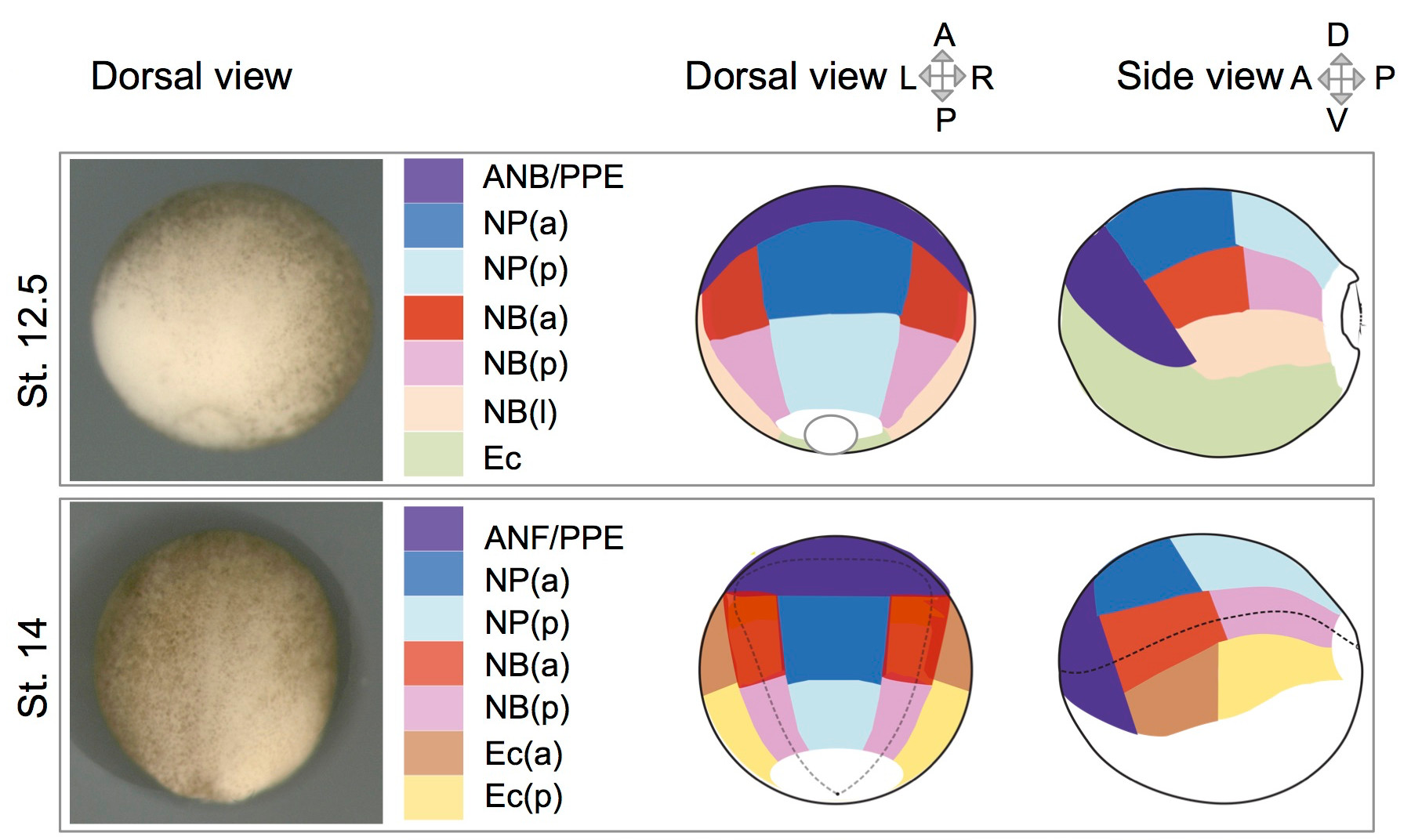

Supplement: S1 Application — (ZIP) [file pbio.2004045.s013.zip › EctoMAP_1.3/www/Dissection.jpg]

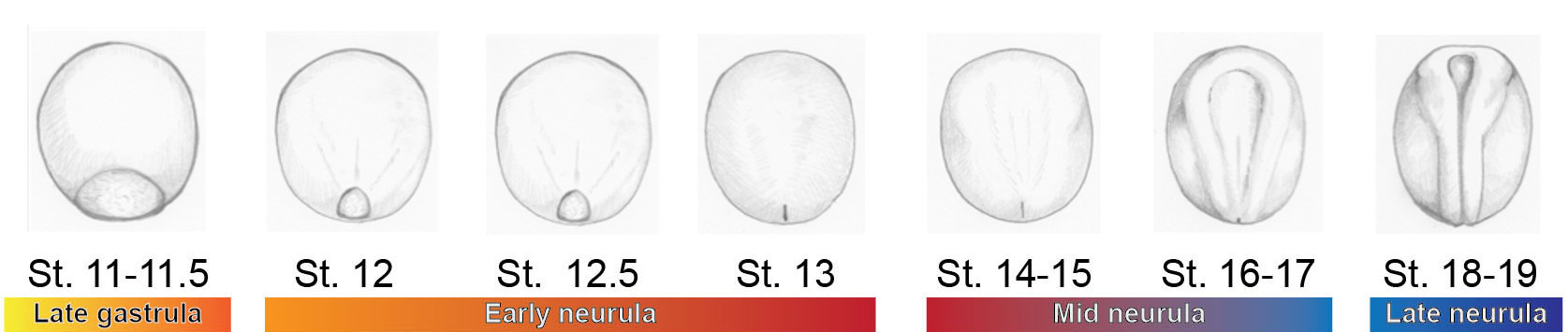

Supplement: S1 Application — (ZIP) [file pbio.2004045.s013.zip › EctoMAP_1.3/www/Temporal_expression.jpg]

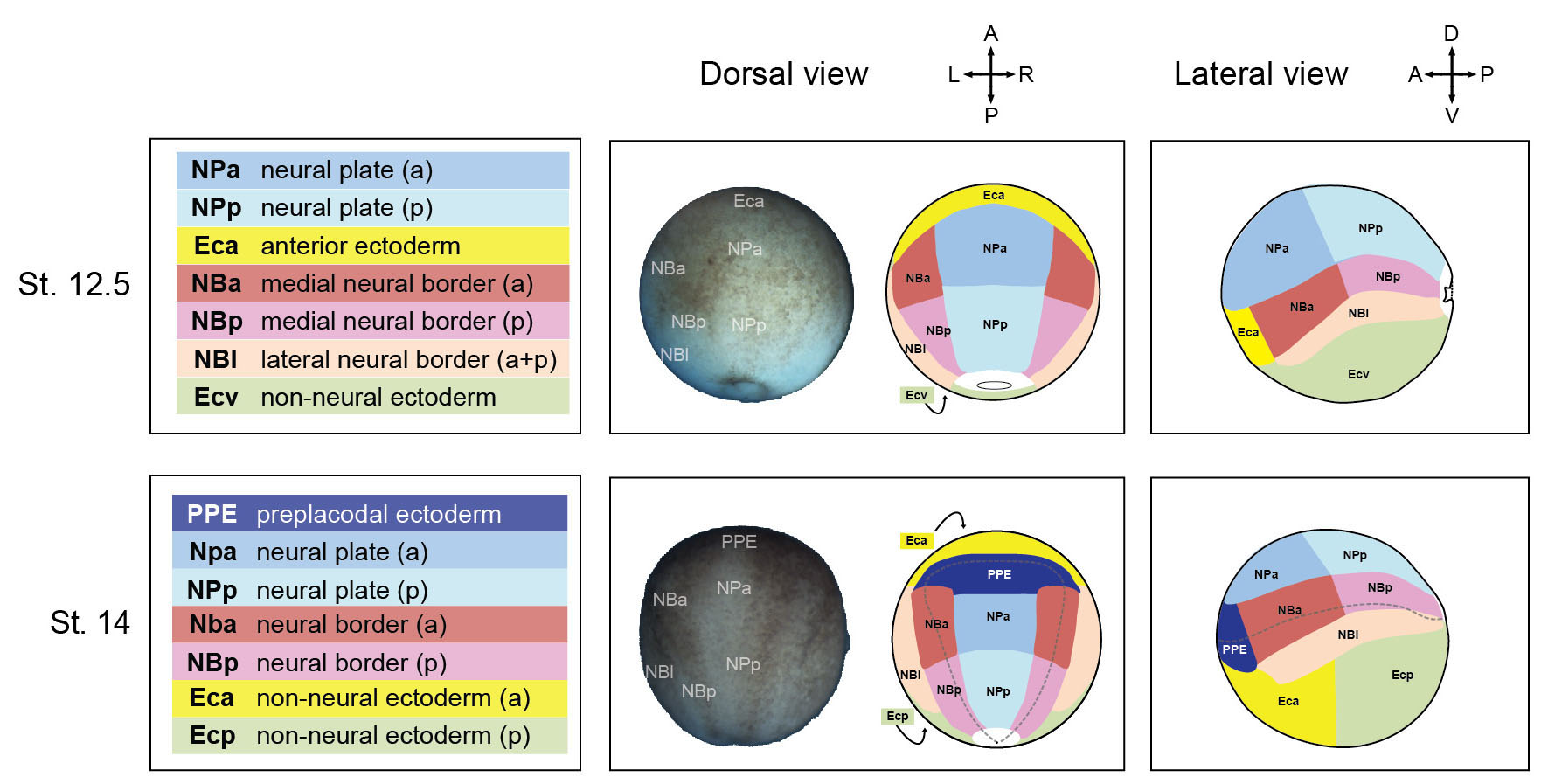

Supplement: S1 Application — (ZIP) [file pbio.2004045.s013.zip › EctoMAP_1.3/www/Tissue_dissection.jpg]

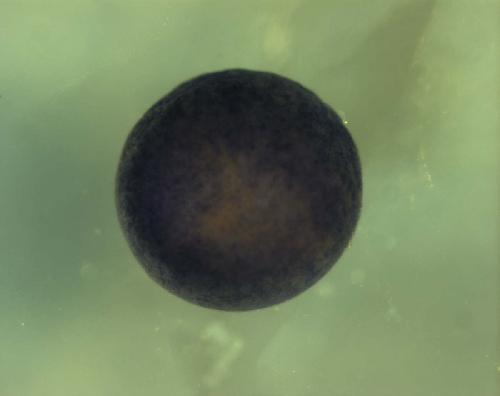

Supplement: S1 Web Archive — (ZIP) [file pbio.2004045.s015.zip › EctoMAP_example-bmp4/EctoMap_tool-bmp4_files/XB-IMG-13466.jpg]

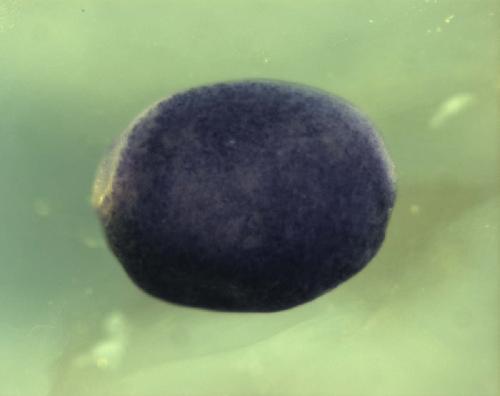

Supplement: S1 Web Archive — (ZIP) [file pbio.2004045.s015.zip › EctoMAP_example-bmp4/EctoMap_tool-bmp4_files/XB-IMG-13471.jpg]
