# Supplementary material for: A molecular atlas of the developing ectoderm defines neural, neural crest, placode, and nonneural progenitor identity in vertebrates
Source: PLoS Biol. 2017 Oct 19;15(10):e2004045. doi: 10.1371/journal.pbio.2004045 (PMC5663519; doi:10.1371/journal.pbio.2004045)
Supplement: S1 Web Archive — (ZIP) [file pbio.2004045.s015.zip › EctoMAP_example-bmp4/EctoMap_tool-bmp4.html]

EctoMap tool


## 

# **EctoMap v1.3**

# Explore the spatiotemporal expression program in the ectoderm of *Xenopus laevis* embryos.

  
  

### **Brief introduction**

EctoMap allows exploring spatial gene expression in the developing neurula ectoderm, in the frog
*X. laevis*
based on the transcriptome of dissected regions of the ectoderm
(tab 1: Gene expression in whole embryos during neurulation; tab 2:
prediction of spatial gene expression in the ectoderm of early and
mid-neurulas; tab 3: average expression in each dissected region with an
index for enrichment of gene expression in the ectoderm).

EctoMap also provides tools to study gene synexpression during
neurulation (tab2: list of correlated genes using WGCNA; tab4:
WGCNA-based network of gene synexpression). For details, see Plouhinec,
Medina et al., (in revision).

  

#### **Navigation**

This window has two main panels, the left one for input queries,
and the right one for query results, displayed as four main tabs, tab 2
containing 3 sub-tabs:

1. Time series Plot:
Gene expression in whole embryos during neurulation.

2. Expression pattern predictions:
2.1: Ectodermal expression pattern prediction for two genes input
as query. 2.2: List of most correlated genes based on WGCNA. 2.3:
*in situ*
hybridization data if available from Xenbase (xenbase.org).

3. Mean expression per dissected region:
average expression for each dissected region for multiple query genes.

4. Co-expression network:
dynamic synexpression network based on WGCNA for a list of input genes.

  

*All figures can be downloaded as png (right click or drag'n'drop).*

  

### **Gene input panel**

This long tab will serve as gene query inputs. Please read the descriptions of each of the sections on the right tabs.

When EctoMap is launched, please be patient and wait
until all data is loaded (bmp4 should appear as the default gene name in
the first input location). Any gene among the 31000 genes expressed in
the ectoderm dataset can be queried, including X. laevis homeologous
copies. To query a gene, type the human HUGO name and select from the
proposed list.

*\* If needed, expression values can be normalized against either odc, gapdh, ef1a or ddx3.*

*\* Note: Please press [draw predictions] or [submit] after each query.*

  

#### **1.** Time series plot

Input TWO OR MORE gene names from list below (Hugo names [X. laevis name - .l or .s]).

bmp4.l[bmp4(0.0)]bmp4.s[bmp4(0.0)]

bmp4.l[bmp4(0.0)]

bmp4.s[bmp4(0.0)]

##### Normalize by:

no normalization

no normalization

Draw predictions

  

#### **2.** Expression pattern predictions

Expression pattern predictions for Stage 12.5 and St. 14.

  

Transcript #1:

bmp4.l[bmp4(0.0)]

bmp4.l[bmp4(0.0)]

Show genes that co-express with Transcript 1 (2.b)

If available, show an example of a published in situ image deposited in www.xenbase.org (2.c)

Transcript #2:

bmp4.s[bmp4(0.0)]

bmp4.s[bmp4(0.0)]

  

Show the expression pattern prediction

Draw predictions

  

#### **3.** Mean expression levels in dissected regions

##### Select the stage to obtain the mean expression values:

12.5

14

##### **Please select the genes you would like to include:**

Include genes plotted in Time Series (section #1)

Include NMF prediction transcripts (section #2)

You can also select genes or transcripts from previous sections.

Show the average FPKM values from the ectodermal tissue dissections (Section 2)

##### Genes names (optional):

  

Submit

  

#### **4.** Co-Expression Network

All the genes input from sections 1 through 4 will
become input nodes (including landmark genes). The number of associated
connected to the input nodes will be determined by the p-value cutoff.
Please give some time for the Network to be made. Please be patient
(several minutes) while the Network is being computed.

Display co-expression Network

###### Landmark genes to include in the network:

Neural crest

Neural border

Ventral

Anterior

Neural Plate

Epidermis

Posterior

Type of correlation

pearson

pearson

Type of correlation

1e-14

1e-14

1e-3

1e-4

1e-5

1e-6

1e-7

1e-8

1e-9

1e-10

1e-11

1e-12

1e-13

1e-14

Submit

  

**Sample codes of dissected regions:**

**ANB/PPE:**
Anterior Neural Border and PrePlacodal Ectoderm (stage 12.5)

**ANF/PPE:**
Anterior Neural Fold and PrePlacodal Ectoderm (stage 14)

**Ec:**
Ectoderm (stage 12.5)

**Eca:**
Ectoderm, anterior

**Ecp:**
Ectoderm, posterior

**NPa:**
Neural Plate, anterior

**NPp:**
Neural Plate, posterior

**NBa:**
Neural Border, anterior

**NBl:**
Neural border, lateral (stage 12.5)

**NBp:**
Neural Border, posterior

**WE:**
Whole Embryo

  
  

EctoMap is a collaborative project between Monsoro-Burq,
Vert, Harland, and Eisen teams (respective main affiliations:
Université Paris Sud, Institut Curie, Orsay, France; Mines ParisTech,
Institut Curie, Paris, France; UC Berkeley, Berkeley, USA; UC
Berkeley/HHMI, Berkeley, USA).

###### Please cite: Plouhinec and Medina-Ruiz et al., 2017 - In revision

  
  
  

##### Send your comments or feedback to:

##### Prof. Monsoro-Burq - anne-helene.monsoro-burq@curie.fr

  

###### Most recent update:

##### 05/2017 - AHMB, JLP, & SMR

- 1. TIME SERIES PLOT
- 2. EXPRESSION PATTERN PREDICTIONS
- 3. MEAN EXPRESSION IN DISSECTED REGIONS
- 4. CO-EXPRESSION NETWORK

Each data point represents the expression level of the
selected transcript (in FPKM) from a single whole embryo, at a given
developmental stage (NF stage, from Nieuwkoop and Faber staging table,
Schemes of embryos during neurulation are presented below the diagram).
This time series allows comparison with other whole embryo time series
such as Yanai et al., 2011.

*The human gene name(s) input from the left panel
will be shown as title. If genes are be duplicated in the X. laevis
genome, each the transcripts will be indicated by a different color as
shown in legend.*

  

**FPKM:**
Fragment Per Kilobase per Million mapped fragment.

- 2.a. Ectodermal expression patterns prediction
- 2.b. List of co-expressed transcripts
- 2.c. In situ hybridization data from Xenbase.

About this section:
Seven regions were dissected from the ectoderm layer of
*Xenopus leavis*
neurulas at Stages 12.5 (early neurula) and 14
(mid-neurula). Each dissected region is color-coded on dorsal and side
views of embryos at each stage. Abbreviations are listed (as well a in
the left side bar).

  

  

### **Expression pattern in the ectoderm at early and mid-neurula stage.**

egions of the ectoderm have been dissected out in
embryos, according to the patterns presented the schemes, at two stages:
12.5 (early neurula) and 14 (mid neurula). Triplicates of individual
samples were sequenced. Expression of each gene in each ectoderm region
is shown based on two approaches: averaging of replicate values for each
dissected region (
**average pattern**
) and NMF-based deconvolution (
**NMF-pattern**
). In each case, the relative intensity of gene
expression is shown in shades of blue: each gene expression is rescaled
between 0 (no expression, white) and 1 (tissue with higher expression,
darkest blue).

  

*Usually, NMF deconvolution resolves better the
pattern of variable genes than average pattern, and allows defining
region-specific genes. However, NMF deconvolution groups some dissected
regions together: posterior neural plate (NPp) and posterior neural
border (NBp) are merged at stage 12.5; anterior (NPa) and posterior
neural plate (NPp) are merged at stage 14. According to your specific
needs, please refer to one or both strategies.*

  

*Please note that rescaling expression levels
for each gene allows detecting expression for weakly expressed genes,
however, it prevents comparing expression levels between stage or genes:
for this, please refer to absolute expression levels in tab 3.*

  

*Please note that posterior tissues (posterior
neural plate, posterior neural border) are closely attached to the
underlying mesoderm in vivo and may be slightly contaminated: please use
the ectoderm enrichment index to select genes highly specific for
ectoderm (btab 3).*

  

##### The diagrams below display the relative ectodermal expression of a given transcript. **1st transcript:** *bmp4.l[bmp4(0.0)]* ***bmp4*** - bone morphogenetic protein 4 Link to Xenbase

##### **2nd transcript:** *bmp4.s[bmp4(0.0)]* ***bmp4*** - bone morphogenetic protein 4 Link to Xenbase

#

**About this section:**
Weighted correlation network analysis (WGCNA) was
used to cluster genes into modules based correlations [Langfelder and
Horvath, 2008].

  

*A signed and unsigned adjacency matrices were
generated using the expression values of all transcripts from both
spatial (dissections) and temporal (dissections and whole embryos)
datasets. The co-expression modules were obtained by introducing the
signed adjacency matrix and the following parameters to the WGCNA
analysis package: soft power = 22, method = hybrid, deepSplit = 4.*

*The 50 transcripts that show closest expression profile similarities with the first transcript input on Section 2 are listed.*

  

Show 51020304050 entries

| bmp4.l[bmp4(0.0)] | Adjacency | Pearson | p-val Pear | Module |
| --- | --- | --- | --- | --- |
| bmp4.l[bmp4(0.0)] | 1.000000000 | 1.0000000 |  | 120 |
| depdc7.l[depdc7(2e-91)] | 0.021625052 | 0.7377136 | 8.881784e-15 | 133 |
| xelaev18029500m.g[lrrc1(0.0)] | 0.019422785 | 0.9030392 | 0.000000e+00 | 52 |
| loc100486773.l[trat1(3e-89)|mrps23(1e-174)] | 0.012417300 | -0.6156268 | 1.561350e-09 | 76 |
| ventx1.1.s[ventx1.1(5e-98)] | 0.012370581 | 0.4617607 | 1.839852e-05 | 138 |
| b3gnt2.l[b3gnt2(0.0)] | 0.012129234 | 0.8240943 | 0.000000e+00 | 133 |
| xelaev18020945m.g[kiaa1324l(0.0)] | 0.011877625 | 0.8752157 | 0.000000e+00 | 120 |
| stx19.l[stx19(0.0)] | 0.011548874 | 0.7724832 | 0.000000e+00 | 73 |
| slc22a31-like.1[slc22a31(0.0)] | 0.009790668 | 0.8060292 | 0.000000e+00 | 68 |
| gfpt1.l[gfpt1(0.0)] | 0.009337031 | 0.7592126 | 4.440892e-16 | 68 |
| gas2l1-like.l[gas2l1(0.0)] | 0.008600356 | 0.8652697 | 0.000000e+00 | 133 |
| xelaev18024443m.g[acp2(0.0)] | 0.008334294 | 0.7905083 | 0.000000e+00 | 133 |
| gfpt1.s[gfpt1(0.0)] | 0.007293724 | 0.7153724 | 1.287859e-13 | 68 |
| bmp4.s[bmp4(0.0)] | 0.006883935 | 0.8254000 | 0.000000e+00 | 133 |
| golph3l.l[golph3l(1e-110)] | 0.006876614 | 0.8144273 | 0.000000e+00 | 50 |
| rbm47.s[rbm47(2e-85)] | 0.006662224 | 0.8208675 | 0.000000e+00 | 68 |
| xelaev18005287m.g[kit(5e-38)] | 0.006481765 | 0.7911903 | 0.000000e+00 | 120 |
| tmem238.l | 0.006168708 | 0.7085703 | 2.771117e-13 | 73 |
| tmem222.l[tmem222(0.0)] | 0.005947328 | -0.6576565 | 4.567458e-11 | 60 |
| xelaev18017523m.g[rassf3(0.0)] | 0.005693392 | 0.8375258 | 0.000000e+00 | 50 |
| loc100145115.l[ensxetg00000023220(0.0)] | 0.005558421 | 0.7829992 | 0.000000e+00 | 73 |
| fam174b-like.l | 0.005428605 | 0.8467313 | 0.000000e+00 | 35 |
| xelaev18022847m.g[kifc3(0.0)] | 0.005141248 | 0.6913557 | 1.749711e-12 | 9 |
| ano5.l[ano5(0.0)] | 0.004941679 | 0.7805820 | 0.000000e+00 | 9 |
| fam83c.l[fam83c(0.0)] | 0.004934477 | 0.7508778 | 1.554312e-15 | 50 |
| cuff.34574 | 0.003976311 | 0.8464567 | 0.000000e+00 | 50 |
| xetrov90030431m.l | 0.003965546 | 0.7981566 | 0.000000e+00 | 50 |
| slc35e1.s[slc35e1(0.0)] | 0.003873704 | 0.8247075 | 0.000000e+00 | 120 |
| foxi4.2.s[foxi4.2(0.0)] | 0.003811328 | 0.7013406 | 6.104006e-13 | 52 |
| xelaev18005230m.g | 0.003713925 | 0.8062607 | 0.000000e+00 | 35 |
| xelaev18004558m.g | 0.003552843 | 0.7603287 | 4.440892e-16 | 73 |
| fzd6.l[fzd6(0.0)] | 0.003378940 | 0.8396914 | 0.000000e+00 | 9 |
| xetrov90016634m.s[twsg1(0.0)] | 0.003250192 | 0.8662881 | 0.000000e+00 | 133 |
| zhx2.l[wdr67(0.0)|zhx2(0.0)] | 0.003248670 | 0.7982077 | 0.000000e+00 | 9 |
| xetrov90021454m.l[kiaa0284(0.0)] | 0.003084592 | 0.6326084 | 3.996634e-10 | 68 |
| smad7-like.l[smad7(2e-30)] | 0.002889053 | 0.7877008 | 0.000000e+00 | 68 |
| xelaev18010552m.g[arvcf(0.0)] | 0.002810006 | 0.8672358 | 0.000000e+00 | 68 |
| ventx1.1.l[ventx1.1(0.0)] | 0.002781294 | 0.4968581 | 3.200097e-06 | 138 |
| ckap4.s[ckap4(0.0)] | 0.002584338 | 0.7703094 | 0.000000e+00 | 50 |
| prrg4.s[prrg4(0.0)] | 0.002533264 | 0.7469497 | 2.664535e-15 | 52 |
| perp-like.s[perp(7e-52)] | 0.002474359 | 0.7130502 | 1.678657e-13 | 54 |
| xelaev18032309m.g | 0.002472387 | 0.5838573 | 1.624850e-08 | 107 |
| loc101734746.l[ensxetg00000017689(0.0)] | 0.002361803 | 0.6008269 | 4.800132e-09 | 73 |
| ventx1.2.l[ventx1.2(7e-73)] | 0.002316855 | 0.3853647 | 4.530341e-04 | 138 |
| ggt1.l[ggt1(6e-29)] | 0.002316473 | 0.4404911 | 4.854148e-05 | 107 |
| sdc1.l[sdc1(5e-57)] | 0.002294647 | 0.5538275 | 1.194308e-07 | 120 |
| xelaev18013379m.g[dgka(0.0)] | 0.002194488 | 0.8743173 | 0.000000e+00 | 9 |
| loc100490855.l[ensxetg00000031447(0.0)|ano9(0.0)] | 0.002182370 | 0.7868503 | 0.000000e+00 | 9 |
| xbp1.l[xbp1(2e-118)] | 0.002151773 | 0.7627012 | 4.440892e-16 | 35 |
| rbm47.l[rbm47(0.0)] | 0.002084556 | 0.7956476 | 0.000000e+00 | 35 |

Processing...

Showing 1 to 50 of 51 entries

- Previous
- 1
- 2
- Next

*In situ*
hybridization expression data recorded in Xenbase
(http://www.xenbase.org) at neurula stage are indicated for comparison.
EctoMap's primary use is to predict expression for novel genes with high
differential expression in the ectoderm for future projects. If no
prior expression was recorded in Xenbase, an error message will appear
("argument is of length zero" and "No in situ image available ...").

  

Mid neurula (St. 11-13) from Xenbase

  

Mid neurula (St. 14-17) from Xenbase

**About this section:**
This section is complementary to the pattern predictions
(tab 2.1). It provides additional information about transcript
enrichment in the ectoderm. The graphs show the average transcript
levels (in FPKM) across different ectodermal dissected regions described
below.

  

*Ectodermal enrichment values are ranked from 0-100,
and are represented by a color gradient (orange through green).
Dark-green for an ectodermal enrichment value of 100 means that the
transcript is be enriched in ectoderm compared to whole embryo. Orange
means the transcript is also expressed elsewhere (e.g. endoderm or
mesoderm).*

  

*Ectodermal enrichment was calculated from the ratio
between the mean expression of a given dissected region over the mean
expression in stage-matched whole embryo, and further expressed as
percentage of the highest ectodermal enrichment value for any gene in
the dataset for this region.*

  

*Note:
The dot-plot height can be adjusted at the bottom of the page*

  
  

##### **Abreviation for dissected regions:**

**ANB/PPE:**
Anterior Neural Border and PrePlacodal Ectoderm (stage 12.5)

**ANF/PPE:**
Anterior Neural Fold and PrePlacodal Ectoderm (stage 14)

**Ec:**
Ectoderm (stage 12.5)

**Eca:**
Ectoderm, anterior

**Ecp:**
Ectoderm, posterior

**NPa:**
Neural Plate, anterior

**NPp:**
Neural Plate, posterior

**NBa:**
Neural Border, anterior

**NBl:**
Neural border, lateral (stage 12.5)

**NBp:**
Neural Border, posterior

  


##### Adjust pixel height for Dotplot

3002,500005003005207409601,1801,4001,6201,8402,0602,2802,500

OK

### **4.** Gene Network

  
  

Note: The visualization of the network visualization may take up to 5 min.

xelaev18031998m.g[snai2(0.0)]sox9.s[sox9(0.0)]sox8.l[sox8(0.0)]gdf6.sloc100486554.l[cacng4(0.0)]sh3pxd2a.l[sh3pxd2a(2e-114)]tfap2e.s[tfap2e(2e-125)]sox9.1[sox9(0.0)]prmt5.l[prmt5(0.0)]lmx1b.1.s[lmx1b.1(3e-66)]mthfr.l[mthfr(0.0)]ercc1.l[ercc1(4e-28)]cldnd1.l[cldnd1(5e-95)]loc100492966.l[c3(0.0)]sox10.s[sox10(4e-103)]hook2-like.l[hook2(0.0)]rapgef2.s[rapgef2(0.0)]cirh1a.l[cirh1a(0.0)]hoxb1.l[hoxb1(0.0)]cuff.62960[snai1(1e-130)]supv3l1.s[supv3l1(0.0)]xelaev18043762m.g[slc16a3(0.0)]zic5.s[zic5(0.0)]c3.l[c3(8e-56)]xelaev18015385m.g[snora18(3e-38)|snora32(4e-24)|snora8(5e-50)]sox8.s[sox8(0.0)]aqp3.l[aqp3(0.0)]baz2b.l[baz2b(0.0)]boc.l[boc(0.0)]loc100488246.l[c14orf169(0.0)]twist1.l[twist1(0.0)]xetrov90002209m.1[rcl1(3e-54)]xelaev18033703m.g[snai2(0.0)]loc100498368-like.s[pcdh8l(0.0)]twist1.s[twist1(0.0)]loc100498368-like.l[pcdh8l(0.0)]xelaev18009061m.g[pdgfra(0.0)]xelaev18005286m.g[pdgfra(0.0)]itga4.l[itga4(1e-74)]rapgef2.l[rapgef2(0.0)]xetrov90003391m.sphf3.l[phf3(0.0)]xelaev18001718m.g[polr3a(0.0)]rhebl1.s[rhebl1(4e-126)]mettl9.l[mettl9(0.0)]loc100494497.1[map1b(4e-120)]ccdc88a-like.s[ccdc88a(3e-106)]emilin1.l[emilin1(5e-68)]rhebl1.l[rhebl1(2e-109)]itga4.s[itga4(5e-73)]loc100485037-like.lxelaev18044445m.g[myo10.2(0.0)]pprc1-like.l[pprc1(2e-22)]kal1.l[kal1(8e-47)]sox10.l[sox10(3e-78)]adam11.l[adam11(2e-85)]xelaev18044261m.g[xb-gene-959703(9e-101)]tdgf1.3.s[tdgf1(1e-144)]stard13.s[stard13(5e-111)]tfap2b.s[tfap2b(0.0)]xelaev18037818m.g[kras(0.0)]naa25.l[naa25(0.0)]mafb.l[mafb(8e-154)]xelaev18039722m.g[ckb(0.0)]xelaev18038233m.g[oct91(0.0)]xelaev18036211m.gosgep.l[osgep(0.0)]mdh2.l[mdh2(0.0)]pex19.l[pex19(0.0)]zmat5.l[zmat5(6e-153)]ssna1.l[ssna1(8e-117)]map2k1.l[map2k1(0.0)]ccnb2.l[ccnb2(2e-166)]wnt1.s[wnt1(0.0)]loc100170576.l[ensxetg00000004330(1e-74)]xelaev18038882m.g[ube2a(0.0)]rasd1.l[rasd1(0.0)]sra1.l[sra1(0.0)]xelaev18030360m.g[ensxetg00000030535(0.0)]dis3l.l[dis3l(0.0)]tmem127.s[tmem127(7e-50)]mxd3.s[mxd3(0.0)]xetrov90019679m.l[oct25(0.0)]hsd17b10.s[hsd17b10(0.0)]xelaev18041755m.g[golga2(1e-58)]ocln.l[ocln(9e-118)]loc100487796-like.sarhgap28-like.1[arhgap28(3e-161)]ocln.s[ocln(2e-85)]marveld2.l[marveld2(0.0)]tfap2b.l[tfap2b(0.0)]xelaev18018155m.g[bmp7.2(0.0)]ube3a.s[ube3a(2e-53)]loc101732978.s[snx17(1e-125)]xelaev18017218m.g[csnk1a1(0.0)]atg16l1.s[atg16l1(1e-93)]cltb.s[cltb(0.0)]xelaev18037330m.g[ets1(3e-116)]hgf.l[hgf(0.0)]cmahp.l[cmahp(1e-58)]cdk9.1[cdk9(0.0)]xelaev18010901m.g[f2rl1(0.0)]anxa9.l[ensxetg00000031026(3e-81)]slc25a30.l[slc25a30(4e-102)]loc100486344.s[notch2(0.0)]xelaev18013641m.g[gpd1(0.0)]xetrov90012209m.s[gpsm2(0.0)]loc100124848-like.l[cald1(0.0)]gja7.l[xb-gene-5905096(0.0)]znf706.s[znf706(1e-105)]tdgf1.3.l[tdgf1(2e-48)]hapln3.l[hapln3(0.0)]ccna2.l[ccna2(8e-120)]xelaev18037929m.g[ensxetg00000018943(4e-104)]rttn.sg2e3.l[g2e3(0.0)]ednra.s[ednra(2e-30)]xelaev18044254m.gvgll1.s[vgll1(0.0)]alg9-like.l[alg9(0.0)|fdxacb1(4e-58)]xelaev18032647m.g[znf585b(1e-62)]xetrov90008749m.lxelaev18032864m.g[nrp1(0.0)]tnfrsf19.s[tnfrsf19(0.0)]mzt1.ssmagp.l[xb-gene-5871173(6e-76)]c10orf58.l[c10orf58(2e-122)]loc100489839-like.l[ensxetg00000008225(8e-74)]gdf6.l[gdf5(4e-59)]psen2.s[psen2(0.0)]cuff.6834[ubc(3e-39)]xelaev18034827m.gslc25a13.s[slc25a13(2e-36)]loc100487248-like.l[plin3(0.0)]cuff.9430[foxd3(2e-166)]foxd3.s[foxd3(0.0)]foxd3.l[foxd3(7e-85)]egr4.l[egr4(2e-94)]spns2.l[spns2(0.0)]xelaev18025735m.g[pfkfb4(0.0)]tmem189.l[tmem189(0.0)]sacs.l[sacs(0.0)]mafb.s[mafb(1e-131)]mmp14.l[mmp14(0.0)]sh3bp4.l[sh3bp4(0.0)]sh3bp4.s[sh3bp4(0.0)]loc100486261-like.l[ccdc61(2e-40)]xelaev18032361m.g[myc(0.0)]ror1.l[ror1(0.0)]herpud1.s[herpud1(5e-108)]cmtm5.l[cmtm5(4e-127)]lbh.l[lbh(3e-120)]wnt1.l[wnt1(0.0)]cdh6.l[cdh6(0.0)]loc100486548.l[lrrc61(0.0)|zbed6(9e-150)]sec23b.l[sec23b(8e-174)]xelaev18046477m.gsh3pxd2a.s[sh3pxd2a(1e-97)]loc101731638.l[ensxetg00000032907(0.0)]mutyh.l[mutyh(0.0)]xelaev18024984m.g[katnb1(2e-129)]xelaev18027783m.g[pax3(9e-173)]xelaev18029860m.g[pax3(2e-149)]xelaev18035571m.g[hes4(0.0)]snx22.l[snx22(0.0)]xelaev18025658m.g[dnal4(2e-136)]bmpr1b.s[bmpr1b(2e-105)]xelaev18019139m.g[arrb2(5e-72)]cuff.50688ift46-like.1[ift46(5e-71)]xetrov90003833m.l[nudt2(6e-124)]zic1.l[zic1(0.0)]dpysl4.s[dpysl4(0.0)]loc100486686.s[sgk223(1e-147)]sptlc1.l[sptlc1(0.0)]wdr78.s[wdr78(0.0)]c6orf62.s[c6orf62(0.0)]loc101732042-like.l[tnk1(0.0)]gdf10.l[gdf10(0.0)]zic5.l[zic5(8e-178)]gse1-like.l[kiaa0182(0.0)]lmx1b.1.l[lmx1b.1(0.0)]pawr.s[pawr(4e-40)]xetrov90010731m.l[lonp2(0.0)]pim3.s[pim3(2e-166)]xelaev18024560m.g[rassf10(0.0)]rsph9.l[rsph9(0.0)]cuff.6143cuff.61231[mars2(8e-121)]xelaev18030771m.g[enkur(0.0)]xetrov90017474m.l[dnttip2(3e-102)]cuff.23967xelaev18022848m.g[katnb1(0.0)]xelaev18004443m.g[ccdc151(0.0)]zic1.s[zic1(2e-170)]zic3.s[zic3(0.0)]pkdcc.1.l[pkdcc.1(2e-29)]zic3.l[zic3(0.0)]stmnd1.s[stmnd1(4e-109)]loc100489393.l[xb-gene-5937787(0.0)]dnai2.s[dnai2(0.0)]c6orf165.l[c6orf165(0.0)]rsph6a.l[rsph6a(0.0)]dzank1.l[c20orf12(0.0)]ccdc37.l[ccdc37(0.0)]olig3.s[olig3(0.0)]trmt6.s[trmt6(0.0)]xelaev18036722m.g[znf384(1e-34)]wdr78.lrsph3.l[rsph3(0.0)]xelaev18006256m.g[arid3a(0.0)]prr18.susp39.l[usp39(0.0)]mak.l[mak(0.0)]ccdc108.l[ccdc108(1e-50)]cenpi.l[cenpi(1e-110)]fam154b.l[fam154b(7e-95)]loc100124772.s[rab6a(1e-25)]ttc18-like.l[ttc18(0.0)]loc100489385.l[rsph1(0.0)]armc4.s[armc4(0.0)]loc101730452.l[azi1(6e-41)]xelaev18026743m.g[ccdc170(0.0)]kiaa1731.1xelaev18019577m.gcnot2.l[cnot2(3e-92)]cdkn2aip.s[cdkn2aip(2e-150)]ss18.s[ss18(7e-176)]spag6.l[spag6(0.0)]ttc24.lsmu1.s[smu1(1e-111)]xelaev18036047m.g[rras(0.0)]xelaev18022884m.g[wdr63(0.0)]loc100170502.l[spag17(0.0)]xelaev18046590m.g[tgif2(4e-137)]xelaev18012138m.g[pdik1l(0.0)]hmgb3.l[hmgb3(0.0)]spata18-like.l[spata18(0.0)]xelaev18043492m.g[tfap2c(0.0)]xelaev18046210m.g[tfap2c(0.0)]kctd15.l[kctd15(0.0)]kctd15.1[kctd15(3e-87)]loc101731508-like.s[mansc1(2e-67)]tmcc3.l[tmcc3(2e-96)]snai1.s[snai1(2e-107)]slc35b2.l[slc35b2(0.0)]zdhhc21.s[zdhhc21(0.0)]gli1.l[gli1.1(1e-80)]loc394967.l[ensxetg00000000927(0.0)]ddx49.1[ddx49(0.0)]rnf2.s[rnf2(0.0)]foxa4.l[foxa4(4e-43)]mex3b.s[mex3b(4e-87)]cuff.11410xelaev18037313m.g[st3gal4(2e-62)]xelaev18042169m.g[sox3(0.0)]xelaev18038842m.g[sox3(0.0)]xelaev18033390m.g[tfap2a(0.0)]grpel2.s[grpel2(4e-44)]gli1.s[gli1.1(0.0)]cuff.3532ptch2.s[ptch2(0.0)]pgrmc2.l[pgrmc2(0.0)]loc100492804.lzc4h2.l[zc4h2(0.0)]mxi1.s[mxi1(0.0)]loc100493716.1[ensxetg00000033891(0.0)|msantd2(0.0)]fam177b.l[galnt11(2e-68)]xelaev18026502m.g[hspc159(6e-105)]xelaev18038168m.gpcdh7.l[pcdh7(0.0)]vill.s[vill(0.0)]msx2.l[msx2(0.0)]msx2.s[msx2(0.0)]msx1.l[msx1(0.0)]dact1.s[dact1(0.0)]prc1.l[prc1(2e-60)]kremen2.l[kremen2(0.0)]pros1.s[pros1(9e-65)]tcf7l1.s[tcf7l1(0.0)]hnf1b.l[hnf1b(0.0)]xarp-like.s[xarp(0.0)]axin2.l[axin2(7e-56)|cep112(0.0)]xelaev18024795m.g[cbfb(2e-58)]tcf7l1.l[tcf7l1(0.0)]xarp-like.l[xarp(0.0)]olig4.l[olig4(0.0)]tcf7.s[tcf7(3e-113)]dhrs3.l[dhrs3(0.0)]kremen2.s[kremen2(0.0)]fzd10.s[fzd10(0.0)]hoxd3.l[hoxd3(0.0)|hoxd4(0.0)]hoxd1.l[hoxd1(0.0)]xelaev18021376m.g[dbn1(0.0)]fzd8.s[fzd8(8e-164)]mgc75753.l[xb-gene-5849780(0.0)]xelaev18010427m.gxelaev18039368m.g[meis3(0.0)]ak3.1[ak3(0.0)]bmp4.l[bmp4(0.0)]xelaev18029500m.g[lrrc1(0.0)]loc100486773.l[trat1(3e-89)|mrps23(1e-174)]ventx1.1.s[ventx1.1(5e-98)]b3gnt2.l[b3gnt2(0.0)]xelaev18020945m.g[kiaa1324l(0.0)]stx19.l[stx19(0.0)]slc22a31-like.1[slc22a31(0.0)]gas2l1-like.l[gas2l1(0.0)]xelaev18024443m.g[acp2(0.0)]gfpt1.s[gfpt1(0.0)]bmp4.s[bmp4(0.0)]golph3l.l[golph3l(1e-110)]rbm47.s[rbm47(2e-85)]xelaev18005287m.g[kit(5e-38)]xelaev18017523m.g[rassf3(0.0)]loc100145115.l[ensxetg00000023220(0.0)]fam174b-like.lxelaev18022847m.g[kifc3(0.0)]ano5.l[ano5(0.0)]fam83c.l[fam83c(0.0)]cuff.34574xetrov90030431m.lslc35e1.s[slc35e1(0.0)]xelaev18005230m.gfzd6.l[fzd6(0.0)]xetrov90016634m.s[twsg1(0.0)]zhx2.l[wdr67(0.0)|zhx2(0.0)]smad7-like.l[smad7(2e-30)]xelaev18010552m.g[arvcf(0.0)]ckap4.s[ckap4(0.0)]perp-like.s[perp(7e-52)]xelaev18032309m.gventx1.2.l[ventx1.2(7e-73)]sdc1.l[sdc1(5e-57)]xelaev18013379m.g[dgka(0.0)]loc100490855.l[ensxetg00000031447(0.0)|ano9(0.0)]rbm47.l[rbm47(0.0)]loc100497331-like.l[tnfsf10l(9e-118)]xelaev18030475m.g[ensxetg00000002717(9e-69)|ensxetg00000010805(1e-120)|ensxetg00000032531(4e-64)]loc100495392.s[has-rs(0.0)]gs17.s[gs17(2e-43)]xelaev18043962m.g[mgc145685(0.0)]xetrov90026849m.l[gripap1(0.0)]hpgd.l[hpgd(1e-60)]gadd45a.l[gadd45a(0.0)]xelaev18014488m.gtsku.s[tsku(0.0)]tll1-like.1.1[tll1(0.0)]xelaev18002058m.g[csnk1d(0.0)]psmc6.l[psmc6(0.0)]xelaev18046261m.gbmp7.1.l[bmp7.1(2e-54)]sgms1.s[sgms1(1e-180)]slc25a1.l[slc25a1(0.0)]cuff.54745xelaev18025905m.g[gata2(6e-179)]bambi.l[bambi(0.0)]xetrov90021782m.l[bub1b(3e-83)]xelaev18020505m.g[mfge8(0.0)]xelaev18039780m.g[sgpp1(0.0)]ism2.l[ism2(0.0)]kitlg.s[kitlg(9e-86)]xelaev18045499m.g[slc9a3r2(2e-142)]rreb1.s[rreb1(0.0)]slc16a1.l[slc16a1(1e-48)]xelaev18025447m.g[uap1(9e-156)]pcdh1.l[pcdh1(0.0)]zfyve28-like.2.s[zfyve28(0.0)]ahnak-like.s[ahnak(1e-25)]slc9a3r2.l[slc9a3r2(0.0)]cuff.39370[mpp1(8e-62)]xelaev18039781m.gxelaev18027940m.g[tf(0.0)]xelaev18031809m.g[rnmt(0.0)]klf5.s[klf5(2e-25)]gata4.l[gata4(0.0)]xetrov90029418m.llyst.l[lyst(0.0)]xelaev18046093m.g[tanc2(4e-53)]gjb1.l[gjb1(0.0)]degs1.l[degs1(0.0)]loc101735152.l[xb-gene-5789558(2e-156)|rbm7(0.0)]xelaev18012140m.g[slc30a2(4e-96)]ppa1.l[ppa1(0.0)]gnai1.l[gnai1(0.0)]mipep-like.l[mipep(0.0)]ralbp1.s[ralbp1(0.0)]loc100497097.l[ensxetg00000032047(8e-165)]xelaev18030881m.g[pon2(2e-161)]loc734035-like.l[znf618(0.0)]cenpe-like.s[cenpe(0.0)]tmbim4.l[tmbim4(3e-160)]hacl1.l[hacl1(0.0)]atg3.s[atg3(0.0)]extl2.l[extl2(0.0)]loc394444.l[xb-gene-5888886(0.0)]slc25a13.l[slc25a13(3e-69)]got1.s[got1(0.0)]sycp2l.1[sycp2l(0.0)]loc100486858.lxelaev18039061m.g[eml1(0.0)]acad10.l[acad10(0.0)]eea1.l[eea1(0.0)]spint2.l[spint2(0.0)]cdkal1.s[cdkal1(0.0)]xelaev18015849m.g[cd63(0.0)]xetrov90019656m.lventx1.1.l[ventx1.1(0.0)]ventx1.2.s[ventx1.2(0.0)]xelaev18036903m.g[ventx2.1(0.0)]xelaev18036901m.g[ventx2.1(0.0)]xelaev18034617m.g[ventx2.1(2e-156)]xelaev18005858m.g[shroom3(7e-73)]xelaev18027350m.g[lrrc1(0.0)]xelaev18029886m.g[farp2(0.0)]xetrov90029418m.scuff.30797dnmt1.s[dnmt1(0.0)]nup93.l[nup93(0.0)]xelaev18019733m.g[has-rs(0.0)]xelaev18019732m.gsgk1.l[sgk1(1e-92)]xelaev18030478m.g[ensxetg00000010805(1e-144)]depdc7.l[depdc7(2e-91)]xetrov90016634m.l[twsg1(0.0)]dock6-like.l[dock7(0.0)]xelaev18023070m.g[nasp(4e-144)]rbbp7.s[rbbp7(0.0)]bzw1.l[bzw1(6e-54)]tiprl.s[tiprl(0.0)]loc100488348.l[c4orf27(0.0)]dcp2.s[dcp2(4e-107)]stk38l.s[stk38l(0.0)]mis18a.siqgap2.l[iqgap2(0.0)]gata4.s[gata4(0.0)]xelaev18026687m.gloc101733741.s[pfkm(0.0)]ggt1.l[ggt1(6e-29)]xelaev18016892m.g[vdac1(7e-147)]xelaev18011585m.gxelaev18024651m.g[xb-gene-5846709(0.0)|xb-gene-5969034(3e-96)]xelaev18010507m.g[vegt(0.0)]tmem222.l[tmem222(0.0)]setd6.l[setd6(0.0)]xelaev18007613m.g[vegt(0.0)]t.l[t(0.0)]xelaev18006074m.g[lmnb2(0.0)]xelaev18034615m.g[ventx2.1(0.0)]hes6.1.s[hes6.1(0.0)]xelaev18008863m.g[tacc3(0.0)]dusp12.l[dusp12(0.0)]ngfr.l[ngfr(1e-50)]sept11.l[sept11(0.0)]xelaev18031753m.g[sall3(0.0)]irf6.s[irf6(2e-151)]foxd4l1.1.l[foxd4l1.1(0.0)]itm2a.s[itm2a(1e-127)]cct4.s[cct4(0.0)]xelaev18033154m.g[trim71(2e-22)]pnhd.s[pnhd(6e-51)]xelaev18032701m.g[ensxetg00000010805(0.0)]wnt8a.s[wnt8a(0.0)]xelaev18001340m.ghes6.1.l[hes6.1(4e-169)]xelaev18037835m.g[nucb1(0.0)]adss.l[adss(0.0)]rrm2.1.l[rrm2.1(0.0)]col4a3bp.l[col4a3bp(0.0)]xelaev18041475m.g[oct91(0.0)]velo1.l[velo1(0.0)]xelaev18038702m.g[irg1(0.0)]mpzl3.l[mpzl3(1e-51)]zpy1.l[zpy1(0.0)]upk3a.l[upk3a(4e-104)]xelaev18038150m.g[cfp(0.0)]xelaev18014128m.g[ensxetg00000031530(1e-43)]ercc3.l[ercc3(0.0)]znf532.l[znf532(1e-75)]loc100488374-like.lxelaev18023995m.g[gata2(0.0)]xelaev18013233m.g[elf1(0.0)]dlx3.s[dlx3(7e-167)]b3gnt2.s[b3gnt2(0.0)]xelaev18015751m.g[elf1(0.0)]cers2.l[cers2(0.0)]slc35c1.l[slc35c1(0.0)]esrp1.l[esrp1(0.0)]grhl3.l[grhl3(1e-87)]als2cl-like.l[als2cl(0.0)]loc100497108.l[elovl3(0.0)]kitlg.l[kitlg(3e-90)]foxi4.1.s[foxi4.1(0.0)]camsap3-like.l[kiaa1543(9e-151)]slc7a4.l[slc7a4(0.0)]lasp1.l[lasp1(0.0)]xelaev18030077m.g[grhl1(5e-167)]xelaev18008287m.g[mcidas(4e-110)]ovol2.l[ovol2(0.0)]loc100485621.l[ensxetg00000034079(0.0)]scamp2.s[scamp2(7e-80)]loc100486704.lxelaev18010956m.g[mcidas(4e-106)]clcn5.l[clcn5(2e-31)]dlx3.l[dlx3(1e-80)]klf5.l[klf5(6e-33)]bambi.s[bambi(0.0)]usp53.s[usp53(0.0)]hunk.l[hunk(0.0)]xelaev18020919m.g[gata3(0.0)]loc100486640-like.l[ensxetg00000031289(0.0)]foxi4.2.l[foxi4.2(0.0)]xelaev18028670m.g[slc30a10(9e-169)]xetrov90026186m.lxelaev18043641m.gloc100486385.sxelaev18028037m.g[grhl1(0.0)]emp2.l[emp2(2e-78)]degs3.s[degs3(0.0)]sptlc2.l[sptlc2(1e-21)]cant1.l[cant1(0.0)]krt8.s[krt8(0.0)]mfsd6l.l[mfsd6l(0.0)]ehd4.l[ehd4(0.0)]gopc.l[gopc(6e-37)]xelaev18013493m.g[ensxetg00000025150(0.0)]loc100488893.szbtb7b.s[zbtb7b(0.0)]krt5.7.l[krt5.7(0.0)]xelaev18046302m.g[slc17a9(5e-55)]mfsd6-like.l[mfsd6(0.0)]rbbp8nl.l[c20orf151(0.0)]esrp1.s[esrp1(0.0)]xelaev18045586m.g[tmem8a(0.0)]nipal3.1[nipal3(2e-50)]xelaev18043807m.g[syngr2(0.0)]capns1-like.1[capns1(0.0)]xelaev18004429m.g[yipf2(3e-141)]xelaev18047992m.gywhaq.s[ywhaq(0.0)]xetrov90003730m.s[atp8b1(1e-69)]cnksr1.l[cnksr1(7e-75)]napepld-like.l[napepld(0.0)]golph3l.s[golph3l(4e-62)]tspan13.s[tspan13(0.0)]cdh1.s[cdh1(8e-40)]jup.s[jup(0.0)]loc100493854.l[ensxetg00000025754(0.0)]xelaev18026315m.g[capn13(5e-44)]xelaev18001338m.g[gdpd3(4e-130)]znf534.l[znf534(3e-142)]xelaev18039840m.g[otx2(0.0)]xelaev18041108m.g[otx2(0.0)]otx1.l[otx1(6e-94)]otx1.s[otx1(3e-35)]dmbx1.s[dmbx1(0.0)]xelaev18001776m.g[hesx1(1e-147)]fezf2.s[fezf2(0.0)]loc101732807.l[masp1(0.0)]pitx2.l[pitx2(0.0)]bcas1.sxelaev18012919m.g[lims1(0.0)]gsc.s[gsc(0.0)]xelaev18046921m.gxelaev18019575m.g[ephb4(0.0)]loc100489393.sfzd8.l[fzd8(0.0)]gsc.l[gsc(0.0)]slc19a1.l[slc19a1(0.0)]fezf2.l[fezf2(0.0)]xetrov90025030m.lsnn.l[snn(0.0)]xelaev18026707m.g[plekhg1(1e-160)]xelaev18037944m.g[ece1(0.0)]sema3a.l[sema3a(0.0)]nuak1.s[nuak1(0.0)]fgfr2.l[fgfr2(0.0)]xelaev18000896m.g[hes7.1(3e-129)]sfrp2.l[sfrp2(0.0)]xelaev18037610m.g[phf13(0.0)]cuff.26459six3.l[six3(0.0)]xelaev18015208m.g[bsdc1(0.0)]xelaev18003045m.g[hesx1(3e-172)]six3.s[six3(0.0)]xelaev18024021m.g[frzb2(0.0)]rax.s[rax(0.0)]fezf1.l[fezf1(0.0)]rax.l[rax(6e-27)]lhx2.s[lhx2(0.0)]lin9.s[lin9(0.0)]crx.l[crx(8e-157)]lhx2.l[lhx2(0.0)]pitx1.l[pitx1(1e-179)]agr2.l[agr2(0.0)]a2m.s[a2m(0.0)]xelaev18046959m.g[cyp27c1(1e-139)]c1galt1.s[c1galt1(0.0)]xelaev18041330m.g[foxn3(0.0)]fzd5.l[fzd5(0.0)]xelaev18007387m.g[smtn(0.0)]dmbx1.l[dmbx1(0.0)]frzb.l[frzb(0.0)]dmrta1.s[dmrta1(0.0)]pitx2.s[pitx2(0.0)]spib.l[spib(0.0)]gatsl1.l[gatsl1(0.0)]xelaev18030463m.g[ensxetg00000011313(6e-54)]ccnd1.s[ccnd1(8e-59)]cuff.41401xelaev18025927m.g[frzb2(0.0)]crx.s[crx(0.0)]nkx3-1.l[nkx3-1(0.0)]xelaev18010379m.g[tbx3(0.0)]dmrta1.l[dmrta1(0.0)]cobll1.l[cobll1(4e-69)]xelaev18007438m.g[tbx3(0.0)]fgfrl1.l[fgfrl1(0.0)]cpn1.s[cpn1(0.0)]agr2.s[agr2(0.0)]xelaev18001054m.g[ephb4(0.0)]hs6st1.l[hs6st1(0.0)]emx1.s[ensxetg00000033848(8e-132)]mmel1.s[mmel1(0.0)]foxd1.l[foxd1(2e-125)]xetrov90011824m.s[faah.3(0.0)]sema3a.s[sema3a(0.0)]sox17a.s[sox17a(0.0)]ckmt1b.s[ckmt1b(0.0)]xelaev18036436m.gszl.s[szl(9e-138)]c8g.sloc100495470.l[ensxetg00000010393(1e-27)]xelaev18045424m.g[ern2(0.0)]slc6a3-like.s[slc6a3(2e-142)]gata6.l[gata6(0.0)]xelaev18029423m.g[tpbg(0.0)]xelaev18020620m.g[myef2(0.0)]foxa2.s[foxa2(4e-40)]fstl1.s[fstl1(1e-55)]srl-like.s[srl(3e-123)]xelaev18003341m.g[hmcn2(3e-71)]xelaev18003703m.gxelaev18002200m.g[atp2a1(0.0)]plod2-like.l[plod2(5e-62)]xelaev18034311m.g[ensxetg00000008230(1e-54)|a2ml1(2e-94)|pzp(0.0)]adap1.l[xb-gene-5838973(0.0)|adap1(1e-44)]xelaev18004064m.g[eef1a1o(0.0)]lefty.s[lefty(0.0)]sparc.l[sparc(2e-74)]xelaev18030588m.g[ensxetg00000006158(6e-38)|obscn(6e-173)]des.1.s[des.1(0.0)]st3gal1.l[st3gal1(0.0)]col5a1-like.l[col5a1(7e-69)]lama1-like.l[lama1(0.0)]srl-like.l[srl(0.0)]nkx6-2.s[nkx6-2(1e-39)]ddit4.l[ddit4(0.0)]xelaev18024361m.g[tnnt3(0.0)]xelaev18010718m.g[lztr1(0.0)]acvr2b.s[acvr2b(0.0)]sox2.1[sox2(0.0)]xelaev18029679m.gcuff.43222xelaev18001880m.gxelaev18003796m.gcuff.45834xelaev18033755m.g[xtr-mir-124(1e-37)]xelaev18005792m.g[dck.1(2e-41)]hes3-like.lzc4h2.s[zc4h2(0.0)]lrig3.s[lrig3(0.0)]hes3-like.sxelaev18014401m.g[pou2f1(0.0)]xelaev18042154m.g[pcdh19(0.0)]ccdc85c.1[ccdc85c(2e-100)]xelaev18010457m.g[msi1(1e-80)]rgma.l[rgma(0.0)]xelaev18017490m.g[metap2(0.0)|usp44(1e-117)]xelaev18016487m.g[pgm2l1(1e-35)]cptp.l[gltpd1(0.0)]xelaev18037374m.g[cadm1(1e-97)]xelaev18009473m.g[dck.1(0.0)]cuff.3842loc101731059.lcetn4.l[cetn4(2e-42)]kdm1a-like.s[kdm1a(0.0)]foxi1.l[foxi1(2e-38)]foxi1.s[foxi1(9e-46)]xelaev18043178m.g[ubp1(0.0)]sox7.s[sox7(1e-59)]loc100488540.l[arpc1a(3e-45)]atp6v1c2.l[atp6v1c2(0.0)]xelaev18016678m.g[ensxetg00000002678(4e-51)]loc100494515-like.l[mb(0.0)]cdc20b-like.l[cdc20b(1e-34)]xelaev18047022m.g[ensxetg00000019246(0.0)|ensxetg00000019254(0.0)]xelaev18030782m.g[msrb2(5e-57)]slc2a12.l[slc2a12(0.0)]tbcel.l[tbcel(0.0)]ccdc67.l[ccdc67(0.0)]loc100216141.sefhc1.l[efhc1(0.0)]xelaev18014036m.g[sytl2(3e-37)]klf6.lloc100497528.l[ensxetg00000019246(0.0)]cast.s[cast(6e-37)]xelaev18020207m.g[slc18a1(4e-62)]xelaev18021045m.g[ndufa5(1e-165)]cuff.47709bcar3.l[bcar3(0.0)]mmp3.l[mmp3(0.0)]hk2.s[hk2(0.0)]speg.s[ensxetg00000019246(3e-55)|speg(3e-23)]rhof.l[rhof(0.0)]rab15.l[rab15(0.0)]slc8b1.l[slc24a6(2e-68)]ptk2b.s[ptk2b(2e-177)]loc101731705.l[tbata(2e-29)]ca2.l[ca2(0.0)]ccno.l[ccno(0.0)]xelaev18029603m.gaim1-like.1.s[aim1(0.0)]loc100493614.l[krt18(0.0)]xelaev18009066m.g[kit(9e-41)]tp63.s[tp63(5e-53)]foxi4.1.l[foxi4.1(6e-41)]hs6st1.s[hs6st1(1e-130)]eya1.1[eya1(0.0)]loc100493941-like.l[tbc1d24.2(5e-78)]tmprss4.l[tmprss4(7e-85)]pou2f3.l[pou2f3(2e-47)]bpgm.l[bpgm(0.0)]epas1.s[epas1(0.0)]ptger4.s[ptger4(7e-100)]xelaev18039754m.gxelaev18019196m.g[ensxetg00000004003(0.0)]foxi4.2.s[foxi4.2(0.0)]cxcl12.s[cxcl12(2e-102)]scnn1b.s[scnn1b(0.0)]gfpt1.l[gfpt1(0.0)]xelaev18007708m.g[arvcf(0.0)]fsbp-like.s[fsbp(4e-157)]xelaev18032660m.g[mgc145518(0.0)]rnf220.1.l[rnf220.1(0.0)]xetrov90018174m.l[st14(0.0)]cdcp1.l[cdcp1(0.0)]acss2-like.l[acss2.1(0.0)]prrg4.s[prrg4(0.0)]xetrov90011380m.l[ensxetg00000020037(6e-93)]ralbp1.l[ralbp1(0.0)]hoxa1.l[hoxa1(1e-54)]hoxa1.s[hoxa1(0.0)]hoxc5.s[hoxc5(0.0)|hoxc6(0.0)]xelaev18022177m.ghoxc4.l[hoxc4(2e-173)]hoxa3.l[hoxa3(0.0)]hoxb6.l[hoxb6(6e-57)]hoxc5.l[hoxc5(0.0)|hoxc6(0.0)|hoxc8(0.0)]hoxa3.s[hoxa3(0.0)|hoxa7(1e-114)]hoxb3.s[hoxb3(0.0)]hoxb6.s[hoxb6(0.0)]xelaev18013563m.g[hoxc6(0.0)]hoxa7.s[hoxa7(0.0)]hoxb7.l[hoxb8(0.0)|hoxc6(2e-41)]hoxb8.s[hoxb8(0.0)]hoxa9.s[hoxa9(2e-109)]hoxc8.s[hoxc8(0.0)]hoxa7.l[hoxa7(2e-119)]hoxa9.l[hoxa9(0.0)]pomgnt2.l[c3orf39(0.0)]xelaev18043697m.g[ensxetg00000030079(4e-103)]xelaev18045992m.g[hoxb9(3e-114)]cd276.l[cd276(0.0)]rara.s[rara(0.0)]cuff.10197ngfr.s[ngfr(1e-131)]cuff.11990cuff.8230cuff.9001[ensxetg00000030701(5e-143)]
