# Supplementary material for: A molecular atlas of the developing ectoderm defines neural, neural crest, placode, and nonneural progenitor identity in vertebrates
Source: PLoS Biol. 2017 Oct 19;15(10):e2004045. doi: 10.1371/journal.pbio.2004045 (PMC5663519; doi:10.1371/journal.pbio.2004045)
Supplement: S1 Web Archive — (ZIP) [file pbio.2004045.s015.zip › EctoMAP_example-bmp4/EctoMap_tool-bmp4_files/bootstrap.html]

# Not Found
